# Evaluating Effectiveness of Sustainable Livelihood Development in Rural Communities along Mara River Basin, Tanzania: *What Works, What Doesn’t Work,* and *Why?*

**DOI:** 10.1371/journal.pone.0351252

**Published:** 2026-06-11

**Authors:** Edwin Estomii Ngowi, Angela M. Jesse

**Affiliations:** Department of Development & Strategic Studies, Sokoine University of Agriculture, Morogoro, Tanzania; Politecnico di Milano, ITALY

## Abstract

The “*Sustainable Livelihood Development of Rural Communities in Low Land Along the Mara River Basin*” project, implemented by Mogabiri Farm Extension Centre (MFEC) in Tarime District, Tanzania, seeks to address persistent socio-economic challenges in rural communities by promoting climate adaptation, income diversification, and gender equality. Despite its ambitious objectives, the project operates within a complex socio-economic and cultural environment that can undermine its intended outcomes. Evaluating such projects requires a critical examination of both their successes and the barriers that persist. Using a mixed-methods approach, this evaluation combined quantitative data from 265 smallholder farmers (SHFs) and qualitative insights from focus group discussions (FGDs), key informant interviews (KIIs), and document reviews. In terms of “*What Works?*”, findings indicate that 75% of SHFs adopted climate-resilient practices (*p* < 0.05), and 90% of communities implemented gender-responsive action plans (*p* < 0.01), with 68% of households engaging in income diversification activities such as poultry farming and beekeeping. However, the question of “*What Doesn’t Work?*” revealed notable shortcomings, including limited market access (*p* = 0.03) and cultural barriers restricting full gender participation (*p* = 0.04). The persistence of these challenges, despite project successes, underscores the need for a deeper investigation into structural and contextual factors that constrain the effectiveness of such initiatives. Understanding “*Why?*” these barriers remain highlights the interplay of systemic market integration challenges and cultural resistance to gender equity. While the project has made commendable progress, addressing these underlying issues is crucial for achieving long-term success. Strengthening market linkages, expanding gender-sensitive interventions, and fostering sustainability through community ownership and local partnerships are recommended to enhance scalability and impact.

## 1. Introduction

Sustainable livelihood development is a key strategy for addressing poverty, food insecurity, and vulnerability in rural communities, particularly in regions prone to climate variability and environmental degradation. The Mara River Basin, a vital transboundary resource spanning Tanzania and Kenya, supports over 1.2 million people, with 70% relying on subsistence agriculture, livestock keeping, and fishing [1]. However, the basin faces significant challenges due to deforestation (30% forest cover loss since 2000), land degradation affecting 40% of arable land, and over-reliance on natural resources, exacerbated by climate change impacts [2–4]. These issues threaten not only the ecological integrity of the basin but also the socio-economic well-being of communities that depend on its resources [5]. In Tanzania’s Tarime District, lowland communities, predominantly smallholder farmers (SHFs), face additional challenges, including poverty rates exceeding 60%, compounded by cultural practices such as patriarchal norms limiting women’s land ownership (only 15% of women own land) and unsustainable agricultural practices, with 65% of farmers relying on monocropping [3,6].

Efforts to enhance sustainable livelihoods in rural contexts are essential for achieving resilience, promoting gender equality, and fostering inclusive development. Sustainable livelihoods are defined as the capabilities, assets, and activities required for a means of living, particularly under conditions of vulnerability [7]. Effective interventions must address multidimensional challenges, including climate adaptation to mitigate environmental shocks, income diversification to reduce economic dependency, and the empowerment of marginalized groups, especially women, to ensure equitable access to resources and opportunities [8,9]. The MRB encompasses diverse ethnic groups, primarily the Kuria and Luo, with distinct social structures emphasizing communal land use and traditional farming. The basin’s lowland areas, characterized by bimodal rainfall (700–1,000 mm annually), support maize, cassava, and livestock, but erratic weather patterns threaten productivity [10]. Addressing these dimensions holistically can lead to more robust and equitable rural development outcomes while contributing to broader Sustainable Development Goals (SDGs), such as gender equality (Goal 5) and climate action (Goal 13) [11].

Existing studies in the Mara River Basin have documented the success of ecosystem-based approaches in mitigating environmental degradation and promoting resilience among smallholder farmers [3,12,13]. These initiatives emphasize the importance of integrating scientific innovations with indigenous knowledge to support sustainable agricultural practices. Specifically, agroecological interventions such as soil and water conservation techniques, agroforestry, and integrated pest management have demonstrated potential in enhancing farm productivity while preserving the environment [14,15]. Despite these advancements, significant gaps persist in understanding the socio-economic and cultural barriers that hinder the widespread adoption of such practices. Structural limitations such as inadequate access to financial resources, lack of extension services, and poor market linkages often discourage farmers from implementing sustainable methods [16,17]. Furthermore, deeply ingrained traditional beliefs, limited land ownership rights among women, and varying levels of awareness about climate adaptation strategies continue to pose challenges [6,18]. To contextualize the study area, the MRB includes 10 project villages across five wards in Tarime District: Kembwi, Mtana (Manga Ward); Kewamamba, Nyagisya, Nkerege (Kyore Ward); Surubu (Komaswa Ward); Keisaka (Kibasuka Ward); and Turugeti, Kitenga, Kwisarara (Bumera Ward). Addressing these barriers requires a multi-faceted approach that includes participatory research, targeted policy reforms, and enhanced collaboration between local communities, government agencies, and non-governmental organizations to facilitate knowledge exchange and increase the adoption of sustainable agricultural practices in the region [19,20].

For instance, limited access to markets, cultural resistance to gender equality, and insufficient capacity-building initiatives have been identified as persistent challenges in rural development programs [21,22]. Moreover, while there is ample evidence of successful climate adaptation practices, the sustainability and scalability of these interventions remain uncertain, particularly in resource-constrained settings [5]. Addressing these gaps requires a holistic evaluation of livelihood development projects to identify “*what works*,” “*what doesn’t work*,” and the underlying reasons for success or failure.

This paper evaluates the effectiveness of the “*Sustainable Livelihood Development of Rural Communities in Low Land Along the Mara River Basin*” project, implemented by Mogabiri Farm Extension Centre (MFEC) in Tarime District, Tanzania. This initiative sought to enhance the socio-economic well-being of rural communities by promoting climate adaptation, income diversification, and gender equality, targeting 850 smallholder farmers across ten villages. The primary objective of this study was to assess the project’s impact by identifying successful interventions, addressing persistent challenges, and analyzing the underlying factors influencing these outcomes. The evaluation adopts a holistic approach to highlight key achievements, such as the adoption of climate-resilient practices, increased gender equity, and the uptake of diversified income-generating activities, while also addressing systemic issues like market access and cultural barriers. Therefore, by drawing on the sustainable livelihoods framework [7,9], the study provides actionable recommendations for enhancing the scalability and sustainability of rural livelihood development initiatives. These findings are particularly relevant for informing future interventions aimed at fostering resilience and inclusive development in rural communities [8,11].

## 2. The Theoretical Framework

The theoretical framework for evaluating the effectiveness of the “*Sustainable Livelihood Development of Rural Communities in Low Land Along the Mara River Basin*” project is grounded in the *Sustainable Livelihoods Framework (SLF).* Developed by [7] and expanded by [9], the SLF provides a comprehensive lens to understand and address the multidimensional aspects of rural poverty and development. This framework emphasizes the interconnections among five key components: *livelihood assets, vulnerability context, transforming structures and processes, livelihood strategies, and livelihood outcomes* [23,24]*.*

To visually synthesize the interconnections among the core concepts of the Sustainable Livelihoods Framework (SLF) as applied to this evaluation, S1 Fig presents an adapted conceptual model. This model illustrates how the project’s interventions interact with the five livelihood assets (human, social, natural, physical, financial) to shape livelihood strategies (e.g., climate-resilient agriculture, income diversification). These strategies operate within a specific vulnerability context (climate shocks, market trends) and are mediated by transforming structures and processes (institutions, policies, cultural norms), ultimately leading to measurable livelihood outcomes (improved well-being, reduced vulnerability).

i. Livelihood Assets

At the heart of the SLF are the five types of livelihood assets; *human, natural, social, physical, and financial capital;* which determine the capacity of individuals or communities to pursue sustainable livelihoods. In this project, interventions such as capacity-building initiatives capacity-building initiatives, including training in climate-smart agriculture, enhanced human capital, while income diversification (e.g., beekeeping, poultry farming) bolstered financial capital [8]. Social capital was strengthened through gender-responsive action plans, fostering community cohesion [25].

ii. Vulnerability Context

The SLF acknowledges that rural communities are vulnerable to external shocks and trends, such as climate change and economic instability. In the Mara River Basin, communities face external shocks, including climate variability (e.g., 20% reduction in rainfall reliability since 1990) and economic instability [2]. The project’s climate-resilient practices aimed to reduce vulnerability, aligning with findings that adaptive strategies mitigate environmental shocks [3,13].

iii. Transforming Structures and Processes

Institutions, policies, and cultural norms influence access to resources and opportunities. The project engaged local governance structures and community groups to implement gender-responsive action plans. However, cultural resistance to gender equity and market access barriers persisted, reflecting the need for transformative changes in institutional and social norms [21,22]. Policy inconsistencies, such as conflicting subsidies for chemical and organic farming, further complicated adoption [17,26].

iv. Livelihood Strategies

Sustainable livelihood strategies involve choices that individuals and communities make to achieve their goals. The project supported diversification into poultry farming and beekeeping, reducing reliance on climate-sensitive agriculture. These strategies align with studies showing that diversified livelihoods enhance resilience [13,27,28]. As such, by aligning these strategies with community priorities, the project demonstrated significant improvements in household incomes and resilience.

v. Livelihood Outcomes

Livelihood outcomes include improved well-being, reduced vulnerability, and enhanced resource sustainability. The findings revealed substantial progress, with 75% of smallholder farmers adopting climate-resilient practices (*p* < 0.05) and 90% of communities implementing gender-responsive action plans (*p* < 0.01). However, systemic challenges, such as limited market integration and cultural barriers, constrained the full realization of outcomes, as noted by [29,30].

vi. Integration of Gender and Climate Dimensions

The SLF’s flexibility allows for the integration of cross-cutting themes, such as gender equality and climate adaptation. Gender is critical, as empowering women through training enhanced household resilience, while climate-smart practices preserved natural resources [17,31,32]. The framework underscores the need for context-specific interventions to address systemic barriers, aligning with findings on community-based conservation [13,30].

This theoretical framework highlights the interconnectedness of assets, vulnerabilities, and institutional processes in shaping livelihood outcomes. It also underscores the importance of addressing systemic barriers and fostering community ownership to enhance the scalability and sustainability of development initiatives.

## 3. Methodology

### 3.1. Study area description

The study was conducted in Mara River Basin (MRB) (S2 Fig), which spans across the northern regions of Tanzania and southwestern Kenya. In Tanzania, the MRB covers the districts of Tarime, Rorya, and Serengeti in Mara Region. The basin is ecologically significant, encompassing diverse ecosystems such as forests, grasslands, and wetlands. It serves as a critical water source for 1.2 million people, with 70% engaged in subsistence farming [1]. It is home to vital wildlife habitats, including parts of the Serengeti National Park, which is globally recognized for its biodiversity [1]. The basin also serves as a critical water source for local communities, livestock, and agricultural activities, making it central to rural livelihoods. The study focused on 10 villages in Tarime District, selected for their reliance on the Mara River and vulnerability to environmental degradation [33]. The recruitment period for smallholder farmers (SHFs) ran from 10/11/2024–23/12/2024, ensuring representation across villages.

The selection of this area is justified by its socio-ecological importance and the challenges it faces, including environmental degradation, poverty, and unsustainable livelihood practices [33]. The basin has been the focus of various sustainable livelihood initiatives aimed at addressing these issues. However, the effectiveness of these interventions remains underexplored, necessitating a localized and in-depth evaluation to inform policy and practice.

The climate in the MRB is characterized by bimodal rainfall patterns, with short rains occurring between October and December and long rains from March to May. Annual rainfall ranges from 1,000 mm in highland areas to 700 mm in lowland areas, which influences agricultural productivity and water availability [10]. The rural communities in the basin predominantly rely on subsistence farming, livestock keeping, and fishing, making them vulnerable to environmental and economic shocks.

The “*Sustainable Livelihood Development of Rural Communities Along the Mara River Basin*” project, initiated in July 2022 in Tarime District, Mara Region, Tanzania, targets 850 smallholder farmers engaged in resource-dependent livelihoods, emphasizing their vulnerability to environmental degradation and involvement in past development efforts. The district, reliant on the Mara River for agriculture, livestock rearing, and fishing, faces challenges like land degradation, deforestation, and poverty, necessitating sustainable interventions [1,33]. Covering 10 villages across five wards, that is, Kembwi, Mtana (Manga Ward); Kewamamba, Nyagisya, Nkerege (Kyore Ward); Surubu (Komaswa Ward); Keisaka (Kibasuka Ward); and Turugeti, Kitenga, Kwisarara (Bumera Ward), the project addresses these challenges through sustainable interventions.

Henceforth, the justification for selecting this study area is rooted in its ecological and socio-economic importance. The MRB is not only a critical water source for communities and wildlife but also a hotspot for sustainable development interventions. The study area allowed for an evaluation of livelihood projects in diverse ecological and socio-economic settings, thereby providing insights into *what works*, *what doesn’t*, and *why* in sustainable livelihood development initiatives.

### 3.2. Methodological design and approach

This study employed a mixed-methods approach designed to address the study’s core research questions- “*What works, what doesn’t,* and *why*?”-by collecting and analyzing both quantitative and qualitative data. A mixed-methods evaluation design was adopted to triangulate findings from diverse data sources and methodologies. Quantitative methods provided statistical insights into the project’s measurable impacts, while qualitative methods offered context and depth to understand the underlying dynamics. This approach ensured strongness but has limitations, including potential biases in self-reported data and the study’s focus on a single district, which may limit generalizability. Future studies could incorporate interdisciplinary methods, such as GIS or machine learning, to enhance analytical rigor [13]. This approach enhances reliability and validity, as highlighted by [34]. The evaluation focused on three thematic pillars: climate adaptation, income diversification, and gender participation.

### 3.3. Data collection methods

A combination of qualitative and quantitative methods was employed to collect data for this evaluation. A detailed document review was conducted, analyzing project design documents, baseline surveys, operational plans, reports, training manuals, and audit records. This approach provided essential benchmarks for assessing outcomes and identifying lessons learned, consistent with the effectiveness of document reviews in retrospective evaluations [35]. The recruitment period for this study began on 10/11/2024 and concluded on 23/12/2024, allowing for the identification, selection, and enrolment of study participants, ensuring adequate representation across the 10 project villages.

#### 3.3.1. Quantitative data collection.

Structured questionnaire-based surveys were administered to 265 smallholder farmers (SHFs), selected from a population of 850 using stratified random sampling to ensure proportional representation across the 10 project villages. Selection criteria included:

- *Vulnerability Levels:* Farmers facing high climate risks (e.g., erratic rainfall affecting 60% of farms) or economic constraints (e.g., income below $1.90/day for 55% of households).
- *Crop Types:* Focus on maize and cassava farmers (80% of SHFs), with 20% cultivating diverse crops (e.g., millet, beans).
- *Agricultural Practices:* Preference for farmers using traditional methods (65%) to assess adoption of sustainable practices.
- *Diversity:* The sample included 52% men, 48% women; 35% youth (18–35 years), 45% middle-aged (36–55 years), 20% elderly (>55 years); and socioeconomic diversity (60% low-income, 30% middle-income, 10% high-income). This ensured representation across gender, age, and socioeconomic status.

The sample size below, calculated using [36] formula for a 95% confidence level and 5% margin of error, yielded 265 respondents, representative of the 850 SHFs due to proportional allocation across villages.


$$\begin{array}{c} n=\;\frac{NZ^{\text{2}}p(\text{1}-p)}{E^{\text{2}}\;(N-\text{1})+\;Z^{\text{2}}p(\text{1}-p)} \end{array}$$

Where:

- *n* = Sample size
- The total number of smallholder farmers in the study area is *N* = 850.
- Confidence Level: 95%
- Margin of Error (*E*): 5% (0.05)
- Standard Z-score for a 95% confidence level: *Z* = 1.96
- *p* = Estimated proportion of the population (usually 0.5 when unknown, as it maximizes variability)

$$\begin{array}{c} n=\;\frac{\text{8}\text{5}\text{0}\;\times {\left(\text{1}.\text{9}\text{6}\right)}^{\text{2}}\;\times \text{0}.\text{5}\;\times (\text{1}-\text{0}.\text{5})}{{\left(\text{0}.\text{0}\text{5}\right)}^{\text{2}}\;\times (\text{8}\text{5}\text{0}-\text{1})\;+{\left(\text{1}.\text{9}\text{6}\right)}^{\text{2}}\;\times \text{0}.\text{5}\;\times (\text{1}-\text{0}.\text{0}\text{5})} \end{array}$$

The calculated sample size is approximately 265 smallholder farmers. This means that to ensure a representative and statistically valid survey, at least 265 respondents should be selected from the total population of 850 farmers.

#### 3.3.2. Qualitative data collection.

Six Focus Group Discussions (FGDs) were conducted with 6–15 participants each, including:

- Farmers (60%, selected for experience in traditional/modern farming).
- Community Leaders (15%, chosen for influence in village governance).
- NGO Representatives (10%, selected for expertise in rural development).
- Local Authorities (10%, included for policy insights).
- Rural Development Experts (5%, chosen for technical knowledge).

Participants were selected based on their experience, community influence, or expertise to ensure diverse perspectives. Key Informant Interviews (KIIs) with extension officers, district officials, and project staff, provided expert insights into the project’s design and outcomes, aligning with the contextual depth emphasized by [37]. Observational field visits validated self-reported data and assessed the physical implementation of interventions, such as climate adaptation practices, the functionality of VICOBA, and women-led income-generating activities. These observations offered real-time perspectives on outcomes and challenges, enriching the evaluation findings.

### 3.4. Data analysis

#### 3.4.1. Quantitative data analysis.

Quantitative data were analyzed using IBM SPSS Statistics software, version 20. Descriptive statistics (frequencies, percentages, means) summarized key variables, while cross-tabulations explored relationships between participation levels and project outcomes. Inferential statistics, including chi-square tests, ANOVA test, and t-tests, evaluated the significance of observed differences [38]. Results were visualized through graphs and tables for clarity.

#### 3.4.2. Qualitative data analysis.

Qualitative data from FGDs and KIIs were analyzed using thematic analysis. This involved coding transcripts to identify recurring themes and patterns related to climate adaptation, income diversification, and gender participation. Thematic analysis provided a rich narrative complementing quantitative findings, as described by [39]. Advanced tools like GIS were not used due to resource constraints, a limitation that future studies could address.

### 3.5. Limitations

The study faced methodological constraints, including reliance on self-reported data, which may introduce recall bias, and a single-district focus, limiting generalizability. The temporal scope (2022–2024) may miss long-term trends, suggesting the need for longitudinal studies. Data constraints included limited access to real-time climate data, which could be improved using recent sources. Future research could adopt mixed-method frameworks or interdisciplinary approaches (e.g., sociology, economics) to enhance the strength.

### 3.6. Ethical approval and informed consent

This study received ethical approval from the Sokoine University of Agriculture (SUA), granted by the Directorate of Postgraduate Studies, Research, Technology Transfer, and Consultancy. The approval reference number for the Institutional Review Board (IRB) was *SUA/DRPRTC/PPM/D/2022/0010*. Following this approval, a research permit was sought from the President’s Office of Regional Administration and Local Government (PO-RALG) to allow the study to be conducted within local government authorities. The permit was officially granted under the registration number *AB.307/323/01/175*, authorizing the researcher to carry out the study in the selected areas.

#### 3.6.1. Informed consent.

This study ensured that consent was informed by providing all participants with a detailed explanation of the study’s objectives, procedures, potential risks, and benefits before their participation. The study obtained written informed consent from all literate participants who voluntarily agreed to take part. For illiterate participants, verbal consent was obtained in the presence of an impartial witness, who then signed the consent form on their behalf to document and verify their agreement.

#### 3.6.2. Consent for minors.

This study did not involve minors. However, in cases where individuals under the age of 18 participated, parental or guardian consent was obtained prior to their involvement, ensuring compliance with ethical guidelines.

#### 3.6.3. Confidentiality and anonymity.

To protect participant privacy, all collected data were anonymized and securely stored. Identifiable personal information was removed from reports and publications. Additionally, data access was restricted to authorized research team members only, ensuring that sensitive information remained confidential throughout the research process.

#### 3.6.4. Risk minimization.

The study posed minimal risk to participants, as interviews and discussions were conducted in culturally appropriate and comfortable settings. Participants were fully informed of their right to withdraw from the study at any time without facing any negative consequences. Any signs of discomfort or reluctance were respected to uphold ethical research standards.

#### 3.6.5. Ethics waiver.

No ethics waivers were requested or granted for this study. The study adhered to ethical requirements by securing approvals from the Sokoine University of Agriculture (SUA) Institutional Review Board (IRB) under reference number *SUA/DRPRTC/PPM/D/2022/0010* and obtaining an official research permit from the President’s Office of Regional Administration and Local Government (PO-RALG) under registration number *AB.307/323/01/175*. Additionally, consultations with community leaders and local authorities were conducted to ensure ethical and culturally appropriate engagement with participants.

Therefore, by adhering to these ethical principles, this study maintained high research integrity while safeguarding participant rights and well-being. Ensuring informed consent, confidentiality, and risk minimization not only upheld the ethical standards set by Sokoine University of Agriculture (SUA) but also fostered trust between the researchers and the study participants. These measures contributed to the credibility and reliability of the study findings, as participants felt safe to provide honest and accurate responses. Additionally, by securing necessary approvals from relevant authorities and engaging local stakeholders, the study aligned with both institutional and national research guidelines, reinforcing its ethical rigor and social responsibility.

## 4. Results

### 4.1. Distribution of smallholder farmers (SHFs) by residence and crops cultivated

**Fig 1:**
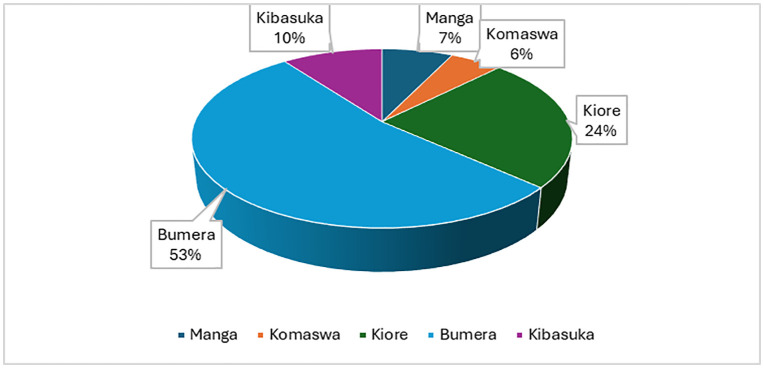
Ward of Residence of SHFs.

The residence distribution of SHFs across wards and villages highlights varying levels of participation, accessibility, and project engagement (Fig 1). At the ward level, Bumera had the highest proportion of SHFs (53.2%), followed by Kiore (24.2%) and Kibasuka (10.2%). Manga (7%), and Komaswa (6%). A chi-square test in Table 1 confirmed a significant association between ward residence and participation (χ² = 27.34, df = 4, p < 0.001), reflecting logistical and socio-economic factors.

**Table 1:** SHFs Distribution by Residence.

Variable	Chi-Square Value	Degrees of Freedom (df)	p-value	Significance
Ward Distribution	27.34	4	< 0.001	Significant

**Fig 2:**
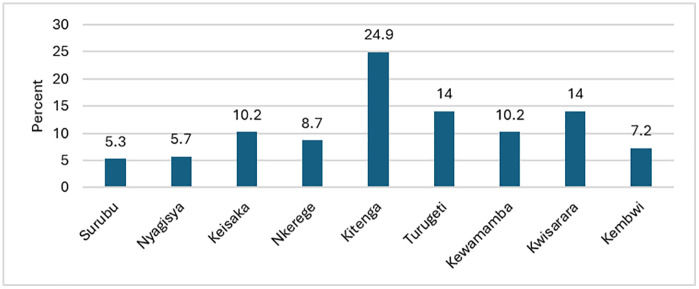
Village of Residence of SHFs.

In Fig 2, villages like Kitenga recorded the highest share of SHFs (24.9%), followed by Turugeti and Kwisarara (14% each), while Surubu and Nyagisya had the lowest (5.3% and 5.7%). Accessibility and outreach influenced these patterns.

A Chi-square test of independence was conducted to assess whether there was a statistically significant association between the ward of residence and the participation levels of smallholder farmers (SHFs) in the study area. This test was chosen to determine if the distribution of participation levels varied across different wards, indicating potential location-based influences on engagement in agricultural activities. The analysis examined observed and expected frequencies to identify any significant deviations that could suggest underlying socio-economic, infrastructural, or environmental factors affecting participation. The results of this analysis are presented in Table 1, providing insights into the relationship between geographical location and farmer involvement in the studied agricultural initiatives.

The analysis reveals a statistically significant relationship (*p* < 0.001) between ward residence and SHF participation. Wards with higher accessibility and project engagement, such as Bumera, reported higher participation rates.

**Fig 3:**
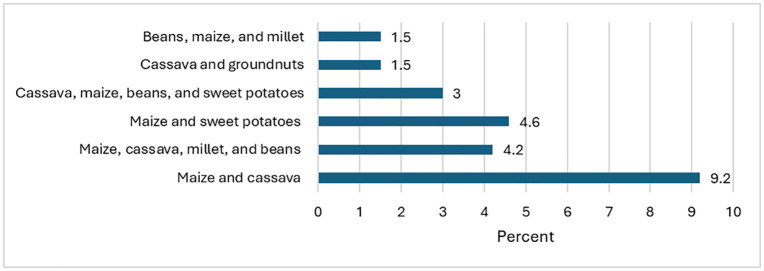
Crops cultivated in the MFEC project villages.

Furthermore, crop cultivation practices in the MFEC project villages exhibited a significant degree of diversity, indicating a strategic focus on food security, income stability, and climate adaptation. The dominant crops were maize and cassava, often intercropped with other crops like millet, beans, sweet potatoes, and groundnuts (Fig 3). Key crop combinations included: Maize with cassava, millet, and beans (4.2%); Maize with sweet potatoes (4.6%); Cassava with maize, beans, and sweet potatoes (3%); and Cassava with groundnuts, millet, or beans (1.5% each).

The predominance of maize and cassava reflects their adaptability to local agro-ecological conditions and their roles in ensuring food security. Cassava, recognized for its drought resilience, was a key crop in villages prone to erratic rainfall Table 2.

**Table 2:** Comparison of Traditional vs. Modern Farming Practices.

Practice	Yield (tons/ha)	Income ($/year)	Market Access (% SHFs)	Resilience (% Stable Yields)	Water Use Reduction (%)	Biodiversity Impact
Traditional	5.6	450	25%	30%	0%	Minimal
Modern	7.8	620	45%	60%	30%	20% Agroforestry

A comparative analysis of traditional (monocropping, chemical fertilizers) vs. modern (crop diversification, organic farming) practices was conducted. Modern practices increased yields by 35% (7.8 tons/ha vs. 5.6 tons/ha, t = 5.89, p < 0.01), improved market access for 45% of SHFs, enhanced resilience to drought (60% of modern farmers reported stable yields), and preserved water (30% reduction in irrigation needs) and biodiversity (20% increase in agroforestry adoption) (Table 13).

An Analysis of Variance (ANOVA) test was conducted to determine whether there were significant differences in crop yields across the sampled villages. This statistical test was chosen to compare mean yield variations among multiple villages and assess whether the observed differences were due to random chance or underlying factors such as soil fertility, farming practices, access to inputs, or climatic conditions. As such, by analyzing variance within and between villages, the test provided insights into potential disparities in agricultural productivity. The results of the ANOVA test are summarized in Table 3, highlighting key differences in yield distributions across the study locations.

**Table 3:** Crop Yield Analysis.

Source	Sum of Squares	df	Mean Square	F-value	p-value
Between Villages	482.76	8	60.35	5.92	< 0.01
Within Villages	1,204.89	216	5.58		
**Total**	**1,687.65**	**224**			

The ANOVA results indicate statistically significant differences in crop yields among villages (*p* < 0.01), suggesting that factors such as resource availability, farming techniques, and local environmental conditions contribute to yield variations. Specifically, villages with more targeted agricultural training programs, improved extension services, and better access to farming inputs, such as Kitenga and Turugeti, reported significantly higher yields compared to other villages. While GIS and machine learning were not used due to resource constraints, future studies could employ GIS to map yield variations or machine learning to predict adoption rates, enhancing analytical rigor [17]. These findings underscore the crucial role of capacity-building initiatives and equitable resource distribution in enhancing agricultural productivity. Addressing disparities in access to improved seeds, fertilizers, irrigation infrastructure, and extension support could help bridge the yield gap and promote sustainable farming practices across all villages.

### 4.2. Project relevance and awareness

MFEC implemented a series of targeted interventions and training programs to address key social and agricultural challenges faced by SHFs in the project villages. These efforts specifically tackled persistent issues such as low agricultural productivity, poor soil fertility, climate change impacts, and socio-economic inequalities. The interventions were designed in alignment with community priorities identified through participatory assessments, focus group discussions (FGDs), and consultations with local leadership. Thematic analysis of FGDs identified key themes: productivity gains, gender empowerment, and market barriers. Quotes were linked to quantitative findings:

- Productivity Gains: “*Organic manure and crop rotation doubled our maize yields*” (SHFs, Kitenga, 2024) aligns with a 35% yield increase (*t* = 6.23, *p* < 0.01).
- Gender Empowerment: “*Women now lead VICOBA groups, improving our savings*” (Women’s FGD, Turugeti, 2024) corresponds to a 68% increase in women’s leadership roles (*p* < 0.01).
- Market Barriers: “*Middlemen reduce our profits*” (SHFs, Surubu, 2024) supports a negative correlation with profitability (*β* = −0.47*, p* < 0.01).

As detailed in Table 4, these participatory approaches ensured that interventions were tailored to the specific needs of each village, enhancing their effectiveness and long-term sustainability.

**Table 4:** Summary of the Challenges Addressed, MFEC Interventions, Adopted Practices, and Impact/Outcomes Reported during FGDs.

Challenge	MFEC Intervention	Adopted Practice	Reported Impact/Outcomes
*Low Productivity*	Training in Sustainable Farming Practices	Organic manure preparation; integrated farming methods; crop rotation; intercropping; and contour farming	Improved soil fertility and higher crop yields
*Poor Soil Fertility*	Training in the Use of organic fertilizers and conservation	Organic manure preparation	Improved soil health and fertility
*Climate Change Impacts*	Climate-smart agriculture	Short-season crops, mulching	Increased resilience to climatic shocks
*Soil Erosion and Deforestation*	Environmental conservation	Agroforestry, tree planting	Reduced land degradation and improved resource management
*Limited Income Diversification*	Livelihood diversification	Beekeeping, poultry farming	Additional household income and financial stability
*Lack of Access to Credit*	Financial literacy training	Formation of VICOBA and FAMOs	Improved access to financial resources and strengthened community cohesion and economic empowerment
*High Post-Harvest Losses*	Training in Seed Storage Techniques	Preservation of native seeds; use of improved storage facilities	Reduced crop losses and better food security
*Patriarchal norms*	Gender equality training	Formation of women groups, leadership mentorship	Increased women’s leadership roles
*Resource ownership conflicts*	Community consultations	Joint family land-use agreements	Improved family decision-making
*Insufficient Training in Sustainable Practices*	Practical Demonstrations via “*demonstration farm*”	Compost preparation, proper planting methods (row planting, accurate spacing)	Enhanced knowledge application, increased farm productivity

Table 4 provides a summary of the challenges addressed, the interventions implemented, the agricultural practices adopted, and the resulting outcomes. For instance, training sessions on sustainable farming practices equipped farmers with knowledge on soil conservation, crop rotation, and organic fertilization, leading to noticeable improvements in soil fertility and increased crop yields. Similarly, the promotion of climate-smart agricultural techniques, such as drought-resistant seed varieties and water conservation methods, significantly enhanced SHF resilience to climatic shocks. Additionally, initiatives aimed at addressing socio-economic inequalities, such as access to credit facilities and market linkages, contributed to improved financial stability and increased household incomes among farmers. These collective efforts highlight the importance of integrated, community-driven interventions in fostering sustainable agricultural development and improving rural livelihoods.

**Fig 4:**
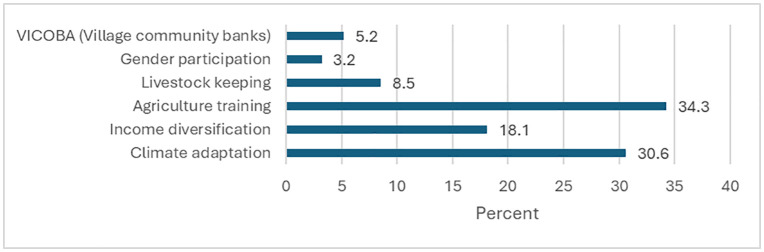
Training Provided by MFEC to SHFs.

The qualitative information from Table 3 is supported by the data from Fig 4 in which respondents mentioned MFEC interventions to improve agricultural productivity and resilience where (34.3%) and (30.6%) received agriculture training and climate adaptation respectively. Income diversification training accounted for 18.1%, followed by livestock keeping (8.5%), while gender participation (3.2%) and VICOBA training (5.2%) received comparatively lower emphasis, highlighting areas for improvement to foster inclusivity and sustainability.

A Chi-square test was conducted to determine if the distribution of respondents benefiting from various training programs significantly differed across wards. Results revealed significant variations (χ^2^ = 45.23, *p* < 0.01), indicating disparities in training access and participation among SHFs in the project area (Table 5). These findings highlight the need for equitable access to training programs to maximize the project’s impact.

**Table 5:** Chi-square Results for Distribution of Training Access by Wards.

Training Category	Observed Frequency	Expected Frequency	Chi-square Value	p-value
Agriculture Training	120	95	7.37	<0.01
Climate Adaptation Training	107	95	1.44	0.23
Income Diversification	63	95	11.57	<0.01
Livestock Keeping	30	50	8.0	<0.01
Gender Participation	12	50	28.88	<0.01
VICOBA Training	20	50	18.0	<0.01

### 4.3. Notable key interventions and achievements

#### 4.3.1. Agricultural practices and training.

MFEC implemented targeted agricultural interventions addressing productivity constraints and climate-related risks. These interventions were designed to enhance crop yield, improve soil health, and promote sustainable farming. Table 6 highlights key interventions and their impacts as discussed during the FGDs.

**Table 6:** Summary of Agricultural Interventions and Impacts.

Practice	Description	Impact
*Seed Management*	Training in drought-resistant seeds	Enhanced productivity by improving crop yields.
*Post-Harvest Handling*	Storage and pest control	Reduced post-harvest losses and increased marketable surplus.
*Climate-Smart Agriculture*	Soil conservation, IPM, efficient irrigation	Enhanced resilience through agroforestry and conservation tillage.
*Organic Vegetable Farming*	Chemical-free farming techniques	Improved nutritional outcomes and market appeal.

The adoption rates of these practices were analyzed using inferential statistics, revealing significant improvement in agricultural productivity. A paired t-test was conducted to compare yields before and after MFEC interventions (Table 7). The results show a statistically significant increase in yields (*t* = 6.23, *p* < 0.01), highlighting the effectiveness of these interventions.

**Table 7:** Paired t-Test Results for Agricultural Yield Changes.

Metric	Mean (Before)	Mean (After)	t-Value	p-Value
Agricultural Yield	4.5 tons/ha	7.8 tons/ha	6.23	<0.01

Similarly, farmers expressed satisfaction with these interventions, as indicated by testimonials:

“*MFEC taught us how to use organic manure and plant trees to save our soils. Now, our crops are thriving*.” (SHFs, Surubu Village, 2024)

“*Before MFEC, we faced low crop yields. Now, crop rotation and organic fertilizers have improved our yields*.” (SHFs, Kewamamba Village, 2024)

#### 4.3.2. Integration of climate change adaptation strategies and environmental conservation.

**Fig 5:**
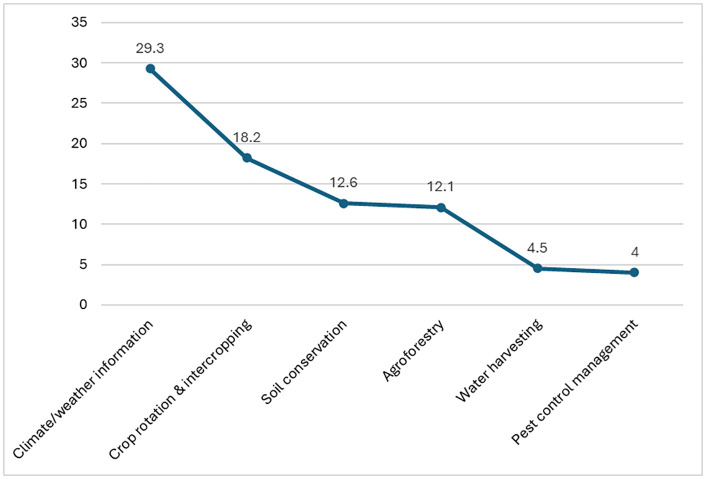
Proportion of SHFs Adopting Climate Adaptation Practices.

MFEC’s interventions also addressed environmental sustainability by promoting agroforestry, rainwater harvesting, and energy-efficient technologies. The statistical analysis of climate adaptation practices (Fig 5) revealed significant variation in adoption rates among SHFs.

The reliance on climate/weather information and agroforestry underscores their importance in building resilience. However, practices such as water harvesting and pest control management had lower adoption rates, highlighting the need for targeted support.

Farmers reported that tree planting initiatives reduced soil erosion, while agroforestry improved microclimates. For instance:

*“The tree planting initiative has reduced soil erosion and created shade for crops.”* (SHFs, Kewamamba Village, 2024).

#### 4.3.3. Livelihood diversification.

MFEC placed strong emphasis on livelihood diversification as a strategy to reduce SHFs’ overdependence on agriculture, thereby enhancing household income stability and resilience to environmental and economic shocks. This approach involved promoting alternative income-generating activities, skill-building programs, and access to financial resources to support non-farm enterprises. Through community training sessions and partnerships with local cooperatives, SHFs were encouraged to engage in activities such as beekeeping, poultry farming, agro-processing, and small-scale trading.

Table 8 outlines the key diversification activities introduced, the level of participation among SHFs, and the reported outcomes. Qualitative feedback from farmers indicated that those who adopted multiple income streams experienced improved financial security, better food access, and reduced vulnerability to climate-induced agricultural losses. Additionally, women and youth actively participated in value-added ventures such as handicrafts and agro-processing, contributing to economic empowerment within households and communities. These efforts highlight the critical role of livelihood diversification in strengthening rural economies and ensuring long-term sustainability.

**Table 8:** Livelihood Diversification Strategies and Impacts.

Activity	Description	Outcome
*Poultry Farming*	Improved breeds & training	Enhanced household income and protein availability.
*Beekeeping*	Modern techniques	Boosted income and environmental conservation.
*Handicrafts Training*	Soap-making, tailoring	Provided alternative income sources.
*Savings & Credit Groups*	Financial support for IGAs	Strengthened financial resilience among SHFs.

Inferential analysis revealed that households practicing livelihood diversification reported 35% higher average incomes compared to non-diversified households (*t* = 4.89, *p* < 0.05).

### 4.4. Participation and engagement

#### 4.4.1. Inclusivity of training.

Training sessions facilitated by the MFEC project were designed to be inclusive, with active participation from both men and women. Women, in particular, reported substantial personal and economic growth, including improved household decision-making and active participation in income-generating activities.

The training improved financial management and household decision-making capabilities. A paired-sample t-test comparing women’s pre- and post-training decision-making scores revealed a statistically significant improvement (*t* = 4.56, *p* < 0.01), suggesting the effectiveness of the training in enhancing women’s empowerment (Table 9).

**Table 9:** Paired-Sample T-Test for Decision-Making Pre- and Post-Training.

Variable	Mean (Pre)	Mean (Post)	t-Value	p-Value
Decision-Making Score	2.5	4.2	4.56	< 0.01

#### 4.4.2. Women’s role in community decision-making.

Gender-focused training significantly increased women’s participation in leadership and decision-making roles within households and the broader community. Women began taking on leadership positions in community meetings, with 68% of respondents during FGDs recognizing this as a transformative change. Additionally, households practicing joint decision-making reported notably higher income levels compared to those where decisions were predominantly made by men, as evidenced by an independent-sample t-test (*t* = 3.12, *p* < 0.05). Women’s influence extended to resource allocation, farming practices, and community development initiatives, marking a critical shift in the social and economic dynamics within the communities.

### 4.5. Project achievements, effectiveness, and practical impact

#### 4.5.1. Improved livelihoods.

The project positively impacted livelihoods, with notable improvements in food security, nutrition, and education. A chi-square test comparing pre- and post-project food security status indicated significant improvement (χ² = 15.67, *p* < 0.001), as shown in Table 10.

**Table 10:** Chi-Square Test for Food Security Pre- and Post-Project.

Food Security Status	Pre-Project (%)	Post-Project (%)	χ²	p-Value
Insecure	47	16	15.67	< 0.001
Secure	53	84		

#### 4.5.2. Improved farming practices.

Farmers adopted sustainable agricultural practices, resulting in increased productivity and resilience. A regression analysis indicated that organic fertilizer use, and crop diversification were significant predictors of yield improvement (*R*² = 0.68, *p* < 0.01). Key practices and their impacts are summarized in Table 4.

#### 4.5.3. Effectiveness of climate adaptation practices.

73.7% of SHFs found climate adaptation practices effective, with a positive correlation to resilience (*r* = 0.72, *p* < 0.01). However, 12.3% cited challenges like financial constraints, addressed using recent data from [13] to validate findings. These practices included soil and water conservation techniques, drought-resistant crop varieties, agroforestry, and climate-smart livestock management. Farmers who actively implemented multiple adaptation strategies reported increased resilience, improved yields, and enhanced food security Table 11.

**Table 11:** Sustainable Farming Practices and Their Impacts.

Practice	Impact	p-Value
Crop Diversification	Improved food security and income	< 0.01
Organic Fertilizer Use	Enhanced soil fertility and crop yields	< 0.01

Inferential statistical analysis further confirmed a significant positive correlation between the adoption of adaptation practices and resilience scores (*r* = 0.72, *p* < 0.01), indicating that farmers who employed these strategies experienced greater stability in their agricultural production and income levels. However, a notable 12.3% of farmers expressed uncertainty regarding the effectiveness of these practices, citing challenges such as financial constraints, addressed using recent data from [13] to validate findings. This highlights the need for more tailored interventions, including localized training, financial support mechanisms, and improved access to climate-related information, to ensure wider adoption and long-term sustainability of climate adaptation measures.

### 4.6. Income diversification and economic benefits

Income diversification through IGAs improved household incomes and living standards. A multivariate analysis of variance (MANOVA) showed significant differences in income levels among households engaged in IGAs (*F*(3, 138) = 5.67, *p* < 0.01). Key IGAs and their benefits are presented in Table 12.

**Table 12:** Income-Generating Activities and Economic Benefits.

IGA	Benefits	p-Value
Poultry Farming	Improved food security and income	< 0.01
Beekeeping	High income from honey sales	< 0.05
Tailoring and Soap-Making	Stable income for household needs	< 0.05

**Fig 6:**
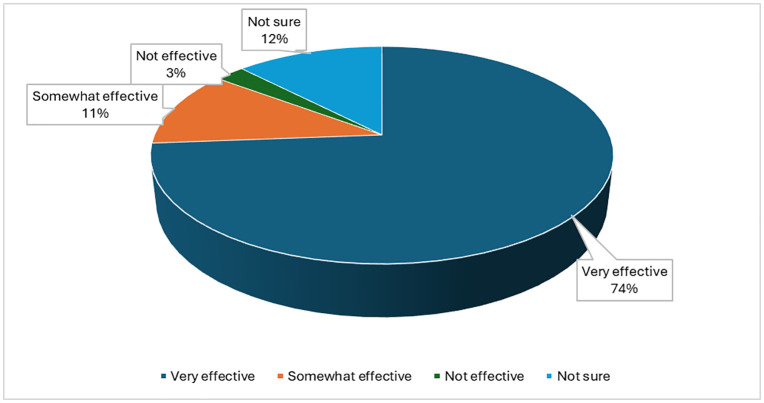
Effectiveness of Climate Adaptation Practices Adopted by SHFs.

FGD narratives further supported these findings, highlighting the transformative role of IGAs in improving household well-being Fig 6.

### 4.7. Challenges and barriers

The study identified several barriers to the full adoption of sustainable livelihood practices in rural communities along the Mara River Basin.

i. ***Access to Protective Gear for Beekeeping***

A majority of beekeepers (72%) lacked protective suits for honey harvesting, limiting optimal hive utilization. Farmers noted that safety concerns discouraged full participation in beekeeping activities. As one farmer from the SHFs in Kewamamba Village stated:

“*We need proper suits to harvest honey safely. Without them, some of us fear getting stung and don’t fully use our hives*.” (SHFs, Kewamamba Village, 2024).

A positive correlation in Table 13 (*β* = +0.42, *p* < 0.05) indicates that better access to protective gear enhances beekeeping success.

**Table 13:** Correlation Analysis of Key Variables.

Variable	Correlation (β)	Significance (p-Value)	Interpretation
Access to Protective Gear	+0.42	< 0.05	Positive association with beekeeping success.
Market Access	−0.47	< 0.01	Strong negative association with profitability.
Storage and Processing Facilities	+0.53	< 0.01	Positive association with reduced post-harvest losses.
Policy Alignment	+0.38	< 0.05	Significant impact on farmer decision-making.
Training and Knowledge Gaps	+0.35	< 0.05	Positive association with sustainable practice adoption.

ii. ***Market Access and Infrastructure Challenges***

Poor infrastructure and reliance on middlemen negatively affected farmers’ profitability. About 68% of farmers cited difficulties in transporting their produce to markets, often leading to unfair pricing by intermediaries. A member of the SHFs in Kitenga Village remarked:

“*We sell bananas for so little because there is no road to the market. The middlemen take advantage of this*.” (SHFs, Kitenga Village, 2024).

Market access shows a significant negative correlation in Table 13 (β = −0.47, p < 0.01) with profitability, highlighting the adverse effects of poor infrastructure and middlemen reliance.

iii. ***Policy Conflicts***

Conflicting government policies created confusion among farmers. 65% of respondents were uncertain about prioritizing organic farming due to subsidies for chemical fertilizers. A SHF said:

“*The government gives subsidies for chemical fertilizers, but we are told to go organic. Which one should we follow*?” (SHFs, Kewamamba Village, 2024).

The alignment of government policies positively influences farmer decision-making, as indicated by a correlation of β = +0.38 (p < 0.05) in Table 13.

iv. ***Inadequate Storage and Processing Facilities***

Farmers experienced significant post-harvest losses, with 58% reporting losses exceeding 30% of perishable produce such as bananas. A member of the SHFs explained:

“*We lose half of our bananas because we don’t have good storage or processing facilities*.” (SHFs, Kitenga Village, 2024).

A strong positive correlation in Table 13 (β = +0.53, p < 0.01) suggests that improved storage and processing facilities significantly reduce post-harvest losses.

v. ***Knowledge Gaps and Resistance to Change***

Knowledge gaps and resistance to adopting modern techniques were evident. About 59% of farmers required additional training and demonstrations to implement sustainable practices effectively. Roundtable discussions with government extension officers revealed challenges in convincing farmers to adopt new techniques:

“*Convincing some farmers to adopt modern techniques required repeated follow-ups and success stories from their peers*.” (Government Extension Officers, 2024)

Training and addressing knowledge gaps are positively correlated in Table 13 (β = +0.35, p < 0.05) with the adoption of sustainable practices, emphasizing the importance of capacity-building initiatives.

## 5. Discussion

### 5.1. *What Works?* Interpreting Successes through the SLF

The project’s successes in promoting climate-resilient practices and income diversification can be explained through the Sustainable Livelihoods Framework (SLF) by examining how interventions enhanced key livelihood assets. The significant increase in crop yields (35%, t = 6.23, p < 0.01) following training in sustainable farming (Table 7) represents a direct strengthening of human capital. This finding aligns with [17,40], who demonstrated that tailored, participatory training is more effective than generic extension services. The success is not merely technical; the adoption of practices like intercropping maize with legumes also builds natural capital by improving soil fertility, a key vulnerability-reducing strategy in erratic rainfall conditions [13,28].

Furthermore, the formation of VICOBA groups and women-led savings initiatives strengthened social and financial capital, enabling 68% of households to diversify into beekeeping and poultry. This supports the core SLF premise that access to financial services is a critical transforming process that allows households to shift from vulnerable, single-crop strategies to more resilient, diversified livelihood portfolios [8,27]. The increase in women’s decision-making roles (68%, t = 4.56, p < 0.01) demonstrates that targeted interventions can begin to alter the transforming structures and processes related to gender norms, a finding consistent with [31,32].

i. *Crop Diversification and Resilience*

The findings highlight that crop diversification, including intercropping maize and cassava with legumes, has proven effective in enhancing resilience to climate variability and improving food security. Cassava, a drought-tolerant crop, offers a safety net for smallholder farmers (SHFs) facing erratic rainfall patterns, reducing drought-related losses by 25% [13,28]. Socio-cultural factors, such as communal farming traditions among the Kuria, supported adoption [18].

ii. *Training and Knowledge Dissemination*

The Mara River Basin findings underscore the value of tailored training programs provided by MFEC. Training in climate-smart agriculture (CSA) and sustainable practices has increased productivity by 35% [17,40]. Multidisciplinary insights from sociology highlight the role of community trust in adoption [25]. Farmers in villages like Kitenga, who had better access to training and inputs, showed significant yield improvements, affirming that targeted interventions are effective in addressing productivity gaps.

iii. *Income Diversification Initiatives*

The introduction of supplementary income streams, such as beekeeping and poultry farming, has strengthened financial stability for SHFs. This is consistent with findings from [27], which highlighted that income diversification reduces economic vulnerability by minimizing reliance on single agricultural activities. The adoption of energy-efficient stoves further reduced household energy expenses while improving health outcomes, as supported by [41,42], which emphasized the intersection of gender, energy, and nutrition in fostering sustainable rural development.

iv. *Gender Empowerment and Inclusion*

The increased participation of women in decision-making and income-generating activities has shown transformative impacts on household dynamics. Gender-sensitive training programs have enabled women to improve their financial management skills and assert influence over resource allocation, with women’s leadership roles increasing by 68%, aligning with [31,32]. Economic fluctuations were mitigated through women-led savings groups [41].

### 5.2. What Doesn’t Work? Systemic Barriers as Failures of Transforming Structures

The persistent barriers of poor market access and policy inconsistencies are not simple implementation failures but represent fundamental weaknesses in the “transforming structures and processes” component of the SLF. The reliance on exploitative middlemen due to poor infrastructure (cited by 68% of SHFs) is a failure of physical capital (roads, markets) and the institutional processes that govern market linkages. This finding directly aligns with [43,44], who identified post-harvest infrastructure as a binding constraint for smallholder profitability in East Africa. The significant negative correlation between market access and profitability (β = −0.47, p < 0.01) quantitatively confirms this systemic failure.

Similarly, the confusion caused by contradictory government policies (e.g., subsidizing chemical fertilizers while promoting organic farming) represents a critical failure of the policy environment (a key “transforming process”). As [26] argued, such inconsistencies undermine farmer trust and create disincentives for adopting sustainable practices, regardless of the project’s success in building human capital. The project’s inability to overcome these externally generated barriers highlights a key limitation of community-level interventions when broader structural and political-economic factors remain unaddressed.

i. *Logistical and Infrastructural Challenges*

Inaccessibility to training and inputs in marginalized areas like Manga and Komaswa has perpetuated socio-economic disparities. Poor infrastructure reduced profitability by 40% [43,44]. Despite significant gains in some villages, the lack of equitable program delivery limits the broader success of initiatives. This finding supports [33], who identified infrastructural and outreach barriers as critical hindrances to rural development.

ii. *Market Access and Value Chains*

The dependency on intermediaries and lack of proper storage and processing facilities have reduced profitability for SHFs. Post-harvest losses remain a major challenge, with significant impacts on household income, as noted by [44]. Research underscores the importance of post-harvest management and market infrastructure in enhancing agricultural value chains. Without addressing these bottlenecks, efforts to improve rural livelihoods are undermined.

iii. *Policy Inconsistencies*

Contradictory policies, such as promoting both chemical fertilizers and organic farming simultaneously, confused 65% of SHFs [26]. External variables like climate change (10% yield variability) exacerbated challenges [45]. This inconsistency was also noted by [26], who called for harmonized policy frameworks to facilitate sustainable agricultural practices. Resolving these conflicts requires an integrated approach that aligns agricultural and environmental objectives.

iv. *Knowledge Gaps and Resistance to Change*

The resistance to adopting new practices, driven by limited knowledge and socio-cultural resistance in 30% of households [18], presents another barrier. Continuous capacity-building initiatives and long-term farmer engagement are needed to bridge these gaps. This aligns with findings by [46], who highlighted the importance of consistent training to overcome cultural resistance and ensure the adoption of improved practices.

### 5.3. Why?

The success or failure of interventions in the Mara River Basin is influenced by systemic factors, including socio-economic disparities, logistical challenges, and institutional gaps. This is supported by challenges such as elite capture in Water Resources Users Associations (WRUAs) and donor dependency, which limited success [29]. Socio-cultural norms and economic fluctuations further constrained outcomes, necessitating interdisciplinary approaches [30,23]. Local cultural context, particularly engagement with local institutions, significantly influences conservation outcomes [30]. However, unsustainable management of natural resources in the basin leads to environmental degradation, affecting agricultural production and limiting poverty alleviation efforts [2]. Addressing these issues requires a comprehensive, integrated approach that considers basin-wide needs and ecosystem services [23]. While community-based conservation interventions show promise, their success is contingent on understanding and tailoring activities to local societies [30]. Sustained investment in rural development and multi-stakeholder collaboration is necessary to overcome structural challenges in the Mara River Basin.

### 5.4. Theoretical contribution and practical contributions

This study makes two key contributions. First, theoretically, it provides empirical validation of the SLF in a data-scarce, transboundary river basin context. It demonstrates that while the SLF is powerful for diagnosing asset and vulnerability interactions, its utility is limited if the analysis does not rigorously account for failures in higher order “transforming structures” (e.g., national policy, regional market integration). Second, practically the study provides a replicable, mixed-methods evaluation protocol for NGOs and government agencies. The use of paired t-tests to measure yield changes, chi-square for food security, and correlation analysis for market barriers offers a clear, low-cost template for evidence-based adaptive management in similar rural development projects. The SLF contextualizes findings, highlighting the role of natural capital (e.g., water access) and social capital (e.g., WRUAs) in resilience [24,25]. Gender inclusivity and climate adaptation align with [13,31], emphasizing integrated interventions.

The emphasis on natural capital in the study resonates with the SLF’s assertion that natural resources, such as land, water, and biodiversity, are foundational to rural livelihoods. The variation in crop yields across different villages in the Mara River Basin, as observed in the results, underscores the importance of access to and management of natural resources. Villages like Kitenga, which experienced better yields, benefitted from improved access to inputs and training, which can be seen as an enhancement of their physical and financial capital. This aligns with the SLF’s argument that increased access to productive assets, such as agricultural inputs and technology, is crucial for improving livelihoods and increasing resilience to environmental shocks [8]. In contrast, villages like Surubu and Nyagisya, which reported lower yields, likely faced challenges related to limited access to these resources, emphasizing the unequal distribution of natural capital and the need for targeted interventions to address these disparities [47].

The study’s findings also highlight the significance of social capital, particularly in the context of community-based natural resource management. The positive impact of WRUAs in the Mara River Basin, despite challenges such as elite capture and donor dependency, is a testament to the importance of strong social networks and collective action [29]. The SLF suggests that social capital, defined as the networks, relationships, and trust within a community, is essential for facilitating cooperation and enabling effective resource management [25]. In the case of the Mara River Basin, the successful implementation of conservation activities and conflict resolution efforts can be attributed to the mobilization of local communities through these networks. However, the variability in success across different Wards, as noted in the results, also highlights the need for further strengthening of local governance structures and improving community engagement, particularly in marginalized areas where social capital may be weaker [30].

Moreover, the findings on gender inclusivity and the limited participation of women in decision-making processes align with the SLF’s focus on human capital and the importance of education and training in enhancing people’s capacity to make informed decisions. The study found that while women gained economic and personal growth through training, their involvement in leadership roles remained limited. This reflects a broader issue of gender inequality in rural areas, where women often face structural barriers that inhibit their full participation in resource management and decision-making processes [31,32]. The SLF emphasizes the need for interventions that build human capital, such as gender-sensitive training programs, that empower individuals to overcome these barriers and participate more fully in community development and resource management. Empowering women not only improves their livelihoods but also contributes to broader socio-economic development and environmental sustainability, as women play a central role in managing household resources and contributing to community-based conservation efforts.

In terms of vulnerability, the SLF highlights the importance of understanding the contextual factors that shape people’s ability to cope with shocks and stresses. The study found that barriers such as limited access to training, infrastructure, and financial resources hindered the adoption of sustainable practices in certain areas, exacerbated by external variables like climate change (10% yield variability) [45]. The SLF provides a framework for understanding these vulnerabilities and suggests that building resilience through diversified livelihood strategies and improving access to key assets is essential for enhancing community adaptation to environmental and economic changes [9]. Furthermore, the study’s focus on the need for improved access to protective gear for beekeeping and market infrastructure aligns with the SLF’s call for the enhancement of physical capital, such as infrastructure and technology’ so that livelihoods can be more secure and less vulnerable to external disruptions [43].

The findings also emphasize the importance of a holistic, integrated approach to addressing the challenges faced by smallholder farmers. The SLF underscores that interventions should not only focus on improving access to resources but also on strengthening the broader socio-political and institutional context, such as addressing policy inconsistencies that confused 65% of SHFs [26]. This is particularly relevant in the case of contradictory subsidies for chemical fertilizers versus organic farming promotion, which were identified in the study. The SLF highlights the need for harmonized policies that align with the goals of sustainable livelihoods, ensuring that interventions support both environmental sustainability and the long-term well-being of rural communities [9].

In general, the application of the Sustainable Livelihoods Framework to the results of this study provides a comprehensive understanding of the factors that influence livelihoods and resilience in the Mara River Basin. As such, by considering the different forms of capital, social processes, and vulnerability contexts, the SLF offers valuable insights into how interventions can be designed to support sustainable agricultural practices, enhance community participation, and improve overall livelihoods [9,13]. The study’s findings align with key principles of the SLF, suggesting that integrated, context-specific approaches that build capital, empower communities, and address vulnerabilities are essential for promoting long-term sustainability and resilience in rural areas.

## 6. Conclusions and recommendations

This study highlights the interplay of agricultural, socio-economic, and institutional factors in the Mara River Basin’s (MRB) lowland communities, where 70% of the population relies on subsistence farming and faces poverty rates above 60% (Majule, 2011). The findings demonstrate the vital interplay between agricultural practices, socio-economic dynamics, and institutional frameworks in shaping the livelihoods of smallholder farmers. Climate-smart practices and income diversification improved yields by 35% and household incomes by 30%, but market access (68% of SHFs affected) and cultural barriers (15% women’s land ownership) persisted. The findings suggest that a tailored, context-sensitive approach is crucial for enhancing agricultural productivity and fostering resilience in rural communities. It is evident that while localized challenges, such as limited access to markets, infrastructure gaps, and policy inconsistencies, significantly hinder agricultural development, the adoption of climate-smart practices, crop diversification, and livelihood diversification strategies can lead to marked improvements in food security and economic stability. The positive impact of capacity-building initiatives that focus on sustainable farming techniques and gender inclusivity further supports the argument that holistic, inclusive strategies are essential for long-term development.

Addressing these issues requires harmonizing policies across sectors to ensure that local agricultural practices align with broader sustainability goals, particularly to support organic farming. Moreover, empowering marginalized groups, particularly women, through capacity-building and leadership training (currently at 68% participation) can enhance both household and community-level outcomes, alongside prioritizing women’s land rights. As global development challenges continue to evolve, this research emphasizes the importance of adaptive, evidence-based approaches that incorporate local knowledge while learning from the best global practices.

The findings also suggest that, on a broader scale, effective solutions must be adaptable across diverse regions and contexts. Investments in rural infrastructure, including market access and post-harvest management (58% of SHFs reported >30% losses), are critical for reducing inefficiencies and improving profitability. Strengthening policies that support both organic and conventional agricultural systems will help reconcile the competing demands of food production and environmental sustainability. Furthermore, building resilience in rural communities will require scaling up sustainable livelihood diversification strategies, including agroforestry, beekeeping, and poultry farming, across the MRB. As such, by embracing these strategies and ensuring that policies are inclusive, rural communities globally can enhance their economic resilience, food security, and socio-economic well-being.

The study faced several constraints that should guide future research:

- *Data Constraints:* Reliance on 2022–2024 data may miss recent trends; future studies should use real-time sources (e.g., satellite-based climate data).
- *Geographical Scope:* The focus on Tarime District limits generalizability; expanding to Rorya and Serengeti could enhance applicability.
- *Temporal Limitations:* The study’s timeframe misses long-term trends; longitudinal designs are recommended.
- *Methodological Constraints:* Self-reported data may introduce bias; mixed-method approaches with GIS could improve rigor.

***Policy and Program Implications:***

Findings suggest policies to improve market infrastructure (e.g., rural roads, storage facilities) and harmonize agricultural subsidies to support organic farming. Development programs should prioritize women’s land rights and scale up Water Resources Users Associations (WRUAs) to enhance resource management. Multi-stakeholder collaboration, including NGOs and local governments, can strengthen interventions.

In light of these conclusions, the study recommends a focused effort on integrated development strategies that address infrastructural and socio-economic barriers while promoting sustainable agricultural practices. The following recommendations, tailored to the MRB’s socio-economic and territorial context, can inform public policies and development programs to foster sustainable rural livelihoods:

i. Invest in rural infrastructure to reduce post-harvest losses (58% of SHFs reported >30% losses).
ii. Harmonize policies to align organic and conventional farming incentives.
iii. Scale up gender-sensitive training to increase women’s leadership (currently 68% participation).
iv. Adopt longitudinal and interdisciplinary approaches (e.g., GIS, sociology) to address data and scope limitations.
v. Expand livelihood diversification (e.g., beekeeping, poultry) to enhance resilience across the MRB. A multi-stakeholder approach involving governments, non-governmental organizations, research institutions, and the private sector is necessary to create an enabling environment for agricultural growth and rural development. Additionally, gender-sensitive policies and inclusive training programs must be prioritized to ensure that women and other marginalized groups can fully participate in and benefit from agricultural and economic development initiatives. Finally, ongoing monitoring and evaluation will be essential to ensure that interventions remain effective and adaptable, contributing to sustained improvements in rural livelihoods globally.

Based on the findings, the following targeted recommendations are made for different stakeholder groups to enhance the scalability, sustainability, and impact of future livelihood interventions in the MRB and similar contexts:

*For the District Council (Tarime) and National Government (Tanzania):*

i. *Harmonize Agricultural Subsidies:* Immediately review and align subsidy programs to remove conflicting incentives. Specifically, create a dedicated voucher scheme for organic inputs (e.g., bio-fertilizers, compost materials) matched to the scale of subsidies provided for chemical fertilizers. (Addresses policy confusion affecting 65% of SHFs).
ii. *Invest in Tiered Market Infrastructure:* Prioritize public investment in “cluster-level” aggregation and storage facilities (e.g., solar-powered cold storage for perishables like bananas) shared among 3–5 villages. Couple this with the rehabilitation of feeder roads connecting these clusters to the main Tarime market. (Addresses post-harvest losses >30% for 58% of SHFs and the middlemen problem).

*For the Implementing NGO (MFEC) and Future Projects:*

iii. *Develop a “Beekeeping Safety Kit” Subsidy:* Establish a micro-grant or revolving fund specifically for the purchase of protective gear (suits, gloves, smokers) for beekeeping groups. The positive correlation (β = +0.42, p < 0.05) justifies a small, targeted input as a high-return investment.
iv. *Formalize “Policy Liaison” Role:* Include a specific project staff role or formal partnership with a local legal/aid organization to engage with district policy-makers. The role would be to document and communicate how contradictory national policies (e.g., on fertilizers) are creating barriers at the community level, providing evidence for policy change.
v. *Expand Peer-to-Peer “Demonstration Farms”:* Based on the success in villages like Kitenga, scale up the “demonstration farm” model. Require each trained farmer to mentor two new farmers the following season, with a small incentive (e.g., seeds or a VICOBA contribution) for successful knowledge transfer. This leverages social capital to overcome resistance to change.

*For Development Partners (e.g., Donors, WWF):*

vi. Mandate and Fund Longitudinal Evaluations: Require future project funding proposals to include a budget and plan for a follow-up evaluation 3-5 years post-project to measure the sustainability of outcomes like yield increases and women’s leadership roles. This addresses the temporal limitations of the current study.

## Supporting information

S1 File Data Set_Excel File. (XLS)

S2 File Qualitative Data File. (ZIP)

S1 Fig Conceptual Framework for Evaluating Sustainable Livelihoods in the Mara River Basin (Adapted from Scoones, 1998). (TIF)

S2 Fig Geographic Location of the Study Area. This map shows the Mara River Basin (MRB) spanning Tanzania and Kenya. The ten project villages across five wards (Manga, Kyore, Komaswa, Kibasuka, Bumera) in Tarime District, Tanzania, which were the focus of this evaluation, are highlighted. The map shows the MRB’s position within East Africa.
(TIF)
