# Supplementary material for: Evaluating Effectiveness of Sustainable Livelihood Development in Rural Communities along Mara River Basin, Tanzania: What Works, What Doesn’t Work, and Why?
Source: PLoS One. 2026 Jun 11;21(6):e0351252. doi: 10.1371/journal.pone.0351252 (PMC13258000; doi:10.1371/journal.pone.0351252)
Supplement: S2 File — (ZIP) [file pone.0351252.s002.zip › Round Table Discussion with Project Implementer.docx]

**ANNEX VI: Round Table Discussion with Project Implementer Checklist**

**Final Evaluation Report for Sustainable Livelihood Development of Rural Communities along Mara River Basin, Tarime District, Tanzania**

**Key Discussion Topics**

**1. Social Problem Addressed**

**Q: Were there any social issues and challenges addressed by MFEC during the project implementation?**

**Expanded Response:**
MFEC tackled various social challenges within the project, with notable progress made in fostering harmonious relationships and promoting gender equality among smallholder farmers (SHFs). For instance, efforts to enhance collaboration between men and women at the family and community levels have improved joint decision-making and participation in development activities. As a result, women are increasingly taking on leadership roles within their groups and communities, contributing to a more inclusive environment.

However, challenges remain, especially regarding resource ownership and equitable decision-making within families. Patriarchal norms persist, with men often unilaterally controlling family assets.

The challenges addressed by MFEC are largely identified through participatory needs assessments, where community members highlight their pressing concerns. Occasionally, issues are raised through village leadership correspondence, which MFEC carefully evaluates to ensure alignment with community priorities. However, reliance on village leaders sometimes risks overlooking broader community needs. MFEC prioritizes community-wide consultations, including participatory tools like **Participatory Assessment of Climate and Disaster Risks (PACDR)**, to ensure inclusivity and accuracy in identifying challenges.

In environmental management, MFEC collaborated with local and national partners, such as the Tanzania Meteorological Agency (TMA), to provide weather information crucial for agricultural decision-making. The integration of traditional and scientific knowledge enhanced farmers' capacity to predict weather patterns effectively. Training sessions emphasized sustainable agriculture practices, such as moisture conservation, seed selection, and pest management, which helped SHFs adapt to changing climatic conditions.

**2. Integration of Environmental Sustainability**

**Q: How were environmental concerns integrated into project activities?**

**Expanded Response:**
MFEC has proactively integrated environmental sustainability into its project framework, aligning its activities with the goals of resilience to climate change and sustainable resource management. Below are the key initiatives that showcase MFEC’s commitment to preserving the environment while enhancing community livelihoods:

1. **Agroforestry and Sustainable Agriculture**
   MFEC promoted agroforestry systems and sustainable farming techniques that prioritize ecological balance and resource conservation. Training sessions introduced farmers to methods such as intercropping with leguminous plants, contour farming to reduce soil erosion, and planting drought-resistant tree species. These practices not only enhance soil fertility and conserve soil moisture but also provide shade and windbreaks, contributing to climate resilience. Participatory demonstrations and hands-on training ensured farmers could replicate these practices independently.
2. **Tree Planting and Nursery Management**
   Recognizing the critical role of trees in combating deforestation and climate change, MFEC supported the establishment of community nurseries stocked with indigenous and drought-tolerant tree species. These nurseries served as a source for reforestation programs and farm boundary plantations. While initial enthusiasm was high, challenges such as sustaining nursery maintenance and selecting appropriate planting sites became apparent. In response, MFEC is piloting decentralized nursery systems, where households take collective responsibility for smaller, localized nurseries. This approach has shown promise in increasing tree survival rates and community ownership of the initiative.
3. **Rainwater Harvesting and Water Management**
   With water scarcity being a growing concern, MFEC emphasized rainwater harvesting techniques to improve water availability for households and agriculture. Primary School were supported with water tanks and trained on constructing simple roof-based water collection systems and underground reservoirs using locally available materials. These reservoirs have proven instrumental in buffering against seasonal water shortages, reducing the burden on natural water bodies, and enabling small-scale irrigation during dry spells.
4. **Energy-Saving Technologies for Sustainable Resource Use**
   MFEC championed the adoption of energy-efficient technologies, particularly improved cookstoves designed to reduce firewood consumption. These stoves not only minimize deforestation but also reduce household exposure to indoor air pollution, improving health outcomes. Women, who are the primary firewood collectors, have particularly benefited from this intervention, as it reduces their workload and frees up time for other activities.
5. **Community Sensitization on Ecosystem Services**
   MFEC conducted awareness campaigns to educate communities on the importance of ecosystem services such as water harvesting, biodiversity, and soil fertility. These sessions emphasized the interconnectedness of human activities and environmental health, fostering a sense of responsibility for protecting natural resources. Communities were encouraged to adopt conservation practices such as protecting riparian zones, controlling bushfires, and reducing waste through composting and recycling.
6. **Participatory Climate Action Planning**
   Through participatory tools like the Participatory Assessment of Climate and Disaster Risk (PACDR), MFEC engaged communities in identifying vulnerabilities and co-developing action plans to address climate risks. These plans prioritized climate-smart agriculture, sustainable grazing practices, and the rehabilitation of degraded lands. The inclusive approach ensured that the interventions were contextually relevant and widely accepted by community members.
7. **Collaborative Research and Knowledge Sharing**
   MFEC partnered with academic and research institutions, including Sokoine University of Agriculture (SUA), to advance agroecology and climate-resilient farming. Jointly organized workshops and farmer field schools facilitated the exchange of knowledge on sustainable farming methods and the ecological benefits of integrating biodiversity into farming systems.

**Broader Impact of Environmental Interventions**

Through these initiatives, MFEC has made substantial progress in fostering a culture of environmental stewardship among communities. The interventions have not only mitigated environmental degradation but also enhanced the resilience of livelihoods to climate variability. By embedding environmental concerns into its projects, MFEC continues to position itself as a leader in sustainable rural development, balancing ecological sustainability with the socio-economic needs of its beneficiaries.

**3. Livelihood Diversification**

**Q: What measures were taken to diversify livelihoods among SHFs?**

### **Expanded Response:** To address the economic vulnerabilities of smallholder farmers (SHFs) and reduce their dependency on unpredictable agricultural yields, MFEC implemented a suite of livelihood diversification strategies. These interventions were designed to create sustainable income streams, enhance food security, and build resilience against environmental and economic shocks. The following activities were among the key measures adopted:

1. **Poultry Farming for Income and Nutrition Enhancement**
   MFEC introduced improved poultry farming techniques to smallholder farmers, focusing on semi-intensive systems. Training sessions covered topics such as constructing cost-effective poultry houses, disease identification and management, and the use of affordable local remedies when modern veterinary services were unavailable. Farmers received improved chicken breeds known for higher productivity in eggs and meat. This initiative not only boosted household incomes but also contributed to better nutrition within the community.
2. **Promotion of Beekeeping as a Sustainable Income Source**
   Recognizing the dual benefits of environmental conservation and income generation, MFEC introduced modern beekeeping practices. Training sessions included hive management, colony health monitoring, and honey extraction techniques. Communities were also taken on exchange visits to successful beekeeping projects to inspire and enhance learning.
3. **Introduction of Fish Farming**
   In collaboration with Tarime District authorities, MFEC supported the introduction of fish farming to diversify livelihoods. Farmers received training on site selection, pond construction, and the care of fingerlings sourced from high-quality suppliers. Practical demonstrations is emphasized feeding regimes and water management to maximize yields.
4. **Livestock Management and Value Addition**
   To improve livestock productivity and marketability, MFEC conducted focused training sessions on poultry keeping, sheep, goat, and cattle husbandry. Topics included disease prevention, vaccination schedules, fattening techniques for market readiness, and feed management using locally available resources. This training helped farmers increase the profitability of their livestock enterprises while reducing losses due to disease and poor management.
5. **Introduction of Handcraft and Small-Scale Enterprises**
   MFEC encouraged SHFs, particularly women tailoring, batique and soap making. Training on basic business skills, including budgeting, marketing, and customer engagement, was provided. These activities offered returns and complemented agricultural income, especially during off-farm seasons.
6. **Savings and Credit Groups to Support IGAs**
   MFEC facilitated the formation of savings and credit groups to enable access to financial resources needed for the initial setup of income-generating activities (IGAs). These groups encouraged a culture of saving and provided small loans to farmers for investing in diversified livelihoods.

**Broader Impact of Livelihood Diversification**

Through these initiatives, MFEC transformed smallholder farming communities by expanding their income sources and enhancing resilience. Diversification not only cushioned households against agricultural risks such as crop failure but also provided them with greater financial stability. Additionally, by integrating sustainable practices into these activities, MFEC ensured that livelihood diversification efforts contributed to environmental conservation and long-term community development.

**4. Agricultural Practices and Training**

**Q: How did the project address agricultural challenges?**

### **Expanded Response:** MFEC implemented targeted and comprehensive strategies to tackle key agricultural challenges faced by smallholder farmers (SHFs), enhancing productivity, reducing losses, and building resilience against climate-related shocks. The following interventions reflect the project’s approach to overcoming these obstacles:

1. **Seed Selection and Management**
   Recognizing the importance of resilient seed varieties in coping with unpredictable weather patterns, MFEC prioritized training on selecting drought-resistant and native seeds. These seeds were chosen for their ability to thrive in local conditions while maintaining soil health. To preserve genetic diversity and promote sustainable farming. Farmers were trained in seed collection, storage, and redistribution to ensure the longevity of traditional seed varieties. However, challenges such as inadequate management skills and lack of infrastructure for the seedbanks persist, requiring continued support and refinement.
2. **Post-Harvest Handling and Storage Solutions**
   A significant portion of crop yields is often lost due to poor post-harvest practices. MFEC addressed this issue by training SHFs on proper drying, cleaning, and safe storage techniques. Farmers learned how to use affordable, locally available materials to build temporary silos and airtight storage bags to reduce pest and moisture damage. Farmers were supported with advanced storage facilities, such as hermetic bags and cold storage units. MFEC continues to advocate for partnerships and funding to bridge this gap.
3. **Promotion of Climate-Smart Agriculture (CSA)**
   To enhance resilience against climate change, MFEC introduced SHFs to CSA practices. These included:
   - **Early Land Preparation**: Training on soil preparation techniques such as minimum tillage to retain soil structure and fertility.
   - **Efficient Irrigation**: Farmers were guided in setting up drip irrigation systems and using water-efficient methods like mulching to reduce evaporation.
   - **Integrated Pest Management (IPM)**: SHFs were trained to combine biological, cultural, and chemical methods for pest control to minimize environmental impacts and reduce reliance on costly pesticides.
     Partnerships with the Tanzania Meteorological Authority (TMA) enabled timely dissemination of localized weather forecasts, helping farmers plan planting and harvesting schedules more effectively.
4. **Vegetable Production for Nutrition and Income**
   MFEC expanded livelihood options and addressed nutritional deficits by introducing vegetable farming and home gardening practices. Farmers were taught how to cultivate fast-growing vegetables such as spinach, tomatoes, and amaranth. These crops provided a reliable source of vitamins for households and additional income through market sales. Training also included organic farming methods, which improved soil health and produced chemical-free vegetables, appealing to health-conscious consumers.
5. **Soil Fertility and Conservation**
   MFEC emphasized soil conservation techniques to mitigate the degradation caused by overuse and climate change. Farmers were trained in contour farming, crop rotation, and organic manure application. Additionally, demonstrations on compost-making and biofertilizer production empowered SHFs to enhance soil fertility without relying heavily on synthetic fertilizers.
6. **Strengthening Farmer Organizations**
   MFEC encouraged SHFs to join in farmers group which provided a platform for knowledge-sharing and collective action. Through these groups, farmer shared experiences on overcoming agricultural challenges.

### **Broader Impacts and Lessons Learned**

Through these initiatives, MFEC not only addressed immediate agricultural challenges but also laid the groundwork for long-term sustainability and resilience. While the project achieved notable successes, challenges such as the high cost of advanced storage solutions and limited irrigation infrastructure remain. Addressing these barriers requires increased investment, policy support, and continued collaboration with local and international stakeholders.

By integrating technical training, innovative practices, and community-driven solutions, MFEC contributed significantly to enhancing agricultural productivity, reducing losses, and ensuring food security for SHFs.

**5. Community Engagement and Participation**

**Q: How were communities engaged to ensure participation and sustainability?**

### **Expanded Response:** MFEC placed a strong emphasis on community engagement, employing inclusive and participatory methods to ensure ownership, active participation, and sustainability of project activities. The following narratives illustrate the approach and its outcomes:

1. **Participatory Development Approach**
   Central to MFEC’s strategy was the **Participatory Assessment of Climate and Disaster Risks (PACDR)** tool. Through this approach, community members actively engaged in identifying the challenges they faced, mapping available resources, and devising strategies for sustainable utilization. This process empowered participants to define their priorities, increasing their commitment to the solutions. For example, communities identified degraded grazing lands as a key issue and collaboratively implemented rotational grazing systems to restore them.
2. **Inclusive Village-Wide Meetings**
   To ensure no one was left behind, MFEC organized inclusive village-wide meetings. These gatherings served as platforms for exchanging ideas, voicing concerns, and aligning community needs with project objectives. Diverse groups, including women, youth, elders, and persons with disabilities, were explicitly invited to participate.
3. **Formation and Strengthening of SHF Groups**
   MFEC encouraged the formation of **smallholder farmer (SHF) groups** to foster collaboration and collective action. These groups provided a platform for farmers to pool resources, share knowledge, and access training more effectively.
   - **Gender-Balanced Representation**: Special attention was given to gender balance in forming and managing these groups. Women were encouraged to take leadership roles, ensuring equitable participation and fostering diversity in decision-making processes.
4. **Community Training and Knowledge Sharing**
   MFEC’s training sessions were interactive and practical, focusing on building the skills of community members. Demonstration farms, farmer field schools, and exchange visits enabled peer-to-peer learning, creating a ripple effect of knowledge dissemination. For instance, SHFs trained in beekeeping shared their expertise with neighboring villages, extending the project’s reach beyond its initial scope.
5. **Feedback and Adaptive Management**
   Regular feedback sessions allowed communities to share their experiences and challenges, ensuring that the project remained responsive to their evolving needs. These sessions highlighted the importance of ongoing capacity building, particularly in areas like resource management, climate adaptation, and group governance. The iterative process of receiving feedback and adapting interventions helped refine project activities and enhance their effectiveness.
6. **Youth and Women Empowerment**
   MFEC recognized the transformative potential of engaging youth and women in development activities. Tailored programs provided them with technical skills in areas such as agro-processing, market analysis, and leadership. Youth-focused initiatives, such as -based climate information systems.
7. **Cultural Sensitivity and Social Inclusion**
   MFEC incorporated cultural values and norms into its engagement strategies, ensuring that interventions resonated with local traditions. For example, community elders were involved as deliberate efforts were made to challenge harmful practices, such as exclusionary gender roles, through advocacy and education campaigns.
8. **Sustainability Through Local Institutions**
   To embed sustainability, MFEC partnered with local institutions such as village councils, women’s associations and youth groups. These entities were trained to take over project components, such as managing farmers, ensuring the continuity of activities even after project completion.

### Impact and Lessons Learned

The participatory and inclusive approaches adopted by MFEC fostered a sense of ownership and accountability among community members. By involving diverse stakeholders, the project gained widespread acceptance and achieved significant progress in addressing social, environmental, and economic challenges.
However, lessons learned from the engagement processes underscored the need for:

- **Strengthened group governance**: Addressing challenges in leadership and decision-making within SHF groups.
- **Enhanced resource mobilization**: Ensuring communities have access to adequate resources for scaling up successful initiatives.
- **Continuous technical support**: Providing ongoing capacity-building programs to adapt to emerging challenges.

By fostering collaboration, inclusivity, and shared responsibility, MFEC laid a strong foundation for the long-term sustainability of its interventions. Further partnerships and investments will be vital to building on this success and scaling the project’s impact

Top of Form

Bottom of Form

***Other initiatives by MFEC......***

**Resolution of Land Conflicts through the 'No Man’s Land' Initiative**
MFEC has successfully implemented the *No Man’s Land* initiative, which aims to mitigate land conflicts within the communities. This approach designates disputed land as a neutral area that is either conserved, utilized for communal purposes, or distributed equitably based on stakeholder agreements. By promoting dialogue and inclusive decision-making, this initiative has contributed significantly to reducing tensions and fostering harmonious coexistence among different groups.

**Staff Capacity Building with a Focus on Monitoring and Evaluation (M&E)**
Recognizing the importance of effective project management and accountability, MFEC has invested in the professional growth of its staff, particularly in the area of Monitoring and Evaluation (M&E). This has involved tailored training sessions, workshops, and mentorship programs to enhance skills in data collection, analysis, and reporting. These efforts ensure that MFEC projects are not only impactful but also align with donor expectations and community needs.

**Collaborative Efforts with Partner Organizations**
MFEC has strengthened its partnerships with key stakeholders such as **PELUM Tanzania**, **Sustainable Agriculture Tanzania (SAT)**, **Ukiliguru Agricultural Research Institute**, and **MVIWANYA**. These collaborations have facilitated the sharing of best practices, co-learning, and co-hosting of events such as COP (Community of Practice) meetings. In these forums, stakeholders deliberate on lessons learned, identify challenges, and strategize for improved implementation of projects. Topics discussed include issues such as Female Genital Mutilation (FGM) with Alliance for Termination of Female Genital Mutilation (ATFGM MASANGA), Child Dignity Forum (CDF), CARE FOR AFRICA on school feeding, Climate Action Network (CAN) on use of climate and weather information and the promotion of agroecology principles as sustainable farming alternatives.

**Establishment of an Agroecology Hub in Collaboration with SUA**
MFEC, in partnership with Sokoine University of Agriculture (SUA), is spearheading the establishment of an Agroecology Hub. This hub aims to provide comprehensive training for farmers on sustainable farming practices. The initiative focuses on soil health, integrated pest management, crop diversification, and climate-resilient farming techniques, enabling farmers to adapt to environmental challenges while boosting productivity.

**Acknowledgment of Support from Bread for the World**
MFEC extends its gratitude to **Bread for the World**, which has played a pivotal role in funding and supporting community development initiatives. Through participatory approaches like the **Participatory Organizational Planning (POP)** framework and the **Participatory Assessment of Climate and Disaster Risk (PACDR)** methodology, MFEC has been able to engage communities in planning and executing activities that enhance their resilience to climate change and socio-economic challenges.
