# Supplementary material for: Evaluating Effectiveness of Sustainable Livelihood Development in Rural Communities along Mara River Basin, Tanzania: What Works, What Doesn’t Work, and Why?
Source: PLoS One. 2026 Jun 11;21(6):e0351252. doi: 10.1371/journal.pone.0351252 (PMC13258000; doi:10.1371/journal.pone.0351252)
Supplement: S2 File — (ZIP) [file pone.0351252.s002.zip › School Feeding Programme - Nyagisya Village.docx]

**ANNEX V: School Feeding Programme Committee Focus Group Discussion (FGD) at Nyagisya Primary School**
**Final Evaluation: Sustainable Livelihood Development of Rural Communities along Mara River Basin, Tarime District, Tanzania**
**Discussion Guide: Kyore Ward, Nyagisya Village**

### **1. The Project’s Role in Schools**

**Q: Can you briefly describe the contribution of MFEC to your school?**

**Responses:**
Mogabiri Farm Extension Centre (MFEC) is recognized as a community service organization operating under the Anglican Church in Tarime District. Its contributions to our school have been diverse and impactful, including:

- **Agricultural Support:** MFEC introduced modern agricultural practices, such as improved maize and potato seeds, enabling the school to establish sustainable farming activities. This has significantly boosted food production and supported the school feeding program.
- **Infrastructure and Equipment:** The organization provided essential tools, utensils, and energy-efficient stoves to facilitate meal preparation. Plates, cups, and cooking pots were distributed to ensure every child had access to hygienic and equitable feeding conditions.
- **Nutrition and Health:** MFEC worked closely with parents and the school community to establish a feeding program. Parents were encouraged to contribute maize (two kilograms per child 3months), and the harvested crops supplemented the program. The project also included deworming and schistosomiasis vaccinations for students.
- **Gender Sensitization:** Through students’ clubs, MFEC raised awareness about harmful cultural practices like female genital mutilation (FGM) and early marriages, as well as the importance of gender equality. The program included gender-responsive teaching and supported the establishment of a gender desk at the school.
- **Environmental Conservation:** MFEC donated 500 tree seedlings to promote environmental sustainability.
- **Empowering Women:** The project improved the economic capacities of women, reducing dependency and enhancing their roles in household decision-making.

**Outcomes:**

- Increased school attendance and reduced truancy due to improved nutrition.
- Enhanced academic performance as students are healthier and more focused.
- Cultural shifts in the community as harmful practices are addressed and discouraged boys circumcision.

### **2. Impact of School Feeding Programs**

**Q: What does MFEC contribute to the health and performance of students in your school?**

**Responses:**

- **Improved Nutrition:** Students now receive regular, nutritious meals, reducing malnutrition and improving their physical and cognitive health.
- **Academic Excellence:** Healthy students exhibit better concentration, participation, and performance in class. The truancy rate has dropped significantly, with fewer cases of absenteeism due to hunger or illness.
- **Community Empowerment:** MFEC has fostered a sense of collective responsibility among parents, staff, and community members, ensuring the program's sustainability.

**Q: What challenges do you face in implementing the school feeding program, and how do you overcome them?**

**Challenges:**

1. **Parental Engagement:** Some parents were initially reluctant to contribute to the program, citing financial constraints.
2. **Energy for Cooking:** Firewood collection by students is unsustainable and labor-intensive.
3. **Food Preservation:** Lack of proper storage facilities often leads to food spoilage.

**Solutions:**

- Conducting regular awareness sessions to educate parents on the program's importance.
- Seeking support for energy-efficient stoves to reduce dependence on firewood.
- Partnering with MFEC and other stakeholders to explore options for constructing a food storage facility.

### **3. Involvement Process**

**Q: Did you in any way involve parents in the school feeding program?**

**Responses:**
Yes, parents are actively involved through various initiatives:

- Contributions of maize (two kilograms per student per season).
- Participation in decision-making and oversight through the school committee.
- Attendance at quarterly meetings to discuss challenges, finances, and program updates.

**Q: Did you face any difficulties in the process?**

**Responses:**
Yes, initial resistance from some parents due to financial difficulties and lack of understanding about the program's benefits. However, this was mitigated through continuous engagement and showcasing the program’s positive impact on students’ health and education.

**Q: Are there other stakeholders involved in the school feeding program?**

**Responses:**
Yes, stakeholders include the government, community leaders, and development partners such as MFEC, who provide technical and material support.

### **4. Sustainability of School Feeding Programs**

**Q: To what extent is the school feeding program sustainable in the absence of MFEC?**

**Responses:**
The program has shown significant sustainability due to the structures and practices established by MFEC. Key indicators of sustainability include:

- Strong parental involvement in food production and contributions.
- Effective management by the school committee.
- Community awareness and ownership of the program.
- Continued partnerships with local organizations and government support.

**Q: What are the indicators to ensure the sustainability of the school feeding program?**

**Indicators:**

- Transparent reporting systems for accountability.
- Regular capacity-building sessions for the school committee and parents.
- Community-wide commitment to gender equity and shared responsibility in program activities.

### **5. Lessons Learned and Recommendations**

**Q: Based on your experience with the project, what were the key lessons learned related to improving school feeding programs?**

**Lessons Learned:**

- Gender-focused education is crucial for addressing systemic inequalities and improving family dynamics.
- Consistent parental engagement ensures program sustainability.
- Addressing cultural barriers requires patience, continuous education, and community dialogue.
- Women, when economically empowered, play a pivotal role in managing household resources efficiently.

**Q: What would you recommend for future projects in improving school feeding programs?**

**Recommendations:**

1. Expand gender education to align with cultural norms while promoting equality.
2. Introduce financial literacy training for parents to enhance their economic independence and reduce gender-based violence.
3. Invest in sustainable energy solutions, such as energy-saving stoves, to minimize reliance on firewood.
4. Construct storage facilities for food preservation.
5. Strengthen collaboration with stakeholders to ensure continuous funding and technical support.

This focus group discussion highlighted the transformative role of MFEC in promoting education, health, and gender equity in Nyagisya Primary School, offering valuable insights for scaling similar initiatives.

Top of Form

Bottom of Form
