# Supplementary material for: Evaluating Effectiveness of Sustainable Livelihood Development in Rural Communities along Mara River Basin, Tanzania: What Works, What Doesn’t Work, and Why?
Source: PLoS One. 2026 Jun 11;21(6):e0351252. doi: 10.1371/journal.pone.0351252 (PMC13258000; doi:10.1371/journal.pone.0351252)
Supplement: S2 File — (ZIP) [file pone.0351252.s002.zip › School Feeding Programme - Turugeti Village.docx]

**Final Evaluation: Sustainable Livelihood Development of Rural Communities along Mara River Basin, Tarime District, Tanzania**

**ANNEX V: School Feeding Programme Committee Focus Group Discussion (FGD)**
**Discussion Guide**

**1. The Project’s Role in Schools**

**Can you briefly describe the contribution of MFEC to your school?**
The Mogabiri Farm Extension Centre (MFEC) has significantly contributed to the development of our school, Turugeti Primary School. Through its initiatives, the following achievements were realized:

- **School Feeding Program**: MFEC introduced and supported agricultural projects that directly benefited the school feeding program. This included the provision of farming inputs, training on sustainable agricultural practices (e.g., organic farming to preserve soil fertility), and the distribution of utensils such as plates, drinking cups, and cooking pots. Additionally, MFEC provided a water tank for rainwater harvesting, ensuring reliable access to clean water.
- **Improved Nutrition and Academic Performance**: The introduction of better-quality seeds and sustainable farming methods has ensured the production of nutritious crops, which are now used in the school feeding program. Consequently, students now have access to a consistent supply of nutritious meals, resulting in improved health and focus in class. Academic performance has also improved, with fewer cases of truancy.
- **Gender and Community Awareness**: The project included education on gender issues, such as advocating against harmful practices like female genital mutilation (FGM) and promoting the education of girls. MFEC raised awareness among students and parents about gender equality and the importance of involving women and men in decision-making processes.

**Challenges Encountered**:
Some parents were initially resistant to contributing to the program, citing financial constraints or a lack of awareness about its importance. Over time, as they witnessed the positive changes in their children’s academic performance and health, many began to support the program by donating maize and beans.

**Role of the School Feeding Committee**:
The committee oversees food supply and utilization, ensuring transparency. It also encourages contributions from parents, coordinates with teachers for program implementation, and regularly reports to parents and stakeholders.

**2. Impact of School Feeding Programs**

**What does MFEC contribute to the health and performance of students in your school?**
The project has significantly impacted students’ well-being and academic performance. Key highlights include:

- **Health Improvements**: Regular meals have reduced cases of malnutrition among students. Improved health has resulted in better class attendance and participation in co-curricular activities like sports and cultural events.
- **Enhanced Academic Performance**: With access to consistent meals, students are more attentive and productive during lessons. Truancy and dropout rates have decreased significantly, as students now look forward to attending school.
- **Capacity Building**: MFEC conducted training for parents, school staff, and committee members on food safety, hygiene, and efficient management of the feeding program. This training emphasized community ownership and involvement, fostering a sense of shared responsibility.

**Challenges in Implementation and Overcoming Them**:

- **Parental Resistance**: Some parents initially hesitated to contribute food items or funds due to financial constraints. To address this, the committee organized community awareness meetings, emphasizing the long-term benefits of the program.
- **Resource Constraints**: Limited access to cooking facilities, water, and funds for hiring cooks posed challenges. These were addressed through in-kind contributions from parents and the support of MFEC in providing essential materials.

**3. Involvement Process**

**Did you in any way involve parents in the school feeding program?**
Yes, parents were actively involved from the outset. They contributed maize, beans, and occasionally money to support the program. Regular meetings were held to update them on the program’s progress, fostering transparency and trust.

**Did you face any difficulties in the process?**
Some parents expressed reluctance to contribute due to financial hardships. However, continuous sensitization campaigns highlighting the benefits for their children gradually improved participation.

**Are there other stakeholders involved in the school feeding program? Who are they?**
Yes, apart from MFEC, the village government played a crucial role in mobilizing community support. Local leaders helped organize meetings, while teachers ensured the smooth implementation of the program within the school.

**4. Sustainability of School Feeding Programs**

**To what extent is the school feeding program sustainable in the absence of MFEC?**
The sustainability of the program relies heavily on community involvement and government support. While the community has shown willingness to contribute, additional support is required to maintain momentum. Specifically:

- **Government Support**: Assistance is needed to support orphaned and vulnerable children who lack family support.
- **Infrastructure Development**: There is a need for additional resources, such as water wells and improved cooking facilities.

**Indicators of Sustainability**:

- Active participation of parents and the school committee in food production and management.
- Establishment of transparent reporting systems to maintain trust and accountability.
- Regular training sessions to build capacity among new committee members and ensure knowledge transfer.

**5. Lessons Learned and Recommendations**

**Key Lessons Learned**:

- **Community Engagement is Key**: Involving parents and the community in every stage of the program fosters ownership and ensures long-term success.
- **Holistic Approach Matters**: Addressing gender issues, health, and academic performance together yields better outcomes for students.
- **Transparency Builds Trust**: Open communication and transparent management encourage continued community support.

**Recommendations for Future Projects**:

- **Water Infrastructure**: Future projects should prioritize access to clean water by constructing wells or boreholes within schools.
- **Sustained Capacity Building**: Regular training sessions for parents, teachers, and committee members should continue to ensure the program’s sustainability.
- **Government Partnership**: The government should take an active role in supporting school feeding programs, particularly for vulnerable students.
- **Support for Local Agriculture**: Encourage the use of organic fertilizers and sustainable farming practices to enhance food security.

Therefore, this evaluation underscores the transformative role of MFEC in promoting education, health, and community development in Tarime District. Through enhanced collaboration and sustained efforts, the program can serve as a model for other rural communities in Tanzania.
