# Supplementary material for: Evaluating Effectiveness of Sustainable Livelihood Development in Rural Communities along Mara River Basin, Tanzania: What Works, What Doesn’t Work, and Why?
Source: PLoS One. 2026 Jun 11;21(6):e0351252. doi: 10.1371/journal.pone.0351252 (PMC13258000; doi:10.1371/journal.pone.0351252)
Supplement: S2 File — (ZIP) [file pone.0351252.s002.zip › Extension Officers-Round table discussion.docx]

**Annex IV: Round Table Discussion with Extension Officers**

**Overview of Project Activities and Outputs**

**Question:** *Can you provide an overview of the extension services you provided to the farmers under the MFEC project?*

Extension officers highlighted their collaborative efforts with the Mogabiri Farm Extension Centre (MFEC) to deliver an array of extension services aimed at improving sustainable livelihoods for rural communities in the Mara River Basin, particularly in Tarime District.

In **Bumera Ward**, the project targeted farmer groups across three villages to address climate resilience through customized training programs. These programs emphasized climate-smart agricultural practices such as conservation tillage, agroforestry, and sustainable water use. Farmers not only received theoretical knowledge but also engaged in practical demonstrations, making the training impactful and actionable.

The officers also facilitated **school feeding programs**, providing improved seed varieties and overseeing their equitable distribution. They conducted training sessions on modern agricultural techniques, post-harvest management, and value addition to help farmers minimize losses and boost income. Efforts were complemented by the introduction of **Income-Generating Activities (IGAs)**, such as soap making, poultry farming, and small-scale enterprises, diversifying household income sources.

The project addressed critical agricultural challenges, including **cassava mosaic disease** and **banana streak virus**, through demonstration farms showcasing improved farming techniques. Other initiatives included:

- Promoting **sustainable livestock management** practices.
- Encouraging **afforestation** by distributing fruit tree seedlings.
- Implementing **soil erosion control** measures like water conservation structures.

Beyond agricultural interventions, MFEC incorporated **social development programs** to tackle pressing community issues, such as gender inequity and harmful cultural practices like **female genital mutilation (FGM)**. The integration of these social aspects strengthened community cohesion and awareness.

**Farmer Adoption of Services**

**Question:** *How did the farmers perceive and adopt the training and services you provided?*

Farmers exhibited a strong willingness to embrace the extension services. Adoption rates exceeded **50%**, with significant uptake in the use of improved seeds, modern farming techniques, and sustainable practices. Success stories—such as increased crop yields and household incomes—encouraged peer learning and wider adoption within the communities.

The establishment and expansion of **Village Community Banks (VICOBAs)** proved transformative. Supported by financial literacy and entrepreneurship training, these community-based savings and loan groups empowered farmers to finance their ventures independently, ensuring long-term sustainability.

**Effectiveness of Implementation Strategies**

**Question:** *What strategies did you use to provide extension services to the farmers?*

The extension services employed participatory and hands-on approaches to address farmers' specific needs. The main strategies included:

- **Practical demonstrations:** Farmers actively engaged in on-farm trials and demonstrations.
- **Group engagements:** Peer-to-peer learning through farmer groups.
- **Model farms:** Real-world examples of best practices to encourage adoption.

**Challenges Encountered**

**Question:** *Which challenges did you encounter in providing extension services under MFEC?*

Despite significant progress, several challenges emerged:

1. **High input costs:** Limited affordability of improved seeds and inputs.
2. **Inconsistent participation:** Irregular attendance at training sessions by some farmers.
3. **Resistance to change:** A minority of farmers resisted adopting new practices, often requiring extended follow-ups and demonstrations of tangible benefits to overcome this hesitation.

**Gender Responsiveness**

**Question:** *Is there any approach or tool adopted from MFEC to assist in extension service delivery to farmers?*

The MFEC approach prioritized **gender inclusivity**:

- **Farmer group composition:** Balanced participation of men and women.
- **Leadership opportunities:** Women were encouraged to assume leadership roles, particularly in financial management.
- **Dedicated support:** Women-only groups received additional training and access to credit facilities.
- **Women’s Empowerment Desks:** These platforms facilitated women’s involvement in decision-making and community development initiatives.

**Impact**

**Question:** *What was it and how did it work?*

The project significantly empowered women, enhancing their economic contributions and leadership within their communities. While cultural barriers, such as men’s reluctance to engage in mixed-gender groups, persist, there has been a noticeable shift toward gender equity in decision-making processes.

**Monitoring, Evaluation, and Tracking Progress**

**Question:** *Was the tool useful in ensuring gender responsiveness when delivering extension services?*

The implementation of extension services was closely monitored through:

- **Regular farmer group meetings:** Provided opportunities for feedback and adjustment.
- **Field visits and assessments:** Enabled real-time progress tracking.
- **Collaboration with local authorities:** Strengthened oversight and reduced implementation gaps.

**Climate Change Adaptation and Success Stories**

Extension services significantly enhanced farmers' resilience to climate change by promoting:

- **Climate-smart techniques:** Adoption of conservation tillage, agroforestry, and water conservation structures.
- **Improved crop varieties:** Farmers embraced high-yield, disease-resistant cassava and banana cultivars.
- **Livestock management innovations:** Poultry management practices, particularly among women’s groups, increased household income.

Farmers shared success stories of increased productivity, reduced post-harvest losses, and improved household nutrition, which motivated others to replicate these practices.

**Project Sustainability and Recommendations**

The project’s sustainability is evident through:

- Replication of farmer group models.
- Alignment with government initiatives like VICOBAs and school feeding programs.

**Recommendations:**

1. Increase the number of trained extension officers for broader outreach.
2. Expand MFEC services across all 26 wards in Tarime District.
3. Strengthen MFEC-government collaborations to secure additional funding and technical support.

**Lessons Learned**

Key lessons for future projects include:

1. **Community collaboration:** Engaging grassroots stakeholders ensures greater buy-in and effectiveness.
2. **Demonstration farms:** Practical learning platforms are critical for driving adoption.
3. **Tailored interventions:** Addressing cultural practices and specific community needs fosters long-term acceptance and impact.

The Mogabiri project demonstrates how integrated approaches addressing agricultural, environmental, and social challenges can achieve **sustainable rural development** in Tanzania.
