# Supplementary material for: Evaluating Effectiveness of Sustainable Livelihood Development in Rural Communities along Mara River Basin, Tanzania: What Works, What Doesn’t Work, and Why?
Source: PLoS One. 2026 Jun 11;21(6):e0351252. doi: 10.1371/journal.pone.0351252 (PMC13258000; doi:10.1371/journal.pone.0351252)
Supplement: S2 File — (ZIP) [file pone.0351252.s002.zip › FGDs Kewamamba Village, Kiore Ward.docx]

**ANNEX III: Farmers’ FGDs Checklist**

**Project Final Evaluation on “*Sustainable Livelihood Development of Rural Communities along the Mara River Basin, Tarime District, Tanzania*”**

**Key Discussion Topics in Kewamamba Village, Kiore Ward** **for SHFs Groups:** (i) *WAKULIMA NA WAFUGAJI* Kewamamba Group (ii) FAMOs Group

**1. Project Relevance and Awareness**

**Discussion Prompt:**
*What challenges were addressed by MFEC in farming activities?*

**Expanded Response:**
MFEC has been instrumental in addressing several critical challenges faced by smallholder farmers (SHFs) and livestock keepers in Kewamamba Village. The center introduced training programs aimed at improving agricultural and livestock practices, which were previously inefficient and unsustainable. Farmers mentioned that before MFEC's intervention, challenges such as soil degradation, low crop yields, and lack of proper farming techniques were rampant. MFEC introduced sustainable farming techniques, including organic fertilizer use, crop rotation, and proper spacing during planting. They also provided native, drought-resistant seeds suited to the local environment.

Additionally, MFEC addressed nutritional deficiencies within households by teaching farmers about balanced diets, food security practices, and proper food storage methods. A key highlight was the introduction of energy-efficient stoves and tree-planting initiatives to combat deforestation and soil erosion, which had been major environmental challenges.

The project also tackled social issues like gender-based violence (GBV) by encouraging community dialogue on the subject and promoting women's participation in economic activities. Many women reported gaining knowledge on managing household resources and participating more actively in decision-making within their families and groups.

**Prompt:**
*Was climate change among the challenges addressed by MFEC?*

**Expanded Response:**
Climate change was also among the key challenge addressed by MFEC. Farmers reported training sessions focused on climate adaptation practices such as water harvesting, conservation tillage, and reforestation. They learned techniques like mulching and the integration of agroforestry into their farming systems. These practices have improved their ability to cope with erratic rainfall patterns and prolonged dry seasons. Farmers also adopted mixed farming and diversification into climate-resilient activities such as beekeeping and poultry keeping.

**Effectiveness of the Climate Adaptation Practices:**
Farmers reported that these practices had been highly effective. For example, tree planting has reduced soil erosion and created microclimates that favor better crop growth. Use of organic fertilizers has improved soil fertility, resulting in higher yields. Demonstration farms established by MFEC provided practical examples of how to implement these techniques effectively.

**2. Participation and Engagement**

**Prompt:**
*Was the training provided by MFEC inclusive of both men and women?*

**Expanded Response:**
The training was highly inclusive, involving both men and women equally. Women, in particular, benefited significantly from the training, as it enhanced their participation in income-generating activities and household decision-making. Participants noted that women's involvement improved family well-being because women used the knowledge gained to invest in better nutrition, education, and shelter for their families.

However, some challenges were identified, such as occasional conflicts in households when women’s empowerment was perceived to challenge traditional gender roles. Addressing this issue, MFEC emphasized family-level collaboration and the principle of shared resources and responsibilities, which helped to foster harmony.

**Prompt:**
*Did women’s involvement contribute to skills and decision-making in the community?*

**Expanded Response:**
Women’s involvement greatly enhanced their decision-making skills within both their households and the wider community. Women now play active roles in their farmer groups and community meetings, influencing decisions on farming methods, resource allocation, and income usage. This shift has also led to greater acceptance of women in leadership roles within the village.

**3. Effectiveness and Practical Impact**

**Prompt:**
*What farming practices introduced by MFEC were adopted, and how effective were they?*

**Expanded Response:**
Farmers adopted several MFEC-recommended practices, including:

- **Crop diversification**: Planting a variety of crops like maize, groundnuts, and cassava, which improved household food security and reduced the risks associated with mono-cropping.
- **Organic fertilizer use**: Farmers switched to composting and using manure instead of chemical fertilizers, which enhanced soil health and increased productivity.
- **Bee farming**: This proved to be a lucrative venture, with farmers reporting significant income from honey sales.
- **Improved livestock rearing**: Training on poultry and goat farming improved animal health and yields.

These practices were widely regarded as effective. Farmers noted improved crop yields, healthier soils, and higher incomes as direct outcomes.

**4. Income Diversification and Economic Benefits**

**Prompt:**
*What other income-generating activities (IGAs) are you engaged in, and what benefits have they brought?*

**Expanded Response:**
Farmers diversified their incomes by engaging in IGAs such as:

- Poultry farming (local chicken breeds)
- Beekeeping with over 120 beehives producing honey for local and external markets especially in Kenya
- Fish farming in two constructed ponds
- Trading in livestock (buying, fattening, and selling cattle)
- Selling bananas, groundnuts, and vegetables at local markets

These activities have brought significant benefits, including:

- Increased household income, which has been used to build better houses with iron sheets and send children to school.
- Creation of savings groups within farmer cooperatives, enabling group members to access loans for expanding their ventures.
- Improved food security at the household level, as the IGAs supplement farm production.

**5. Challenges and Barriers**

**Prompt:**
*What challenges have you faced in adopting new farming practices?*

**Expanded Response:**
Some challenges include:

1. **Lack of Protective Gear for Beekeeping:** Farmers noted a significant barrier in the form of inadequate equipment, particularly protective gear required for safe and efficient honey harvesting. This limitation discourages active engagement in beekeeping activities.
2. **Market Access:** Poor infrastructure and over-reliance on exploitative middlemen create difficulties for farmers in accessing markets. This results in selling produce at unfair prices, reducing their earnings and affecting overall sustainability.
3. **Government Policy Conflicts:** While the Mogabiri Farm Extension Centre (MFEC) actively promotes the use of organic fertilizers for sustainable farming, farmers highlighted a conflicting message from government subsidies encouraging the use of chemical fertilizers. This creates confusion and affects the adoption of environmentally friendly practices.
4. **Post-Harvest Losses:** Farmers face considerable post-harvest losses due to inadequate storage facilities, particularly for perishable crops like bananas and avocadoes. These losses undermine their efforts and result in reduced profitability.

**Proposed Interventions by Farmers**

Farmers suggested practical solutions to mitigate these challenges. These include:

- Emphasizing **value addition** activities, such as processing cassava into flour or peanuts into peanut butter, to increase product value and shelf life.
- Facilitating **better access to markets** through improved infrastructure and direct farmer-buyer linkages to eliminate middlemen.

These interventions aim to address both immediate challenges and long-term sustainability, promoting resilience among farmers and enhancing their livelihoods.

Farmers suggested interventions such as value addition (e.g., processing cassava into flour or peanuts into peanut butter) and better access to markets.

**6. Sustainability and Continuation of Benefits**

**Prompt:**
*How confident are you in sustaining new farming practices in the absence of the project?*

**Expanded Response:**
Farmers expressed moderate confidence in sustaining the practices. They have established strong farmer groups and savings cooperatives that provide a foundation for continued collaboration. However, they emphasized the need for ongoing support, especially in acquiring resources such as storage facilities, processing equipment, and advanced training on climate-smart practices.

**7. Overall Satisfaction and Recommendations**

**Prompt:**
*What was the most successful part of MFEC’s intervention?*

**Expanded Response:**
The most successful aspect of MFEC’s intervention was its comprehensive approach to tackling both social and economic issues. Farmers highlighted the emphasis on group training, which fostered collaboration and peer learning. The introduction of sustainable farming practices and IGAs significantly improved livelihoods.

**Recommendations:**

To address the challenges faced by farmers and enhance agricultural productivity and sustainability in the region, the following recommendations are proposed:

1. **Provide Continued Training and Mentorship to Farmer Groups:**
   Sustained capacity-building efforts are crucial for empowering farmers with the necessary knowledge and skills. Regular training sessions can focus on modern farming techniques, efficient resource management, and sustainable practices. Mentorship programs involving experienced farmers and agricultural extension officers should also be established to ensure continuous learning and support for farmer groups.
2. **Establish Value-Addition Facilities to Process Agricultural Produce Locally:**
   Setting up facilities for value addition, such as cassava processing plants or peanut butter production units, can significantly increase the profitability of agricultural produce. By processing raw products locally, farmers can enhance the market value, reduce post-harvest losses, and create employment opportunities within their communities. These facilities can be managed through cooperatives or public-private partnerships to ensure long-term sustainability.
3. **Strengthen Market Linkages and Provide Farmers with Market Information:**
   Farmers often lack access to reliable markets, leaving them vulnerable to exploitative middlemen. Establishing robust market linkages, such as farmer-to-market direct sales platforms or partnerships with agro-processors, can help farmers secure fair prices. Additionally, providing farmers with timely market information on pricing, demand, and emerging trends can improve decision-making and enable them to align their production with market needs. Digital tools and community information hubs can be utilized to disseminate this information effectively.
4. **Advocate for Policy Alignment to Encourage Sustainable Agricultural Practices:**
   There is a need for coordinated policy efforts to promote sustainable agriculture. For instance, resolving the conflict between subsidies for chemical fertilizers and the promotion of organic alternatives can reduce confusion among farmers. Policymakers should collaborate with agricultural stakeholders, including organizations like MFEC, to ensure that policies support environmentally friendly practices and align with the broader goals of sustainable development. Advocacy efforts should also include raising awareness about the long-term benefits of organic farming and facilitating access to affordable organic inputs.

SHFs noted that by implementing these recommendations can address the root causes of farmers’ challenges while fostering sustainable agricultural practices, increasing incomes, and improving food security in the region. Collaboration between stakeholders, including farmer groups, government institutions, non-governmental organizations, and private sector actors, will be essential in achieving these goals.
