# Supplementary material for: Evaluating Effectiveness of Sustainable Livelihood Development in Rural Communities along Mara River Basin, Tanzania: What Works, What Doesn’t Work, and Why?
Source: PLoS One. 2026 Jun 11;21(6):e0351252. doi: 10.1371/journal.pone.0351252 (PMC13258000; doi:10.1371/journal.pone.0351252)
Supplement: S2 File — (ZIP) [file pone.0351252.s002.zip › FGDs Kitenga Village, Bumera Ward.docx]

## **ANNEX III: Farmers’ FGDs Checklist**

**Key Discussion Topics**

**Key Discussion Topics in Kitenga Village, Bumera Ward** **for SHFs Groups:** (i) TAITAKYA “*Shituka*” Group (ii) KAZI Group (iii) FAMOs Group (iv) JITUME Group

**1. Project Relevance and Awareness**

**Discussion Prompt:**

- Which kinds of challenges were addressed by MFEC in farming activities?

**Expanded Response:**

Mogabiri Farm Extension Centre (MFEC) significantly contributed to improving the livelihoods of farmers in Kitenga Village. Their interventions addressed various challenges in farming activities, including low agricultural productivity, poor soil fertility, limited knowledge of modern farming techniques, and challenges related to climate change. MFEC introduced training programs on **integrated farming methods, soil conservation techniques, crop diversification, and organic farming practices.**

Key interventions included:

- **Nutritional Projects in Schools:** Encouraging sustainable school farming initiatives.
- **Training in Modern Agriculture:** Providing step-by-step guidance on farm preparation, planting, soil erosion prevention, and optimal livestock management.
- **Seed Preservation and Organic Fertilizer Use:** Farmers were taught to prepare and preserve indigenous seeds and to use animal manure as a natural fertilizer to enhance soil fertility.
- **Climate Adaptation Planning:** Guidance on when to plant, water harvesting techniques, and tree planting to improve rainfall patterns.
- **Capacity Building on Entrepreneurship:** Training on marketing strategies, value addition, and small-scale business management.

The training extended to **livestock keeping, poultry management, and cooperative resource use.**

**Key Achievements:**

- Farmers adopted integrated farming and organic fertilizers, improving crop quality and yields.
- Increased community awareness of climate-resilient farming practices.
- Enhanced entrepreneurial skills, allowing farmers to market agricultural products locally and externally.
- Farmers expressed satisfaction with the project's focus on capacity building. Groups such as TAITAKYA, KAZI, and JITUME highlighted their improved ability to prepare and manage their land, conserve local seeds, and diversify crops to ensure resilience to climate shocks. They also emphasized the positive impact of VICOBA (Village Community Banking) training, which has enhanced household savings and investment capacities.

Farmers noted that the PACDR process was an eye-opener. They appreciated being involved in discussions about how climate and disaster risks impacted their livelihoods. They recognized it as a valuable platform to:

- Identify key vulnerabilities in farming practices, such as unpredictable rainfall, soil degradation, and pest invasions.
- Share indigenous knowledge on managing weather variability and disasters.
- Understand the importance of collective action in mitigating risks.

**Quotes from Farmers:**

- “PACDR ilitufanya kutambua changamoto zinazotusumbua na kufikiria hatua za kuchukua. Ilikuwa ni muhimu kuona kuwa shida hizi ni za pamoja na kuna njia za kuzitatua.”
- “Kupitia PACDR tulijifunza kuwa kilimo chetu kinaweza kuimarishwa kwa kutumia mbinu za kisasa na pia tukitunza mazingira.”

### **....Key Issues Identified During PACDR**

Farmers highlighted several critical challenges affecting their agricultural practices:

- **Climate Variability:** Unpredictable rainfall patterns affecting planting and harvesting cycles.
- **Soil Erosion:** Loss of fertile topsoil due to deforestation and poor farming techniques.
- **Pest and Disease Outbreaks:** Increased incidence of crop pests and livestock diseases.
- **Water Scarcity:** Limited access to reliable water sources for irrigation and livestock.

**Response:** Farmers were keen to address these challenges through the proposed action plan, emphasizing practices like agroforestry, crop diversification, and water conservation.

### **Participation in Action Plan Development (Mpango Kazi)**

Farmers praised the participatory approach of the action plan. They felt their voices were heard, and their suggestions were integrated. Key elements of the action plan included:

- **Climate-Resilient Farming Practices:** Training on mixed cropping, crop rotation, and use of organic fertilizers.
- **Soil and Water Conservation:** Encouragement to adopt terracing, planting cover crops, and constructing rainwater harvesting structures.
- **Livelihood Diversification:** Promotion of alternative income-generating activities (e.g., poultry keeping, beekeeping).
- **Community Collaboration:** Establishment of farmer groups to share knowledge, resources, and labor.

**Quotes from Farmers:**

- “Mpango kazi umetuwezesha kuelewa kuwa tunaweza kushirikiana kama jamii kuboresha kilimo chetu na kupambana na changamoto za hali ya hewa.”
- “Tunapenda kwamba hatua zetu zimezingatia siyo tu mahitaji yetu bali pia mazingira yetu.”

### **....Implementation and Follow-Up**

Farmers reported being optimistic about implementing the action plan but also highlighted the need for:

- Regular follow-up from extension officers to ensure adherence to best practices.
- Access to inputs such as quality seeds, organic fertilizers, and affordable tools.
- More training on financial literacy and access to credit for farming activities.

**Challenges Encountered:**

- Limited financial resources to adopt all recommended practices.
- Resistance from some community members who were hesitant to change traditional farming methods.
- A need for more demonstration farms to illustrate the benefits of the proposed interventions.

### **.....Recommendations for Sustainability**

Farmers provided feedback to ensure the sustainability of the action plan:

1. **Increased Training and Capacity Building:** Regular refresher training on climate-smart agricultural practices.
2. **Formation of Task Groups:** Designated groups to monitor progress and ensure adherence to the plan.
3. **Collaboration with Local Authorities:** Strengthened ties with government officials to access more support and funding.
4. **Incorporation of Indigenous Knowledge:** Blending modern techniques with traditional practices for holistic solutions.

**Quote from a Farmer:**

- “Mpango kazi unapaswa kufuatiliwa mara kwa mara. Tukiwa na mafunzo endelevu na usaidizi wa Mogabiri na serikali, tunaweza kuleta mabadiliko ya kweli kwenye maisha yetu.”

### **Overall Reflection**

Farmers viewed PACDR and the resulting action plan as a transformative initiative that fostered resilience against climate and disaster risks. They expressed gratitude for being included in the decision-making process and stressed the importance of continued support from stakeholders to realize long-term benefits.

**Discussion Prompt:**

- Was climate change among the challenges solved by MFEC?

**Expanded Response:**

Yes, climate change was a critical focus of MFEC's interventions. Through training and practical demonstrations, farmers were introduced to climate adaptation practices such as:

1. **Water Harvesting and Management:** Constructing retention ditches and using harvested water for irrigation.
2. **Agroforestry:** Planting drought-resistant tree species to improve microclimates and stabilize the soil.
3. **Integrated Crop and Livestock Systems:** Balancing livestock populations to minimize overgrazing and soil degradation.

The **PACDR (Program for Agricultural Climate Disaster Resilience)** has played a central role in helping farmers adapt to the changing climate. This program focuses on developing tailored action plans for each farming community, incorporating both short-term emergency responses and long-term resilience strategies. These action plans, known locally as **“Mpango Kazi”** (Action Plan), provide a structured framework for farmers to follow in the event of climate shocks or disasters.

Key components of **PACDR - Mpango Kazi** include:

- **Early Warning Systems:** Establishment of systems that give farmers advance notice of weather patterns, allowing them to take preventive measures, such as adjusting planting schedules or securing water resources.
- **Community-based Resource Management:** Promoting local involvement in decision-making processes regarding land and water management to ensure that resources are used sustainably and equitably.

By implementing the PACDR, farmers have experienced increased confidence in their ability to adapt to climate changes, resulting in more stable food production and incomes.

**Prompt:** *What did MFEC do to assist you in solving the mentioned challenges?*
**Responses:**
MFEC actively addressed climate change-related challenges by introducing climate adaptation practices. Training sessions focused on integrated crop-livestock systems, soil conservation measures, agroforestry, and water-efficient irrigation methods. These measures have helped farmers mitigate the adverse effects of erratic rainfall and prolonged droughts. MFEC’s assistance went beyond technical training; it empowered communities through hands-on demonstrations and follow-ups. For instance, MFEC:

- Established demonstration farms to model sustainable agricultural practices.
- Conducted regular training on nutrition, crop diversification, and pest control.
- Provided access to improved local chicken breeds and facilitated their management to optimize productivity.
- Supported farmers in forming and strengthening groups for collective action, enabling better resource sharing and mutual learning.
- Fostered collaboration with government extension officers to ensure ongoing technical support for SHFs.

**2. Participation and Engagement**

**Discussion Prompt:**

- Was the training on the challenges addressed by MFEC inclusive of both men and women?

**Expanded Response:**

MFEC ensured inclusivity by engaging both men and women in training and decision-making processes. Community leaders and government extension officers collaborated to create gender-balanced programs. Women’s involvement was particularly emphasized in areas like:

- Food preservation and nutrition.
- Management of community-based savings groups (VICOBA).
- Participation in decision-making forums for resource allocation.

The active involvement of women enhanced their confidence and leadership skills, enabling them to take on prominent roles in their households and the community.

**Impact of Inclusivity:**

- Women gained financial literacy and entrepreneurial skills, contributing to household incomes.
- Increased gender equality in community decision-making processes.

**3. Effectiveness and Practical Impact**

**Discussion Prompt:**

- Were there any farming practices introduced by MFEC during the training process? Which ones were adopted? How effective were they?

**Expanded Response:**

MFEC introduced various innovative farming practices, including:

1. **Mixed Cropping and Crop Rotation:** Enhancing soil fertility and reducing pest infestations.
2. **Organic Farming Techniques:** Promoting the use of manure and compost.
3. **Climate-Smart Agriculture:** Techniques like planting drought-tolerant crops and water-efficient irrigation.

Farmers reported significant improvements in productivity and sustainability after adopting these practices. For example, crop yields increased by approximately 40%, and farming costs were reduced by minimizing chemical inputs.

**Prompt:** What climate adaptation practices have you integrated, and how effective have they been?
**Responses:**
Farmers integrated practices like tree planting, mulching, and intercropping to combat soil erosion and retain moisture. Organic manure application was adopted to improve soil fertility without degrading the environment. Diversified crop production also reduced risks from crop failure due to unpredictable weather. Farmers testified that these practices had increased their resilience to climate variability, with some noting an improvement in crop yields even during dry spells.

**4. Income Diversification and Economic Benefits**

**Discussion Prompt:**

- Are you engaging in other income-generating activities (IGAs)? What benefits have they provided?

**Expanded Response:**

Farmers diversified their income sources through:

1. **Small-Scale Businesses:** Selling surplus crops, running small shops, and trading farm produce.
2. **Livestock Keeping:** Rearing poultry, goats, and cattle for meat, milk, and manure production.
3. **Community Savings Groups:** Engaging in collective lending and borrowing schemes to finance IGAs.

**Benefits Gained:**

- Increased household income.
- Financial stability through access to affordable loans.
- Improved food security and nutrition.

**5. Challenges and Barriers**

**Discussion Prompt:**

- What challenges have you faced in adopting new farming practices?

**Expanded Response:**

Despite the success of MFEC’s interventions, some challenges persisted, including:

1. **Limited Access to Indigenous Seeds:** Farmers faced difficulties in obtaining and preserving high-quality seeds.
2. **Time Constraints:** Preparing organic fertilizers like manure required significant time and effort.
3. **Knowledge Gaps:** Some farmers needed refresher training to fully grasp and implement new techniques.

Recommendations:

- Establish a centralized seed bank for indigenous seeds.
- Provide regular follow-up training and mentorship programs.

**6. Sustainability and Continuation of Benefits**

**Discussion Prompt:**

- How confident are you in sustaining new farming practices in the absence of the project?

**Expanded Response:**

Farmers expressed confidence in sustaining the practices introduced by MFEC. Through the **train-the-trainer (FAMOs) approach,** community members have developed the capacity to continue training others. This ensures that the knowledge and skills gained will be passed down, promoting sustainability.

**7. Overall Satisfaction and Recommendations**

**Discussion Prompt:**

- What was the most successful part of MFEC in solving community challenges? What are your recommendations?

**Expanded Response:**

**Most Successful Areas:**

- Enhancing agricultural productivity through climate-resilient practices.
- Promoting entrepreneurship and financial inclusion.
- Building strong farmer groups that fostered unity and resource sharing.

**Recommendations:**

1. Expand the scope of future projects to address other social issues, such as gender-based violence and child education disparities.
2. Provide advanced training on indigenous seed production and preservation.
3. Facilitate participation in agricultural fairs to showcase local products.
4. Increase the frequency of follow-up training sessions.

MFEC project has made a lasting impact on the community, equipping farmers with valuable skills and fostering sustainable development. Farmers are optimistic about continuing their journey toward improved livelihoods and agricultural productivity.
