# Supplementary material for: Evaluating Effectiveness of Sustainable Livelihood Development in Rural Communities along Mara River Basin, Tanzania: What Works, What Doesn’t Work, and Why?
Source: PLoS One. 2026 Jun 11;21(6):e0351252. doi: 10.1371/journal.pone.0351252 (PMC13258000; doi:10.1371/journal.pone.0351252)
Supplement: S2 File — (ZIP) [file pone.0351252.s002.zip › FGDs Surubu Village, Komaswa Ward.docx]

## **ANNEX III: Farmers’ FGDs Checklist**

**Project Final Evaluation on “*Sustainable Livelihood Development of Rural Communities along the Mara River Basin, Tarime District, Tanzania*”**

**Key Discussion Topics in Surubu Village, Komaswa Ward** **for SHFs Groups:** (i) WASU Group “*Wanawake Surubu*” (ii) MUUNGANO Group (iii) FAMOs Group

**1. Project Relevance and Awareness**

**Discussion Prompt:**

- *Which kinds of challenges were addressed by MFEC in farming activities?*

**Responses:**
Mogabiri Farm Extension Centre (MFEC) is recognized for addressing multiple challenges faced by farmers in Surubu Village. Farmers highlighted several key areas where MFEC’s interventions made significant contributions:

1. **Improved Agricultural Practices:**
   - Farmers were trained on sustainable farming methods, such as intercropping, contour farming, and the use of organic fertilizers. This addressed issues of poor soil fertility and low productivity.
   - Groups like MUUNGANO reported adopting organic manure over synthetic fertilizers and planting trees to curb soil erosion and mitigate climate change.
   - WASU group members were introduced to improved sorghum seeds and proper maize planting techniques, which enhanced yields and storage.
2. **Entrepreneurship and Financial Skills:**
   - Farmers were trained on forming and managing Village Community Banks (VICOBA), enabling them to save, borrow, and invest in farming inputs and other income-generating activities.
   - WASU initiated tailoring and soap-making projects, which provided stable incomes for group members.
3. **Climate Change Adaptation:**
   - MFEC educated farmers on the importance of reforestation and reducing deforestation. Groups were encouraged to plant trees to attract rainfall and combat environmental degradation.
4. **Gender-Based Violence (GBV) Awareness:**
   - Through gender-focused seminars, communities were empowered to reduce GBV by encouraging equal participation in decision-making and economic activities. Women in WASU noted reduced dependency on their spouses, contributing to harmony within households.

Farmers emphasized that the MFEC training had transformed their livelihoods, making farming more productive and community groups more resilient. They expressed a desire for continued support in accessing improved seeds and tools, as these remained a challenge.

**2. Participation and Engagement**

**Discussion Prompts:**

- *Was the training on the challenges addressed by MFEC inclusive of both men and women?*
- *How useful were the training sessions in solving community challenges?*
- *Did women’s involvement in training contribute to their skills and participation in community decision-making?*

**Responses:**
MFEC training sessions were inclusive, targeting both men and women. Women’s participation led to:

1. **Enhanced Skills:**
   - Women in WASU and MUUNGANO learned new skills in crop and poultry farming, entrepreneurship, and financial management, empowering them to contribute effectively to household income.
   - Increased confidence in managing IGAs like tailoring, soap-making, and food processing.
2. **Improved Community Dynamics:**
   - With training, men became more supportive of women’s involvement in income-generating activities, reducing domestic tensions and GBV incidents.
   - Women began participating in community decisions, fostering gender equality in leadership roles.

**Impact Narrative:**
The inclusiveness of the training improved household welfare and strengthened community cohesion. However, farmers requested more specialized training on poultry rearing and beekeeping for both genders to address existing gaps.

**3. Effectiveness and Practical Impact**

**Discussion Prompts:**

- *Were there any farming practices introduced by MFEC?*
- *Which ones did you adopt in your farming process, and how effective were they?*

**Responses:**
MFEC introduced practices such as:

1. **Crop Rotation and Mixed Cropping:** Improved soil fertility and reduced pest infestations.
2. **Use of Organic Fertilizers:** MUUNGANO adopted compost-making techniques, which significantly improved maize yields.
3. **Agroforestry:** Planting trees around farmlands minimized soil erosion and enhanced biodiversity.
4. **Poultry Farming Techniques:** Farmers were trained on disease prevention and feeding, increasing poultry survival rates.

Farmers reported that these practices were effective in boosting productivity and ensuring environmental sustainability.

**4. Income Diversification and Economic Benefits**

**Discussion Prompts:**

- *What income-generating activities (IGAs) are you engaged in apart from farming?*
- *What benefits have you gained from these IGAs?*

**Responses:**
Farmers engaged in diverse IGAs, including:

1. **Tailoring and Soap-Making (WASU):** Increased income through the sale of school uniforms and soap products.
2. **Beekeeping:** Farmers started honey production to complement their income.
3. **Small Businesses:** Individual members ventured into petty trading of farm produce and household goods.

**Benefits:**

- Additional household income reduced financial dependency and improved the standard of living.
- Strengthened group savings allowed members to access loans for emergencies or business expansion.

**5. Challenges and Barriers**

**Discussion Prompts:**

- *What challenges have you faced in adopting new farming practices?*

**Responses:**
Challenges included:

1. **Access to Inputs:** Farmers struggled to obtain high-quality seeds and affordable tools.
2. **Livestock Health:** Poultry farming faced setbacks due to disease outbreaks and limited access to veterinary services.
3. **Knowledge Gaps:** Some farmers found it difficult to fully implement new techniques without additional follow-up training.

**Proposed Solutions:**
Farmers suggested MFEC should facilitate better access to improved seeds, conduct refresher training, and establish a veterinary service hub in the community.

**6. Sustainability and Continuation of Benefits**

**Discussion Prompt:**

- *How confident are you in sustaining new practices in the absence of the project?*

**Responses:**
Farmers expressed confidence in sustaining the practices due to the knowledge gained. They emphasized:

1. Forming stronger ties with local government for ongoing support.
2. Continuing group activities to share skills and resources.
3. Applying VICOBA principles to sustain investments in farming and IGAs.

**7. Overall Satisfaction and Recommendations**

**Discussion Prompts:**

- *What was the most successful part of MFEC in solving community challenges?*
- *What advice would you give to MFEC?*

**Responses:**
**Most Successful Aspects:**

- Training on climate-resilient farming and IGAs was transformative, leading to increased income and food security.

**Recommendations:**

- Provide more frequent and specialized training on livestock management and sustainable farming.
- Improve access to inputs such as seeds and tools.
- Strengthen follow-up mechanisms to ensure continued support and monitoring.
- Enhance communication between MFEC and farmers to address emerging challenges quickly.

The maize demonstration plots and tree seedling programs have been among the most successful initiatives supported by the Mogabiri Farm Extension Centre (MFEC). However, farmers have raised requests for additional high-quality maize seeds and tree seedlings to enhance productivity and address agricultural and environmental challenges.

### Needs and Recommendations

**Maize Demonstration Plots:**

- Farmers recommend strengthening the maize demonstration plot programs to provide more opportunities for learning advanced techniques in planting, maintaining, and harvesting maize.
- There is a significant demand for high-yield maize seeds that are drought-tolerant and resistant to diseases. This is particularly critical in the Surubu area, which often faces challenges related to climate change.

**Tree Seedlings:**

- There is a strong need for increased provision of tree seedlings, particularly those that offer multiple benefits, such as fruit trees, soil conservation trees, and trees that attract rainfall.
- Farmers propose that training programs on tree planting and care should be emphasized to ensure the trees grow successfully and meet their intended objectives.

### Expected Benefits

- **Improved maize production**, contributing to better nutrition and food security.
- **Reduced soil erosion** through tree planting and enhanced soil fertility.
- **Increased income** from fruit harvests and higher maize yields.

### Recommendations for MFEC

1. **Enhance Seed Distribution:**
   - Provide high-quality maize seeds and tree seedlings to farming groups to meet the growing demand.
2. **Expand Demonstration Plots:**
   - Establish additional maize demonstration plots and provide specialized training on tree management practices.
3. **Conduct Close Monitoring:**
   - Follow up on the progress of farming and tree-planting projects to assess challenges and provide technical assistance to farmers.

By addressing these needs, farmers anticipate continuing to achieve significant progress in sustainable agriculture and improving the environmental health of the Surubu area.

Farmers expressed overall satisfaction with MFEC’s contributions to their livelihoods. They valued the participatory approach, which empowered them to implement sustainable practices and improve household welfare. Farmers encouraged continued collaboration to ensure long-term success.
