# Supplementary material for: Evaluating Effectiveness of Sustainable Livelihood Development in Rural Communities along Mara River Basin, Tanzania: What Works, What Doesn’t Work, and Why?
Source: PLoS One. 2026 Jun 11;21(6):e0351252. doi: 10.1371/journal.pone.0351252 (PMC13258000; doi:10.1371/journal.pone.0351252)
Supplement: S2 File — (ZIP) [file pone.0351252.s002.zip › FGDs Turugeti Village, Bumera Ward.docx]

**ANNEX III: Farmers’ FGDs Checklist**

**Project Final Evaluation on “*Sustainable Livelihood Development of Rural Communities along the Mara River Basin, Tarime District, Tanzania*”**

**Key Discussion Topics in Turugeti Village, Bumera Ward** **for SHFs Groups:** (i) MKOMBOZI Group (ii) VICOBA Group (iii) UPATU Group (iv) FAMOs Group

**1. Project Relevance and Awareness**

**Discussion Prompt:**
**Q: Which kinds of challenges were addressed by MFEC in farming activities?**
**Responses:**
Mogabiri Farm Extension Centre (MFEC) has played a transformative role in addressing various challenges that farmers in Turugeti village have faced. MFEC has focused on improving agricultural productivity by teaching sustainable farming techniques, including better crop management, soil conservation, and organic fertilizer usage. Additionally, the center has introduced innovative practices such as bee farming, fish farming, tree planting, and poultry keeping, tailored to address specific environmental and economic issues.

Through its awareness programs, MFEC has educated us on the importance of conserving indigenous seeds, using organic manure like compost and cow dung, and adopting climate-resilient practices such as planting drought-resistant crops. For example, farmers now practice row planting with accurate spacing to optimize water usage and increase yields. The introduction of "shamba darasa" (demonstration farms) has been a game-changer, enabling us to implement these practices effectively.

Beyond farming, MFEC has been instrumental in fostering community cohesion through group formations such as VICOBA, MKOMBOZI, and FAMOs, which have empowered us economically and socially. These groups have not only facilitated access to financial resources but also strengthened social ties and mutual support within the community.

Therefore, MFEC addressed multiple challenges through comprehensive training and practical solutions, focusing on:

- **Agricultural Development:**
  - Promoting improved farming techniques, including the use of organic fertilizers like compost and manure instead of chemical fertilizers, which deplete soil nutrients.
  - Demonstrating climate-resilient farming practices, such as short-season seed varieties and precision planting methods.
- **Livestock Management:**
  - Educating on sustainable livestock rearing, including poultry and fish farming.
  - Training on beekeeping for income diversification.
- **Climate Change Adaptation:**
  - Teaching climate-smart agriculture and methods to address drought, unpredictable rainfall, and soil erosion.
  - Mapping village resources to enhance management of roads and water retention infrastructure during extreme weather events.
- **Group Management and Livelihoods:**
  - Facilitating the formation and management of groups like VICOBA and FAMOs to enhance financial stability and community solidarity.
  - Teaching group dynamics, fund management, and creating shared goals for sustainable livelihoods.
- **Environmental Conservation:**
  - Encouraging tree planting and natural resource conservation to combat deforestation and land degradation.

The project has significantly improved participants’ knowledge of farming, group management, and climate adaptation. Many noted that MFEC introduced innovations like community demonstration farms and native seed preservation, which have been transformative for local farmers.

**Q: What did MFEC do to assist you in solving the mentioned challenges?**
MFEC provided practical and theoretical training on sustainable agriculture and climate adaptation techniques. These trainings addressed our key challenges, such as soil fertility depletion, erratic rainfall, and pest infestations. For instance, the center promoted the use of organic fertilizers instead of industrial ones, which often degrade soil quality. MFEC also distributed indigenous drought-tolerant seeds, helping farmers cope with unpredictable weather patterns.

Moreover, MFEC encouraged the construction of water-harvesting structures and proper road maintenance using locally available materials to mitigate the effects of heavy rainfall and soil erosion. They also facilitated access to resources like roofing materials and financial support for group-based projects such as poultry farming.

MFEC provided tailored solutions by:

- Delivering hands-on training in sustainable practices.
- Distributing native seeds adapted to local conditions.
- Providing continuous mentorship on farm productivity and group leadership.

**Q: Was climate change among the challenges solved by MFEC?**
Yes, MFEC actively addressed climate change challenges by integrating climate adaptation strategies. Climate change was a significant focus. MFEC trained farmers on climate-smart agricultural practices such as early planting, short-season crops, and agroforestry. Farmers were also taught how to create village maps for better resource management and mitigate climate-related risks, such as droughts and floods.

**Q: What climate adaptation practices have you integrated?**
Farmers have integrated several practices, such as planting drought-tolerant crops, using mulching to retain soil moisture, and constructing water storage systems. These methods have proven effective, with farmers reporting increased yields even in adverse weather conditions. Additionally, agroforestry has contributed to soil conservation and increased shade cover, which has mitigated the effects of heatwaves.

Farmers have adopted:

- Short-cycle crops resistant to erratic weather patterns.
- Manure-based fertilization to improve soil fertility sustainably.
- Resource mapping to manage village infrastructure and mitigate climate risks.

**Q: How effective have they been?**
Farmers reported improved yields and resilience to climatic shocks. The techniques introduced were practical, leading to better food security and environmental conservation.

**2. Participation and Engagement**

**Discussion Prompt:**
**Q: Was the training on challenges addressed by MFEC inclusive of both men and women?**
**Responses:**
Yes, the training was inclusive and encouraged active participation from both genders. Men and women were equally involved in discussions, hands-on demonstrations, and decision-making activities. The training sessions were inclusive, ensuring equal participation of both men and women. This inclusivity strengthened community involvement and ensured that knowledge and skills were shared equitably across genders.

**Q: How useful were the trainings in solving community challenges?**
The training was highly impactful. Farmers now practice crop rotation and better seed storage techniques, which have significantly reduced post-harvest losses. Women, in particular, gained skills in leadership and financial management, enabling them to play active roles in household and community decision-making processes.

The training programs were highly useful as they:

- Equipped participants with knowledge to solve immediate and long-term challenges.
- Encouraged community-driven solutions and collaboration.
- Increased household income through better agricultural and group practices.

**Q: Did women’s involvement contribute to skill development and decision-making?**
Women reported gaining confidence and skills in areas like:

- Financial literacy through VICOBA.
- Agricultural decision-making within their households.
  This has fostered greater inclusion of women in community leadership roles.

**3. Effectiveness and Practical Impact**

**Discussion Prompt:**
**Q: Were there any farming practices introduced by MFEC during the training?**
**Responses:**
MFEC introduced practices such as compost manure preparation, agroforestry, and integrated pest management. Farmers adopted these practices, and the results have been promising. For example, organic manure has improved soil fertility, while agroforestry has enhanced environmental sustainability and diversified income through tree products.

MFEC introduced practices such as:

- Use of compost manure for long-term soil health.
- Improved planting methods like row planting and proper spacing.
- Native seed storage and preservation for sustainability.

**Q: Which practices have you adopted? How effective were they?**
Farmers widely adopted composting and improved planting methods, reporting better yields and reduced input costs.

**4. Income Diversification and Economic Benefits**

**Discussion Prompt:**
**Q: Are you also engaging in other income-generating activities (IGAs)?**
**Responses:**
Farmers have diversified their incomes by engaging in beekeeping, poultry farming, and small-scale businesses. For example, women have excelled in soap-making and tailoring, while men have ventured into fish farming and carpentry. These activities have not only improved household incomes but also reduced over-dependence on farming.

IGAs initiated include:

- Beekeeping and honey production.
- Poultry farming.
- Small-scale trading facilitated by market days.

**Q: What benefits did you gain from the IGAs?**

- Additional income to meet family needs.
- Reduced reliance on single sources of income, increasing financial stability.
- Enhanced community cohesion through group activities.

**5. Challenges and Barriers**

**Discussion Prompt:**
**Q: Did you receive training on new farming practices?**
**Responses:**
Challenges include limited access to capital, insufficient training on organic manure preparation, and a lack of adequate livestock to produce sufficient manure. These constraints have slowed the adoption of sustainable practices, leading to suboptimal productivity in some cases. Farmers also cited high-interest rates on loans as a barrier to scaling up farming activities. Challenges remain, including:

- Limited access to credit due to high-interest rates and inadequate collateral.
- Insufficient training on manure production and preservation of organic fertilizers.
- Limited resources for scaling up poultry and beekeeping activities.

**6. Sustainability and Continuation of Benefits**

**Discussion Prompt:**
**Q: How confident are you in sustaining the new practices in the absence of the project?**
**Responses:**
Farmers expressed confidence in sustaining the new practices due to the hands-on training received and the strong community support networks established by MFEC. However, they emphasized the need for continuous follow-up and periodic refresher training.

Farmers expressed confidence due to the practical skills gained and the establishment of self-sustaining groups. However, they emphasized the need for ongoing support in technical training and resource mobilization.

**7. Overall Satisfaction and Recommendations**

**Discussion Prompt:**
**Q: What was the most successful part of MFEC in solving community challenges?**
**Responses:**
The most successful aspect of MFEC’s work was its holistic approach, addressing both environmental and socio-economic challenges. The establishment of VICOBA groups and the introduction of climate-smart agricultural practices were particularly impactful, fostering resilience and self-reliance among farmers.

The most successful aspects were:

- Empowering communities with practical knowledge.
- Enhancing group cohesion and financial independence through VICOBA.
- Introducing sustainable farming practices and climate adaptation strategies.

**Q: What would be your advice to MFEC?**
Farmers recommend expanding access to credit for men and women equally and providing additional training on advanced farming techniques. They also suggest greater collaboration with government agencies to ensure the sustainability of project benefits.

Farmers recommended:

- Expanding training on manure production and preservation techniques.
- Strengthening partnerships with local governments to enhance resource mobilization.
- Increasing focus on vulnerable groups and equitable participation in all activities.

The groups highlights MFEC's significant impact on sustainable livelihoods and community resilience, while also outlining areas for further development.
