# Supplementary material for: Evaluating Effectiveness of Sustainable Livelihood Development in Rural Communities along Mara River Basin, Tanzania: What Works, What Doesn’t Work, and Why?
Source: PLoS One. 2026 Jun 11;21(6):e0351252. doi: 10.1371/journal.pone.0351252 (PMC13258000; doi:10.1371/journal.pone.0351252)
Supplement: S2 File — (ZIP) [file pone.0351252.s002.zip › Government Official Round Table Discussion.docx]

**Round Table Discussion on the Project Final Evaluation at Tarime District Council**

**1. Project Activities and Outputs**

**Q: What do you understand about Mogabiri and the services they provide in your area?**

**Response:**
Mogabiri is one of the key development partners working with us to ensure that community services align with their specific needs.

The collaboration between the government and local stakeholders, such as Mogabiri, has been instrumental in delivering agricultural and livestock extension services in Tarime District. These services include:

1. **Training on Sustainable Agricultural Practices**
   - Farmers receive hands-on training focused on sustainable practices to enhance productivity. MFEC serves as an advisory hub, providing knowledge on improved crop production, livestock management, climate change adaptation, and environmental management. Farmers receive hands-on training focused on sustainable practices to boost productivity. Mogabiri Farm Extension Centre (MFEC) serves as a vital advisory hub, offering expertise in improved crop production, livestock management, climate change mitigation, and environmental conservation.
2. **Gender-Based Violence (GBV) Awareness**
   - Mogabiri conducts training on GBV issues, particularly focusing on Female Genital Mutilation (FGM) in schools and the wider community. Mogabiri provides training on GBV, particularly addressing Female Genital Mutilation (FGM) in schools and communities.
3. **Formation and Strengthening of VICOBA Groups**
   - The project supports the establishment and capacity building of VICOBA groups. Many of these groups are now actively engaged in savings and credit activities, empowering farmers through accessible loans (up to three times their contributions) and financial management training. The project has established and empowered Village Community Banks (VICOBA) groups, enabling them to engage in savings and credit activities. Members can access loans (up to three times their contributions) and receive financial management training, fostering economic resilience.
4. **Promotion of Income-Generating Crops**
   - MFEC introduced bananas as a cash crop, collaborating with stakeholders to develop *KIMAWAZO* Banana Market. This initiative has encouraged farmers to adopt this high-value crop.
5. **Community and School Nutrition Programs**
   - The introduction of a school feeding program has significantly improved student attendance, reduced dropouts (particularly among girls), and enhanced academic performance. This success has inspired government plans to make the program mandatory in all primary schools.
6. **Collaboration with Government Officials**
   - MFEC works closely with government officials, sharing plans to align interventions with community needs. This collaboration ensures efficient resource utilization and avoids duplication of services.
7. **Infrastructure and Resource Support**
   - Government trust in the project has led to contributions such as donating a sunflower oil extraction machine to a farmer group. This partnership optimizes resources and promotes sustainability.

**Q: Are there any observable changes after Mogabiri’s interventions in your area?**

**Response:**
Yes, there have been significant changes in the community:

- **Active Participation:**
  - Farmers have adopted the People-Owned Process (POP) approach, which fosters a sense of ownership and self-reliance through active engagement in planning and implementation. The People-Owned Process (POP) approach has enhanced farmers' engagement in planning and implementation, fostering ownership and self-reliance. Example of the POP Approach in Practice; In rural agriculture development programs by MFEC, for example, the POP approach involved farmers’ groups actively participating in identifying the key challenges they face in production, such as water scarcity or soil degradation. The community then collaboratively designs solutions, such as rainwater harvesting techniques or organic farming practices, and takes the lead in implementing and managing these strategies. Additionally, regular meetings are held to assess progress, share experiences, and adapt strategies based on community feedback.

Therefore, the People-Owned Process (POP) approach is a community-driven, participatory methodology that emphasizes the active involvement of local communities in the design, implementation, and evaluation of development programs or initiatives. This approach ensures that the people who are directly impacted by development interventions are not only beneficiaries but also key decision-makers throughout the process. The primary focus of POP is on empowering communities by recognizing their knowledge, needs, and resources, thereby promoting sustainable and contextually relevant solutions.

- **Behavioural Change:**
  - Sustainable agricultural practices have enhanced household food security and income levels. For instance, VICOBA-trained groups report improved financial management and timely loan repayments.
- **Challenges in Adoption:**
  - Some groups faced challenges such as limited resources for post-harvest management and marketing. Collaboration with institutions like Sokoine University of Agriculture (SUA) is recommended to address these gaps.

**Q: How do you involve other stakeholders, such as Mogabiri, in the planning process?**
**Response:**
We regularly hold meetings with all stakeholders, including Mogabiri, where they share their plans. These plans are documented in our reports, helping us identify service gaps and direct efforts to areas not yet covered by other stakeholders.

**Q: How does Mogabiri involve the Local Government Authority (LGA) in their activities?**
**Response:**
Mogabiri collaborates with local government officials by informing them about their mission before starting community interventions. As providing community services is a government responsibility, Mogabiri’s support is highly valued. Despite challenges such as staffing and transportation limitations, we ensure collaboration and provide assistance where possible.

**Q: Do you face any challenges working with Mogabiri?**
**Response:**
Yes, some challenges include:

- **Staffing Gaps:**
  - A lack of staff in key areas, such as environmental management, limits collaboration. This creates a perception that Mogabiri is closer to the community than the government.
- **Limited Inclusion of Government Professionals:**
  - Some training programs, such as those on climate change, do not involve relevant government professionals, possibly due to staffing gaps.
- **Exclusion of Certain LGA Departments:**
  - Mogabiri does not consistently include all LGA departments, such as the planning department, which affects the level of support they could receive.

**Q: What advice would you give Mogabiri to improve their services?**
**Response:**

1. **Focus on Value Addition and Market Linkages:**
   - Mogabiri should assist in identifying markets and educating the community on product processing, packaging, and sales. For example, communities could produce their own biscuits rather than relying on imports.
2. **Involve All LGA Departments:**
   - Engaging all relevant LGA departments will enhance collaboration and allow for broader support from professionals.
3. **Stakeholder Meetings:**
   - Regular meetings with stakeholders will help sustain implemented projects, provide a comprehensive picture of successes and challenges, and promote efficient resource utilization.
