# Supplementary material for: Evaluating Effectiveness of Sustainable Livelihood Development in Rural Communities along Mara River Basin, Tanzania: What Works, What Doesn’t Work, and Why?
Source: PLoS One. 2026 Jun 11;21(6):e0351252. doi: 10.1371/journal.pone.0351252 (PMC13258000; doi:10.1371/journal.pone.0351252)
Supplement: S2 File — (ZIP) [file pone.0351252.s002.zip › Manager.docx]

**ANNEX VII: Interview with the Project Manager Checklist**

**Project Final Evaluation: *“Sustainable Livelihood Development of Rural Communities Along Mara River Basin, Tarime District, Tanzania”***

***Question 1*: Project Overview and Achievements**

**Key Achievements of the Project:**
The Project Manager at Mogabiri Farm Extension Centre (MFEC) identified several notable achievements:

- **Improved Livelihoods:**
  The project significantly enhanced community livelihoods by adopting a **demand-driven approach** that encouraged mindset and behavioural change among smallholder farmers (SHFs). Improvements were evident in food security, where families shifted from consuming one meal daily to three meals. Moreover, there was a marked improvement in the overall well-being of the households, with better nutrition for children and increased school enrolment, especially for girls. A marked reduction in food wastage and enhanced food preservation techniques have ensured year-round food availability. Previously, households were limited to one meal a day, but many now enjoy three meals daily. Furthermore, we have successfully formed and strengthened smallholder farmer (SHF) groups. SHFs groups simplify challenges, making them easier to address, while fostering personal growth, discipline, and collaboration. Economic groups also encourage saving and planning for better livelihoods. Successful groups emerge from members’ shared initiative, with a clear purpose, common vision, measurable goals, commitment, and a guiding constitution.
- **Empowering Women and Addressing Gender Dynamics:**
  Women’s empowerment initiatives provided significant benefits, including entrepreneurship training and increased financial independence. However, this progress occasionally caused friction within households, as some men felt sidelined. To mitigate these challenges, the project began integrating men into development plans to foster inclusive growth and address **gender dynamics constructively.** Women’s empowerment programs have seen women actively engage in income-generating activities, such as soap making and poultry farming. However, these changes have sometimes disrupted household gender dynamics, with men expressing feelings of marginalization. The project has responded by involving men in development planning to foster inclusivity and shared responsibilities.
- **Climate Change Adaptation and Mitigation:**
  The project utilized the ***Participatory Assessment of Climate Change and Disaster Risks (PACDR)*** tool to support SHFs in addressing climate risks. This tool improved SHFs’ capacity to use climate information systems for preparing resilient farming practices. The project also promoted the use of **environmentally friendly trees** and discouraged eucalyptus planting near water sources and farms. The introduction of the PACDR tool for participatory climate change and disaster risk assessments has empowered communities to utilize climate information effectively. Farmers now adopt better land preparation methods and select environmentally friendly tree species for reforestation efforts.
- **Diversification of Livelihoods:**
  The project trained SHFs on diverse income-generating activities (IGAs), such as poultry farming, VICOBA groups, and small businesses. These initiatives aimed to improve household resilience against economic shocks. The seedbank system established in collaboration with SAT Morogoro and PELUM promoted the use of indigenous seed varieties for better productivity. The project promoted the use of indigenous seed varieties like maize, groundnuts, beans, cassava, sweet potatoes, banana seedling, vegetables, and sorghum. Collaborations with PELUM Morogoro established seed banks, enhancing sustainable farming practices.
- **Addressing Environmental Sustainability:**
  In the effort to promote environmental sustainability, several initiatives were implemented to encourage sustainable agricultural practices that minimize environmental degradation while enhancing productivity. Key strategies included:

1. **Organic Fertilizers:** Farmers were encouraged to adopt organic fertilizers, which help improve soil fertility without harming the environment. These fertilizers, which can be sourced from locally available materials such as compost or manure, contribute to better soil structure, retain moisture, and support sustainable crop production.
2. **Cover Crops:** The use of cover crops was promoted to protect the soil from erosion, improve its nutrient content, and enhance water retention. Cover crops such as legumes not only enrich the soil with nitrogen but also provide a protective layer, reducing the impact of harsh weather conditions like heavy rains or droughts.
3. **Natural Pest Control:** Farmers were introduced to natural pest control methods, which reduce reliance on harmful chemical pesticides. Local solutions like ash, **bangipori** (wild cannabis), **pilipili** (pepper), **miti midogo inayotoa maziwa** (small trees that exude milky sap), and **natural aloe vera** were encouraged as effective and eco-friendly ways to deter pests and protect crops. Additionally, **livestock urine** was utilized as a natural pesticide, further promoting sustainability while reducing chemical usage.
4. **Afforestation Efforts:** Afforestation was a key component of the sustainability agenda, with the distribution of fruit tree seedlings to local farmers. These trees help combat deforestation, provide shade for crops, enhance biodiversity, and improve soil quality. Moreover, the fruits from these trees offer an additional source of income and nutrition for the community, supporting both environmental and economic resilience.

- **Agricultural Support and Capacity Building:**
  The project prioritized capacity building for smallholder farmers (SHFs) by equipping them with the necessary knowledge and skills to improve agricultural practices and manage challenges effectively. Several key initiatives were implemented to enhance the capacity of farmers in the following areas:

1. **Training on Post-Harvest Management and Value Addition:** A series of training sessions were conducted to educate farmers on post-harvest management techniques, focusing on the importance of proper handling, storage, and processing of agricultural products to minimize losses and improve quality. Farmers learned techniques for extending the shelf life of crops, particularly for perishable items like fruits and vegetables. Additionally, value addition strategies were introduced, such as processing cassava into flour or peanuts into peanut butter, which provide farmers with opportunities to increase their income and enhance product value.
2. **Demonstration Farms for Crop Disease Management:** To address the challenges posed by crop diseases, demonstration farms were established to showcase effective management techniques for prevalent diseases, such as cassava mosaic disease and banana streak virus. These demonstration farms provided farmers with practical examples of how to detect, treat, and prevent these diseases, promoting healthier crops and reducing losses. By incorporating these hands-on training methods, farmers were able to observe the direct impact of disease management practices on crop yields and quality.
3. **Improved Planting Techniques and Seed Quality:** The project also focused on improving planting techniques by providing training on **Njia za upandaji** (planting methods) and the importance of using **mbegu bora** (quality seeds). Farmers were taught how to identify high-quality seeds and select the best varieties for their specific agro-ecological zones. Training on seed selection enabled farmers to understand the importance of choosing the right seeds to enhance yields and minimize crop failures. Additionally, farmers were educated on the names and characteristics of different seed varieties, helping them to make informed decisions about what to plant based on local climate and soil conditions.

**Recommendations:**

1. **Strengthening Market Linkages:** Future projects should prioritize the establishment of stronger market linkages to enable smallholder farmers (SHFs) to sell their produce directly, bypassing exploitative middlemen. This can be achieved by creating collection centers or cooperative-based systems where farmers can aggregate their produce, ensuring fair prices and reducing reliance on intermediaries. Strengthening these linkages will help farmers secure better deals, enhance income stability, and foster market-driven agricultural practices. Additionally, providing access to digital platforms or market information systems would support farmers in identifying demand trends and optimizing sales strategies.
2. **Reviving Partnerships with Research Institutions:** Collaborations with research institutions, such as Lyamungo Research Institute, Ukiliguru Research Centre, and Serian Research Institute in Arusha, should be revitalized to ensure the availability of high-quality seed varieties. These institutions have the expertise and resources to conduct research and breed crops that are resilient to pests, diseases, and climate change. By working with these research bodies, future projects can ensure that SHFs have access to improved seed varieties that are more productive and suited to the changing climate conditions. Additionally, partnerships can facilitate the development of tailored extension services, helping farmers adopt the best practices in crop management.
3. **Promoting Farmer-Focused Capacity Building:** In addition to market linkages and seed quality, future initiatives should continue to prioritize capacity building for farmers, focusing on not only technical farming skills but also business management, financial literacy, and entrepreneurial skills. These training programs should be designed to help farmers understand the entire value chain, from production to marketing, and help them develop sustainable, profitable business models.

 **Enhancing Infrastructure for Storage and Processing:** Future projects should also work on building the necessary infrastructure for farmers, particularly storage and processing facilities. This will help reduce post-harvest losses, especially for perishable crops like bananas and avocados, and will enable value addition, which can further increase farmers' profitability. Establishing storage facilities at collection points will ensure that farmers can store their produce safely until the right market opportunities arise.

***Question 2*: Climate Change Adaptation and Smallholder Farmers**

**Climate and Weather Information Use:**

- The project played a crucial role in supporting smallholder farmers (SHFs) by disseminating essential climate and weather information. Through targeted workshops and the use of local extension workers, farmers were equipped with timely, location-specific weather updates that enabled them to make informed decisions about their farming activities. This information helped SHFs to plan better for unpredictable weather patterns, which are becoming more frequent due to climate change.

One of the key tools utilized in the project was the **Participatory Assessment of Climate Change and Disaster Risks (PACDR)** tool. This tool proved to be instrumental in helping SHFs not only understand the climate risks they face but also integrate this knowledge into their daily farming schedules. With the assistance of the PACDR tool, farmers were able to forecast weather patterns and plan for events such as droughts or floods, thereby reducing vulnerability to crop failure.

The PACDR tool was particularly beneficial in guiding farmers in addressing soil erosion and land restoration. By using the tool, SHFs learned to incorporate soil conservation techniques into their farming practices, such as contour farming, mulching, and planting cover crops. These techniques helped mitigate soil erosion, improve soil fertility, and prevent land degradation. Additionally, the tool provided guidance on how to restore degraded lands through sustainable farming practices, allowing farmers to rehabilitate lands that were once considered unproductive.

The integration of these practices into SHFs’ farming systems resulted in increased soil health and productivity, thereby improving the resilience of their farms against climate impacts. As a result, the farmers were able to enhance their ability to adapt to climate change while also increasing their agricultural output.

Through continued use of tools like PACDR and ongoing support from local extension services, future projects can further improve SHFs' capacity to plan for and respond to climate challenges, ensuring their livelihoods remain secure.

- **Example Applications:** The integration of climate and weather information into farming practices proved to be highly effective for smallholder farmers (SHFs) within the project area. One notable example of this adaptation is the adjustment of planting times based on shifting rainfall patterns. By utilizing the climate information disseminated through the project’s workshops and local extension workers, farmers were able to better anticipate the onset of the rainy season and adjust their planting schedules accordingly. This allowed them to maximize crop yields and avoid the risks associated with planting too early or too late, which could result in crop failure due to unpredictable weather.

Additionally, farmers adopted contour farming as a key strategy to minimize soil erosion, particularly during heavy rains and floods. Contour farming, which involves planting along the natural contours of the land, helps slow down the movement of water, allowing it to soak into the soil instead of running off and causing erosion. This practice was especially crucial in areas prone to flooding or intense rainfall, as it prevented the loss of topsoil and maintained soil fertility. By creating small terraces or ridges along the contours, farmers were able to protect their land from further degradation while improving its ability to retain water, which is vital for crop growth during drier periods.

These adaptation strategies not only helped reduce the risks posed by erratic weather patterns but also contributed to the long-term sustainability of farming practices. As farmers became more attuned to the changing climate, they were able to proactively implement these methods, demonstrating a strong capacity for resilience and adaptation to the challenges posed by climate change.

**Income Diversification and Livelihood Strengthening:**

- The project facilitated IGAs like soap making, poultry farming, and vegetable gardening. Training sessions enhanced entrepreneurship and marketing skills.
- Despite successes, linking SHFs to markets remained a challenge, as many farmers were dependent on collection centers where they received low prices. Addressing market access in future projects is critical.

**Adoption of Adaptive Practices:**

- New practices like kitchen gardening, agroforestry, and soil conservation techniques were introduced. The promotion of compost manure over industrial fertilizers ensured soil health and sustainability.
- Challenges included resistance to change and limited land for new practices. These were addressed through follow-ups and farmer-to-farmer exchange visits (FAMO programs).

***Question 3*: Gender Participation and Climate Change Response**

**Gender-Responsive Adaptation Action Plans:**

- Women were encouraged to participate in adaptation planning through community dialogues and training sessions. Examples include women leading water management committees and IGAs, which boosted household resilience.
- **Community Empowerment:** The project placed a strong emphasis on **community empowerment** through capacity-building sessions aimed at fostering inclusive decision-making and promoting gender equality within households and the broader community. These sessions were designed to equip community members, particularly women, with the knowledge and skills to actively participate in decision-making processes related to agriculture, household resource management, and community development.

One of the key focuses was **reducing gender-based violence (GBV)**. The project recognized that gender inequality and violence were significant barriers to the well-being and empowerment of women, especially in rural areas. Through workshops and discussions, the project raised awareness about the negative impacts of GBV on families and communities, while also providing strategies for prevention and support. Community leaders and local influencers played a crucial role in disseminating these messages, fostering a safer and more supportive environment for women and children.

Additionally, the project worked to **promote harmony in households** by encouraging shared responsibilities and mutual respect between men and women. This was particularly important in agricultural households, where roles traditionally assigned to men and women can be restrictive. By encouraging joint decision-making on farm activities, resource allocation, and family planning, the project helped create more cooperative and equitable relationships. As a result, households were not only more cohesive but also better able to address challenges together, whether related to climate change, financial management, or day-to-day household tasks.

Through these efforts, the project contributed to strengthening social cohesion and enhancing the agency of marginalized groups, especially women, in community and family decisions, ultimately leading to more resilient and sustainable communities.

**Capacity Building for Gender and Rights:**

- The project also included **gender sensitization training**, which aimed to challenge and address traditional practices that often hindered women’s full participation in climate adaptation activities. Many of these traditional practices stem from long-standing gender roles that limit women’s access to resources, decision-making power, and economic opportunities. The training focused on raising awareness among both men and women about the importance of equal participation in climate change adaptation efforts, promoting gender equality in farming practices, and recognizing the vital role that women play in sustainable agricultural practices.

A key aspect of this training was the **promotion of women’s legal rights**. Many women in the project areas were unaware of their legal rights regarding land ownership, inheritance, and access to government programs. By educating women on these rights, the project empowered them to assert their entitlement to resources, protect their interests, and advocate for themselves within their households and communities.

In addition to legal rights, **financial literacy training** was provided to women to enhance their understanding of managing finances, budgeting, and making informed decisions about investments, savings, and loans. This training helped women improve their economic standing and contributed to their ability to invest in climate adaptation practices, such as drought-resistant crops or improved farming techniques. As a result, women gained greater control over their economic resources and had more leverage in decision-making both within their families and in the broader community.

To amplify the impact, the project emphasized the importance of **collaboration with other partners**. By working with local government agencies, civil society organizations, and private sector actors, the project was able to expand its reach and effectiveness. Collaborating with partners who specialize in gender equality, legal advocacy, and financial services helped create a more integrated approach to addressing women’s empowerment and climate adaptation. This multi-stakeholder approach not only strengthened the project’s outcomes but also fostered long-term partnerships that could continue supporting women’s empowerment and climate resilience efforts beyond the project’s duration.

Through these interventions, the project helped dismantle barriers to women’s participation in climate adaptation and equipped them with the tools, knowledge, and confidence to take active roles in shaping their future.

- **Impact of Dialogue Meetings:** These forums enhanced mutual understanding between genders, reducing conflicts and promoting shared responsibilities.

***Question 4*: School Feeding Programs**

**Role of the Project:**

- The project addressed malnutrition and school absenteeism by initiating **school feeding programs** in villages under pgms schools. Parents contributed maize for school meals, supported by community meetings organized by MFEC. Add process
- **Sustainability Measures:** School demonstration farms for maize and bananas were established, supported by training on improved agricultural techniques. Government collaboration further reinforced these efforts.

**Challenges:** Sustaining community contributions remains a concern, but ongoing mobilization and government support offer promising solutions.

**Recommendations:**

1. **Focus on Market Access:** Future projects should include comprehensive market linkage strategies to enable SHFs to sell their produce at fair prices without intermediaries. Value chain
2. **Address Gender Dynamics:** Engage gender experts to craft culturally sensitive approaches that promote inclusive development while respecting traditions.
3. **Strengthen Capacity Building:** Expand training on sustainable agricultural practices, IGAs, and financial literacy to ensure long-term benefits for SHFs.
4. **Enhance Climate Resilience:** Prioritize tools and frameworks like PACDR while integrating community feedback to refine climate adaptation strategies.
5. **Monitor Project Outcomes:** Implement follow-up mechanisms to track the sustainability of interventions, especially school feeding programs and gender-responsive action plans.

**Final Recommendations:** The MFEC project has demonstrated a comprehensive approach to sustainable livelihood development, integrating agricultural, social, and environmental interventions. However, emphasis on market linkages, gender-sensitive strategies, and long-term sustainability mechanisms would ensure the project’s legacy end
