# Supplementary material for: Evaluating Effectiveness of Sustainable Livelihood Development in Rural Communities along Mara River Basin, Tanzania: What Works, What Doesn’t Work, and Why?
Source: PLoS One. 2026 Jun 11;21(6):e0351252. doi: 10.1371/journal.pone.0351252 (PMC13258000; doi:10.1371/journal.pone.0351252)
Supplement: Attachment — Submitted filename: PONE-D-25-05903.pdf [file pone.0351252.s005.pdf]

PLOS ONE

Evaluating Effectiveness of Sustainable Livelihood Development in Rural Communities along Mara River Basin, Tanzania: What Works, What Doesn’t Work, and Why?

--Manuscript Draft--

**Manuscript Number:**	PONE-D-25-05903
**Article Type:**	Research Article
**Full Title:**	Evaluating Effectiveness of Sustainable Livelihood Development in Rural Communities along Mara River Basin, Tanzania: What Works, What Doesn’t Work, and Why?
**Short Title:**	Effectiveness of Sustainable Livelihood Development in Rural Communities Along Mara River Basin, Tanzania
**Corresponding Author:**	Edwin Estomii Ngowi, Ph.D.<br>Sokoine University of Agriculture<br>Morogoro, Tanzania, TANZANIA, UNITED REPUBLIC OF
**Keywords:**	Sustainable livelihoods; climate adaptation; income diversification; gender equality; rural development; smallholder farmers (SHFs); Mara River basin; Tanzania
**Abstract:**	The “Sustainable Livelihood Development of Rural Communities in Low Land Along the Mara River Basin” project, implemented by Mogabiri Farm Extension Centre (MFEC) in Tarime District, Tanzania, seeks to address persistent socio-economic challenges in rural communities by promoting climate adaptation, income diversification, and gender equality. Despite its ambitious objectives, the project operates within a complex socio-economic and cultural environment that can undermine its intended outcomes. Evaluating such projects requires a critical examination of both their successes and the barriers that persist. Using a mixed-methods approach, this evaluation combined quantitative data from 265 smallholder farmers (SHFs) and qualitative insights from focus group discussions (FGDs), key informant interviews (KIIs), and document reviews. In terms of “What Works?”, findings indicate that 75% of SHFs adopted climate-resilient practices ($p<0.05$), and 90% of communities implemented gender-responsive action plans ($p<0.01$), with 68% of households engaging in income diversification activities such as poultry farming and beekeeping. However, the question of “What Doesn’t Work?” revealed notable shortcomings, including limited market access ($p=0.03$) and cultural barriers restricting full gender participation ($p=0.04$). The persistence of these challenges, despite project successes, underscores the need for a deeper investigation into structural and contextual factors that constrain the effectiveness of such initiatives. Understanding “Why?” these barriers remain highlights the interplay of systemic market integration challenges and cultural resistance to gender equity. While the project has made commendable progress, addressing these underlying issues is crucial for achieving long-term success. Strengthening market linkages, expanding gender-sensitive interventions, and fostering sustainability through community ownership and local partnerships are recommended to enhance scalability and impact.
**Order of Authors:**	Edwin Estomii Ngowi, Ph.D.
	Angela M. Jesse, Ph.D.
**Additional Information:**	
**Question**	**Response**
**Financial Disclosure**<br><br>Enter a financial disclosure statement that describes the sources of funding for the work included in this submission. Review the submission guidelines for detailed requirements. View published research articles from *PLOS ONE* for specific examples.	The author(s) received no specific funding for this work.

This statement is required for submission and **will appear in the published article** if the submission is accepted. Please make sure it is accurate.<br><br>**Funded studies**<br>Enter a statement with the following details:<br>• Initials of the authors who received each award<br>• Grant numbers awarded to each author<br>• The full name of each funder<br>• URL of each funder website<br>• Did the sponsors or funders play any role in the study design, data collection and analysis, decision to publish, or preparation of the manuscript?<br><br>Did you receive funding for this work?	
**Competing Interests**<br><br>Use the instructions below to enter a competing interest statement for this submission. On behalf of all authors, disclose any competing interests that could be perceived to bias this work—acknowledging all financial support and any other relevant financial or non-financial competing interests.<br><br>This statement is required for submission and **will appear in the published article** if the submission is accepted. Please make sure it is accurate and that any funding sources listed in your Funding Information later in the submission form are also declared in your Financial Disclosure statement.<br><br>View published research articles from *PLOS ONE* for specific examples.	The authors have declared that no competing interests exist.

**NO authors have competing interests**<br><br>Enter: *The authors have declared that no competing interests exist.*<br><br>**Authors with competing interests**<br><br>Enter competing interest details beginning with this statement:<br><br>*I have read the journal's policy and the authors of this manuscript have the following competing interests: [insert competing interests here]*<br><br>* typeset	
**Ethics Statement**<br><br>Enter an ethics statement for this submission. This statement is required if the study involved:<br><br>• Human participants<br>• Human specimens or tissue<br>• Vertebrate animals or cephalopods<br>• Vertebrate embryos or tissues<br>• Field research<br><br>Write "N/A" if the submission does not require an ethics statement.<br><br>General guidance is provided below. Consult the submission guidelines for detailed instructions. **Make sure that all information entered here is included in the Methods section of the manuscript.**	ETHICAL APPROVAL AND INFORMED CONSENT:<br>This study received ethical approval from the Sokoine University of Agriculture (SUA), granted by the Directorate of Postgraduate Studies, Research, Technology Transfer, and Consultancy. The approval reference number for the Institutional Review Board (IRB) was SUA/DRPRTC/PPM/D/2022/0010. Following this approval, a research permit was sought from the President's Office of Regional Administration and Local Government (PO-RALG) to allow the study to be conducted within local government authorities. The permit was officially granted under the registration number AB.307/323/01/175, authorizing the researcher to carry out the study in the selected areas.<br><br>INFORMED CONSENT:<br>This study ensured that consent was informed by providing all participants with a detailed explanation of the study's objectives, procedures, potential risks, and benefits before their participation. The study obtained written informed consent from all literate participants who voluntarily agreed to take part. For illiterate participants, verbal consent was obtained in the presence of an impartial witness, who then signed the consent form on their behalf to document and verify their agreement.<br><br>CONSENT FOR MINORS:<br>This study did not involve minors. However, in cases where individuals under the age of 18 participated, parental or guardian consent was obtained prior to their involvement, ensuring compliance with ethical guidelines.<br><br>CONFIDENTIALITY AND ANONYMITY:<br>To protect participant privacy, all collected data were anonymized and securely stored. Identifiable personal information was removed from reports and publications. Additionally, data access was restricted to authorized research team members only, ensuring that sensitive information remained confidential throughout the research process.<br><br>RISK MINIMIZATION:<br>The study posed minimal risk to participants, as interviews and discussions were conducted in culturally appropriate and comfortable settings. Participants were fully informed of their right to withdraw from the study at any time without facing any

**Format for specific study types**<br><br>**Human Subject Research (involving human participants and/or tissue)**<br>• Give the name of the institutional review board or ethics committee that approved the study<br>• Include the approval number and/or a statement indicating approval of this research<br>• Indicate the form of consent obtained (written/oral) or the reason that consent was not obtained (e.g. the data were analyzed anonymously)<br><br>**Animal Research (involving vertebrate animals, embryos or tissues)**<br>• Provide the name of the Institutional Animal Care and Use Committee (IACUC) or other relevant ethics board that reviewed the study protocol, and indicate whether they approved this research or granted a formal waiver of ethical approval<br>• Include an approval number if one was obtained<br>• If the study involved *non-human primates*, add *additional details* about animal welfare and steps taken to ameliorate suffering<br>• If anesthesia, euthanasia, or any kind of animal sacrifice is part of the study, include briefly which substances and/or methods were applied<br><br>**Field Research**<br><br>Include the following details if this study involves the collection of plant, animal, or other materials from a natural setting:<br>• Field permit number<br>• Name of the institution or relevant body that granted permission	negative consequences. Any signs of discomfort or reluctance were respected to uphold ethical research standards.<br><br>ETHICS WAIVER:<br>No ethics waivers were requested or granted for this study. The study adhered to ethical requirements by securing approvals from the Sokoine University of Agriculture (SUA) Institutional Review Board (IRB) under reference number SUA/DRPRTC/PPM/D/2022/0010 and obtaining an official research permit from the President's Office of Regional Administration and Local Government (PO-RALG) under registration number AB.307/323/01/175. Additionally, consultations with community leaders and local authorities were conducted to ensure ethical and culturally appropriate engagement with participants.<br>Therefore, by adhering to these ethical principles, this study maintained high research integrity while safeguarding participant rights and well-being. Ensuring informed consent, confidentiality, and risk minimization not only upheld the ethical standards set by Sokoine University of Agriculture (SUA) but also fostered trust between the researchers and the study participants. These measures contributed to the credibility and reliability of the study findings, as participants felt safe to provide honest and accurate responses. Additionally, by securing necessary approvals from relevant authorities and engaging local stakeholders, the study aligned with both institutional and national research guidelines, reinforcing its ethical rigor and social responsibility.
**Data Availability**<br><br>Authors are required to make all data underlying the findings described fully available, without restriction, and from the time of publication. PLOS allows rare exceptions to address legal and ethical concerns. See the PLOS Data Policy and FAQ for detailed information.	Yes - all data are fully available without restriction

A Data Availability Statement describing where the data can be found is required at submission. Your answers to this question constitute the Data Availability Statement and **will be published in the article**, if accepted.<br><br>**Important:** Stating 'data available on request from the author' is not sufficient. If your data are only available upon request, select 'No' for the first question and explain your exceptional situation in the text box.<br><br>Do the authors confirm that all data underlying the findings described in their manuscript are fully available without restriction?	
**Describe where the data may be found in full sentences. If you are copying our sample text, replace any instances of XXX with the appropriate details.**<br><br>• If the data are **held or will be held in a public repository**, include URLs, accession numbers or DOIs. If this information will only be available after acceptance, indicate this by ticking the box below. For example: *All XXX files are available from the XXX database (accession number(s) XXX, XXX.)*.<br>• If the data are all contained **within the manuscript and/or Supporting Information files**, enter the following: *All relevant data are within the manuscript and its Supporting Information files.*<br>• If neither of these applies but you are able to provide **details of access elsewhere**, with or without limitations, please do so. For example:<br><br>*Data cannot be shared publicly because of [XXX]. Data are available from the XXX Institutional Data Access / Ethics Committee (contact via XXX) for researchers who meet the criteria for access to confidential data.*<br><br>*The data underlying the results presented in the study are available from (include the name of the third party*	All relevant data are within the manuscript and its Supporting Information files.

and contact information or URL).<br>• This text is appropriate if the data are owned by a third party and authors do not have permission to share the data.<br><br>* typeset	
Additional data availability information:	Tick here if your circumstances are not covered by the questions above and you need the journal’s help to make your data available.

Manuscript Click here to access/download;Manuscript;Manuscript Final.docx

Evaluating Effectiveness of Sustainable Livelihood Development in Rural Communities along Mara River Basin, Tanzania: *What Works, What Doesn't Work,* and *Why?*

Edwin E. Ngowi[1] and Angela M. Jesse[2]

[1]*Department of Development & Strategic Studies, Sokoine University of Agriculture, Morogoro, Tanzania. Email: edwin.ngowi@sua.ac.tz*
[2]*Department of Development & Strategic Studies, Sokoine University of Agriculture, Morogoro, Tanzania. Email: angela.jesse@sua.ac.tz*

Abstract
The "*Sustainable Livelihood Development of Rural Communities in Low Land Along the Mara River Basin*" project, implemented by Mogabiri Farm Extension Centre (MFEC) in Tarime District, Tanzania, seeks to address persistent socio-economic challenges in rural communities by promoting climate adaptation, income diversification, and gender equality. Despite its ambitious objectives, the project operates within a complex socio-economic and cultural environment that can undermine its intended outcomes. Evaluating such projects requires a critical examination of both their successes and the barriers that persist. Using a mixed-methods approach, this evaluation combined quantitative data from 265 smallholder farmers (SHFs) and qualitative insights from focus group discussions (FGDs), key informant interviews (KIIs), and document reviews. In terms of "*What Works?*", findings indicate that 75% of SHFs adopted climate-resilient practices ($p<0.05$), and 90% of communities implemented gender-responsive action plans ($p<0.01$), with 68% of households engaging in income diversification activities such as poultry farming and beekeeping. However, the question of "*What Doesn't Work?*" revealed notable shortcomings, including limited market access ($p=0.03$) and cultural barriers restricting full gender participation ($p=0.04$). The persistence of these challenges, despite project successes, underscores the need for a deeper investigation into structural and contextual factors that constrain the effectiveness of such initiatives. Understanding "*Why?*" these barriers remain highlights the interplay of systemic market integration challenges and cultural resistance to gender equity. While the project has made commendable progress, addressing these underlying issues is crucial for achieving long-term success. Strengthening market linkages, expanding gender-sensitive interventions, and fostering sustainability through community ownership and local partnerships are recommended to enhance scalability and impact.

Keywords: Sustainable livelihoods, climate adaptation, income diversification, gender equality, rural development, smallholder farmers (SHFs), Mara River basin, Tanzania

1. Introduction

Sustainable livelihood development is a key strategy for addressing poverty, food insecurity, and vulnerability in rural communities, particularly in regions prone to climate variability and environmental degradation. The Mara River Basin, a vital transboundary resource between Tanzania and Kenya, supports the livelihoods of millions through agriculture, livestock keeping, and fishing activities. However, the basin faces significant challenges due to deforestation, land degradation, and over-reliance on natural resources, exacerbated by climate

change impacts (Ogutu et al., 2020; WWF, 2021). These issues threaten not only the ecological integrity of the basin but also the socio-economic well-being of communities that depend on its resources (Mkonda & He, 2018).

Efforts to enhance sustainable livelihoods in rural contexts are essential for achieving resilience, promoting gender equality, and fostering inclusive development. Sustainable livelihoods are defined as the capabilities, assets, and activities required for a means of living, particularly under conditions of vulnerability (Chambers & Conway, 1992). Effective interventions must address multidimensional challenges, including climate adaptation to mitigate environmental shocks, income diversification to reduce economic dependency, and the empowerment of marginalized groups, especially women, to ensure equitable access to resources and opportunities (Ellis, 2000; Scoones, 1998). Addressing these dimensions holistically can lead to more robust and equitable rural development outcomes while contributing to broader Sustainable Development Goals (SDGs), such as gender equality (Goal 5) and climate action (Goal 13) (United Nations, 2015).

Existing studies in the Mara River Basin have documented the success of ecosystem-based approaches in mitigating environmental degradation and promoting resilience among smallholder farmers (Gichuki et al., 2019; Ogutu et al., 2020). These initiatives emphasize the importance of integrating scientific innovations with indigenous knowledge to support sustainable agricultural practices. Specifically, agroecological interventions such as soil and water conservation techniques, agroforestry, and integrated pest management have demonstrated potential in enhancing farm productivity while preserving the environment (Mwangi et al., 2021; Kariuki et al., 2022). Despite these advancements, significant gaps persist in understanding the socio-economic and cultural barriers that hinder the widespread adoption of such practices. Structural limitations such as inadequate access to financial resources, lack of extension services, and poor market linkages often discourage farmers from implementing sustainable methods (Mutunga et al., 2021). Furthermore, deeply ingrained traditional beliefs, limited land ownership rights, especially among women, and varying levels of awareness about climate adaptation strategies continue to pose challenges (Ndungu & Nyangena, 2020; Ochieng et al., 2023). Addressing these barriers requires a multi-faceted approach that includes participatory research, targeted policy reforms, and enhanced collaboration between local communities, government agencies, and non-governmental organizations to facilitate knowledge exchange and increase the adoption of sustainable agricultural practices in the region (Njoroge et al., 2023; Kiptoo & Wambua, 2024).

For instance, limited access to markets, cultural resistance to gender equality, and insufficient capacity-building initiatives have been identified as persistent challenges in rural development programs (Njuki et al., 2014; FAO, 2022). Moreover, while there is ample evidence of successful climate adaptation practices, the sustainability and scalability of these interventions remain uncertain, particularly in resource-constrained settings (Mkonda & He, 2018). Addressing these gaps requires a holistic evaluation of livelihood development projects to identify "*what works*," "*what doesn't work*," and the underlying reasons for success or failure.

This paper evaluates the effectiveness of the "*Sustainable Livelihood Development of Rural Communities in Low Land Along the Mara River Basin*" project, implemented by Mogabiri Farm Extension Centre (MFEC) in Tarime District, Tanzania. This initiative sought to enhance the socio-economic well-being of rural communities by promoting climate adaptation, income diversification, and gender equality, targeting 850 smallholder farmers across ten villages. The primary objective of this study was to assess the project's impact by identifying successful interventions, addressing persistent challenges, and analyzing the underlying factors influencing these outcomes. The evaluation adopts a holistic approach to highlight key achievements, such as the adoption of climate-resilient practices, increased gender equity, and the uptake of diversified income-generating activities, while also addressing systemic issues like market access and cultural barriers. Therefore, by drawing on the sustainable livelihood's framework (Chambers & Conway, 1992; Scoones, 1998), the study provides actionable recommendations for enhancing the scalability and sustainability of rural livelihood development initiatives. These findings are particularly relevant for informing future interventions aimed at fostering resilience and inclusive development in rural communities (Ellis, 2000; United Nations, 2015).

2. The Theoretical Framework

The theoretical framework for evaluating the effectiveness of the "*Sustainable Livelihood Development of Rural Communities in Low Land Along the Mara River Basin*" project is grounded in the *Sustainable Livelihoods Framework (SLF).* Developed by Chambers and Conway (1992) and expanded by Scoones (1998), the SLF provides a comprehensive lens to understand and address the multidimensional aspects of rural poverty and development. This framework emphasizes the interconnections among five key components: *livelihood assets, vulnerability context, transforming structures and processes, livelihood strategies, and livelihood outcomes.*

i. Livelihood Assets

At the heart of the SLF are the five types of livelihood assets; *human, natural, social, physical, and financial capital;* which determine the capacity of individuals or communities to pursue sustainable livelihoods. In this project, interventions such as capacity-building initiatives and income diversification (for instance, poultry farming and beekeeping) enhanced human and financial capital, while gender-responsive action plans strengthened social capital.

ii. Vulnerability Context

The SLF acknowledges that rural communities are vulnerable to external shocks and trends, such as climate change and economic instability. In the Mara River Basin, climate variability exacerbates resource degradation and disrupts traditional livelihood systems (Ogutu et al., 2020). As such, by promoting climate-resilient practices, the project aimed to reduce vulnerability and enhance adaptive capacity.

iii. Transforming Structures and Processes

Institutions, policies, and cultural norms influence access to resources and opportunities. The project engaged local governance structures and community groups to implement gender-responsive action plans. However, cultural resistance to gender equity and market access limitations persisted as barriers, reflecting the need for transformative changes in institutional and social norms (FAO, 2022; Njuki et al., 2014).

iv. Livelihood Strategies

Sustainable livelihood strategies involve choices that individuals and communities make to achieve their goals. The project supported diversification into activities such as poultry farming and beekeeping, which reduced dependency on climate-sensitive agriculture. Henceforth, by aligning these strategies with community priorities, the project demonstrated significant improvements in household incomes and resilience.

v. Livelihood Outcomes

Livelihood outcomes include improved well-being, reduced vulnerability, and enhanced resource sustainability. The findings revealed substantial progress, with 75% of smallholder farmers adopting climate-resilient practices ($p < 0.05$) and 90% of communities implementing gender-responsive action plans ($p < 0.01$). However, systemic challenges, such as limited market integration and cultural barriers, constrained the full realization of outcomes.

vi. Integration of Gender and Climate Dimensions

The SLF's flexibility allows for the integration of cross-cutting themes, such as gender equality and climate adaptation. Gender is critical, as equitable access to resources and opportunities strengthens the socio-economic fabric of communities. Likewise, climate adaptation practices ensure the sustainability of natural resources critical for rural livelihoods (Mkonda & He, 2018; United Nations, 2015).

This theoretical framework highlights the interconnectedness of assets, external factors, and institutional processes in shaping livelihood outcomes. It also underscores the importance of addressing systemic barriers and fostering community ownership to enhance the scalability and sustainability of development initiatives.

3. Methodology

3.1 Study Area Description

The study was conducted in Mara River Basin (MRB), which spans across the northern regions of Tanzania and southwestern Kenya. In Tanzania, the MRB covers the districts of Tarime, Rorya, and Serengeti in Mara Region. The basin is ecologically significant, encompassing diverse ecosystems such as forests, grasslands, and wetlands. It is home to vital wildlife habitats, including parts of the Serengeti National Park, which is globally recognized for its biodiversity (McClain et al., 2014). The basin also serves as a critical water source for local communities, livestock, and agricultural activities, making it central to rural livelihoods. The recruitment period of SHFs for this study began on 10/11/2024 and concluded on 23/12/2024.

The selection of this area is justified by its socio-ecological importance and the challenges it faces, including environmental degradation, poverty, and unsustainable livelihood practices (Kairu et al., 2018). The basin has been the focus of various sustainable livelihood initiatives aimed at addressing these issues. However, the effectiveness of these interventions remains underexplored, necessitating a localized and in-depth evaluation to inform policy and practice.

The climate in the MRB is characterized by bimodal rainfall patterns, with short rains occurring between October and December and long rains from March to May. Annual rainfall ranges from 1,000 mm in highland areas to 700 mm in lowland areas, which influences agricultural

productivity and water availability (Chauhan & Nayak, 2020). The rural communities in the basin predominantly rely on subsistence farming, livestock keeping, and fishing, making them vulnerable to environmental and economic shocks.

The "*Sustainable Livelihood Development of Rural Communities Along the Mara River Basin*" project, initiated in July 2022 in Tarime District, Mara Region, Tanzania. The district, reliant on the Mara River for agriculture, livestock rearing, and fishing, faces challenges like land degradation, deforestation, and poverty, necessitating sustainable interventions (McClain et al., 2014; Kairu et al., 2018). Covering 10 villages across five wards, that is, Kembwi, Mtana (Manga Ward); Kewamamba, Nyagisya, Nkerege (Kyore Ward); Surubu (Komaswa Ward); Keisaka (Kibasuka Ward); and Turugeti, Kitenga, Kwisarara (Bumera Ward), the project targets 850 smallholder farmers engaged in resource-dependent livelihoods, emphasizing their vulnerability to environmental degradation and involvement in past development efforts.

Henceforth, the justification for selecting this study area is rooted in its ecological and socio-economic importance. The MRB is not only a critical water source for communities and wildlife but also a hotspot for sustainable development interventions. The study area allowed for an evaluation of livelihood projects in diverse ecological and socio-economic settings, thereby providing insights into *what works*, *what doesn't*, and *why* in sustainable livelihood development initiatives.

3.2 Methodological Design and Approach

This study employed a mixed-methods approach designed to address the study's core research questions- "*What works, what doesn't,* and *why*?"-by collecting and analyzing both quantitative and qualitative data. A mixed-methods evaluation design was adopted to triangulate findings from diverse data sources and methodologies. Quantitative methods provided statistical insights into the project's measurable impacts, while qualitative methods offered context and depth to understand the underlying dynamics. This approach enhances reliability and validity, as highlighted by Creswell and Plano Clark (2018). The evaluation focused on three thematic pillars: climate adaptation, income diversification, and gender participation.

3.3 Data Collection Methods

A combination of qualitative and quantitative methods was employed to collect data for this evaluation. A detailed document review was conducted, analyzing project design documents, baseline surveys, operational plans, reports, training manuals, and audit records. This approach provided essential benchmarks for assessing outcomes and identifying lessons learned, consistent with the effectiveness of document reviews in retrospective evaluations (Bowen, 2009). The recruitment period for this study began on 10/11/2024 and concluded on 23/12/2024. This period allowed for the identification, selection, and enrolment of study participants, ensuring adequate representation across the 10 project villages. Structured questionnaire-based surveys were administered to 265 smallholder farmers (SHFs), selected through stratified random sampling to ensure proportional representation across the 10 project villages. The surveys captured demographic characteristics, adoption of climate-resilient practices, participation in income-generating activities, and perceptions of gender inclusion.

The sample size, calculated using Cochran's (1977) formula, achieved a 95% confidence level with a 5% margin of error. A widely used formula for sample size determination in finite populations is:

$$n = \frac{NZ^2p(1-p)}{E^2(N-1) + Z^2p(1-p)}$$

Where:

- n = Sample size
- The total number of smallholder farmers in the study area is N = 850.
- Confidence Level: 95%
- Margin of Error (E): 5% (0.05)
- Standard Z-score for a 95% confidence level: Z = 1.96
- p = Estimated proportion of the population (usually 0.5 when unknown, as it maximizes variability)

$$n = \frac{850 \times (1.96)^2 \times 0.5 \times (1-0.5)}{(0.05)^2 \times (850-1) + (1.96)^2 \times 0.5 \times (1-0.5)}$$

The calculated sample size is approximately 265 smallholder farmers. [illegible]s means that to ensure a representative and statistically valid survey, at least 265 respondents should be selected from the total population of 850 farmers.

Six Focus Group Discussions (FGDs) were conducted with 6–15 participants in each group, including separate sessions for Farmer Motivators, men, women, and youth to ensure inclusivity. FGDs explored participants' experiences, challenges, and perceptions of the project's effectiveness, facilitated by experienced moderators to promote open dialogue. Key Informant Interviews (KIIs) with stakeholders, including extension officers, district officials, project staff, and community leaders, provided expert insights into the project's design and outcomes, aligning with the contextual depth emphasized by Yin (2017). Observational field visits validated self-reported data and assessed the physical implementation of interventions, such as climate adaptation practices, the functionality of VICOBA, and women-led income-generating activities. These observations offered real-time perspectives on outcomes and challenges, enriching the evaluation findings.

3.4 Data Analysis

3.4.1 Quantitative Data Analysis

Quantitative data were analyzed using IBM SPSS Statistics software, version 20. Descriptive statistics (frequencies, percentages, means) summarized key variables, while cross-tabulations explored relationships between participation levels and project outcomes. Inferential statistics, including chi-square tests, ANOVA test, and t-tests, evaluated the significance of observed differences (Field, 2018). Results were visualized through graphs and tables for clarity.

3.4.2 Qualitative Data Analysis

Qualitative data from FGDs and KIIs were analyzed using thematic analysis. This involved coding transcripts to identify recurring themes and patterns related to climate adaptation,

income diversification, and gender participation. Thematic analysis provided a rich narrative complementing quantitative findings, as described by Braun and Clarke (2006).

3.5 Ethical Approval and Informed Consent

This study received ethical approval from the Sokoine University of Agriculture (SUA), granted by the Directorate of Postgraduate Studies, Research, Technology Transfer, and Consultancy. The approval reference number for the Institutional Review Board (IRB) was *SUA/DRPRTC/PPM/D/2022/0010*. Following this approval, a research permit was sought from the President's Office of Regional Administration and Local Government (PO-RALG) to allow the study to be conducted within local government authorities. The permit was officially granted under the registration number *AB.307/323/01/175*, authorizing the researcher to carry out the study in the selected areas.

3.5.1 Informed Consent

This study ensured that consent was informed by providing all participants with a detailed explanation of the study's objectives, procedures, potential risks, and benefits before their participation. The study obtained written informed consent from all literate participants who voluntarily agreed to take part. For illiterate participants, verbal consent was obtained in the presence of an impartial witness, who then signed the consent form on their behalf to document and verify their agreement.

3.5.2 Consent for Minors

This study did not involve minors. However, in cases where individuals under the age of 18 participated, parental or guardian consent was obtained prior to their involvement, ensuring compliance with ethical guidelines.

3.5.3 Confidentiality and Anonymity

To protect participant privacy, all collected data were anonymized and securely stored. Identifiable personal information was removed from reports and publications. Additionally, data access was restricted to authorized research team members only, ensuring that sensitive information remained confidential throughout the research process.

3.5.4 Risk Minimization

The study posed minimal risk to participants, as interviews and discussions were conducted in culturally appropriate and comfortable settings. Participants were fully informed of their right to withdraw from the study at any time without facing any negative consequences. Any signs of discomfort or reluctance were respected to uphold ethical research standards.

3.5.5 Ethics Waiver

No ethics waivers were requested or granted for this study. The study adhered to ethical requirements by securing approvals from the Sokoine University of Agriculture (SUA) Institutional Review Board (IRB) under reference number *SUA/DRPRTC/PPM/D/2022/0010* and obtaining an official research permit from the President's Office of Regional Administration and Local Government (PO-RALG) under registration number *AB.307/323/01/175*. Additionally, consultations with community leaders and local authorities were conducted to ensure ethical and culturally appropriate engagement with participants.

Therefore, by adhering to these ethical principles, this study maintained high research integrity while safeguarding participant rights and well-being. Ensuring informed consent,

confidentiality, and risk minimization not only upheld the ethical standards set by Sokoine University of Agriculture (SUA) but also fostered trust between the researchers and the study participants. These measures contributed to the credibility and reliability of the study findings, as participants felt safe to provide honest and accurate responses. Additionally, by securing necessary approvals from relevant authorities and engaging local stakeholders, the study aligned with both institutional and national research guidelines, reinforcing its ethical rigor and social responsibility.

4 Results

4.1 Distribution of Smallholder Farmers (SHFs) by Residence and Crops Cultivated

The residence distribution of SHFs across wards and villages highlights varying levels of participation, accessibility, and project engagement (Figure 1). At the ward level, Bumera had the highest proportion of SHFs (53.2%), followed by Kiore (24.2%) and Kibasuka (10.2%). Manga and Komaswa accounted for the lowest participation rates, at 7.2% and 5.3%, respectively.

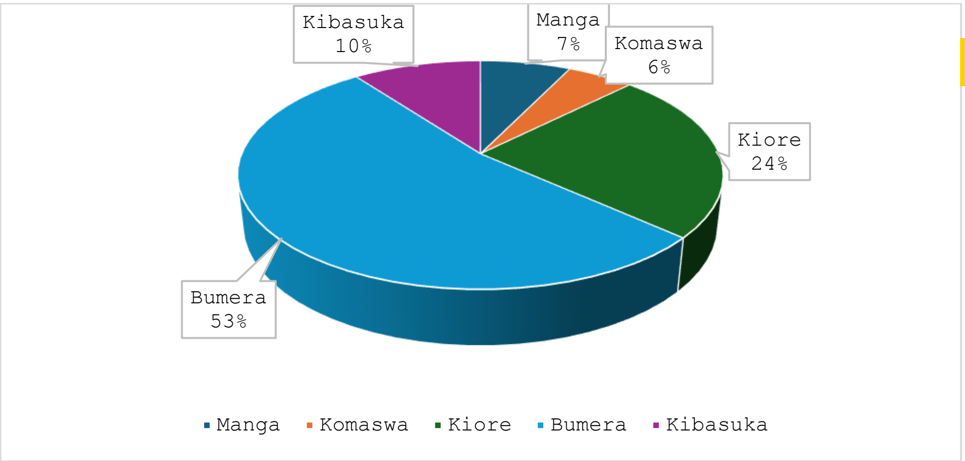

Figure 1: Ward of Residence of SHFs

This variation in participation reflects logistical challenges, differing engagement levels, and potential socio-economic factors affecting community involvement. In Figure 2, villages like Kitenga recorded the highest share of SHFs (24.9%), followed by Turugeti and Kwisarara (14% each). In contrast, Surubu and Nyagisya had the lowest proportions, at 5.3% and 5.7%, respectively. The prominence of Kitenga can be linked to improved accessibility, stronger project outreach, and higher awareness levels among farmers.

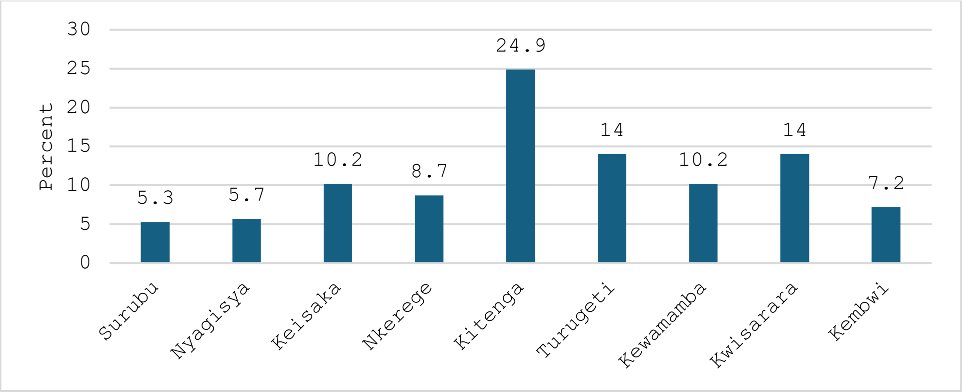

Figure 2: Village of Residence of SHFs

A Chi-square test of independence was conducted to assess whether there was a statistically significant association between the ward of residence and the participation levels of smallholder farmers (SHFs) in the study area. This test was chosen to determine if the distribution of participation levels varied across different wards, indicating potential location-based influences on engagement in agricultural activities. The analysis examined observed and expected frequencies to identify any significant deviations that could suggest underlying socio-economic, infrastructural, or environmental factors affecting participation. The results of this analysis are presented in Table 1, providing insights into the relationship between geographical location and farmer involvement in the studied agricultural initiatives.

Table 1: SHFs Distribution by Residence

Variable	Chi-Square Value	Degrees of Freedom (df)	p-value	Significance
Ward Distribution	27.34	4	< 0.001	Significant

The analysis reveals a statistically significant relationship (p<0.001) between ward residence and SHF participation. Wards with higher accessibility and project engagement, such as Bumera, reported higher participation rates.

Furthermore, crop cultivation practices in the MFEC project villages exhibited a significant degree of diversity, indicating a strategic focus on food security, income stability, and climate adaptation. The dominant crops were maize and cassava, often intercropped with other crops like millet, beans, sweet potatoes, and groundnuts (Figure 3). Key crop combinations included: Maize with cassava, millet, and beans (4.2%); Maize with sweet potatoes (4.6%); Cassava with maize, beans, and sweet potatoes (3%); and Cassava with groundnuts, millet, or beans (1.5% each).

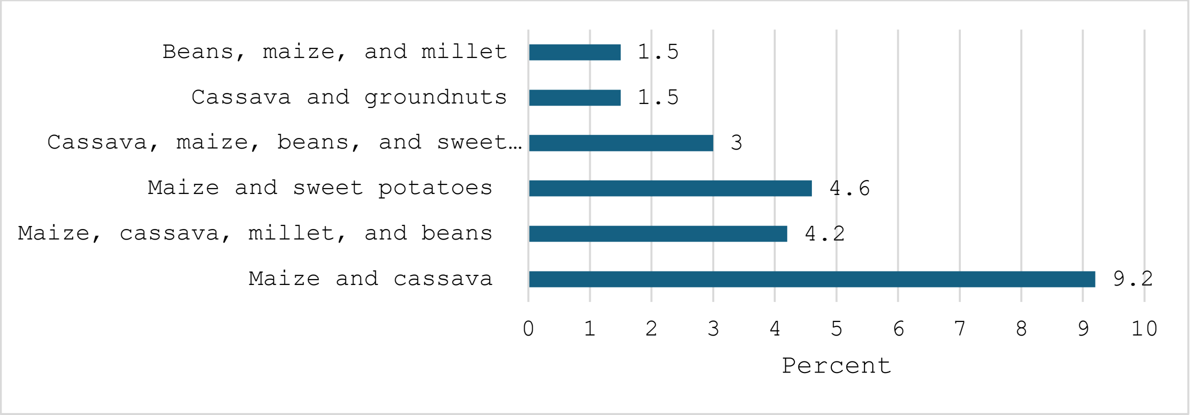

Figure 3: Crops Cultivated in the MFEC Project Villages

The predominance of maize and cassava reflects their adaptability to local agro-ecological conditions and their roles in ensuring food security. Cassava, recognized for its drought resilience, was a key crop in villages prone to erratic rainfall.

An Analysis of Variance (ANOVA) test was conducted to determine whether there were significant differences in crop yields across the sampled villages. This statistical test was chosen to compare mean yield variations among multiple villages and assess whether the observed differences were due to random chance or underlying factors such as soil fertility, farming practices, access to inputs, or climatic conditions. As such, by analyzing variance within and between villages, the test provided insights into potential disparities in agricultural productivity. The results of the ANOVA test are summarized in Table 2, highlighting key differences in yield distributions across the study locations.

Table 2: Crop Yield Analysis

**Source**	**Sum of Squares**	**df**	**Mean Square**	**F-value**	**p-value**
Between Villages	482.76	8	60.35	5.92	< 0.01
Within Villages	1,204.89	216	5.58		
**Total**	**1,687.65**	**224**			

The ANOVA results indicate statistically significant differences in crop yields among villages ($p<0.01$), suggesting that factors such as resource availability, farming techniques, and local environmental conditions contribute to yield variations. Specifically, villages with more targeted agricultural training programs, improved extension services, and better access to farming inputs, such as Kitenga and Turugeti, reported significantly higher yields compared to other villages. These findings underscore the crucial role of capacity-building initiatives and equitable resource distribution in enhancing agricultural productivity. Addressing disparities in access to improved seeds, fertilizers, irrigation infrastructure, and extension support could help bridge the yield gap and promote sustainable farming practices across all villages.

4.2 Project Relevance and Awareness

MFEC implemented a series of targeted interventions and training programs to address key social and agricultural challenges faced by SHFs in the project villages. These efforts specifically tackled persistent issues such as low agricultural productivity, poor soil fertility, climate change impacts, and socio-economic inequalities. The interventions were designed in alignment with community priorities identified through participatory assessments, focus group discussions (FGDs), and consultations with local leadership. As detailed in Table 3, these participatory approaches ensured that interventions were tailored to the specific needs of each village, enhancing their effectiveness and long-term sustainability.

Table 3 provides a summary of the challenges addressed, the interventions implemented, the agricultural practices adopted, and the resulting outcomes. For instance, training sessions on sustainable farming practices equipped farmers with knowledge on soil conservation, crop rotation, and organic fertilization, leading to noticeable improvements in soil fertility and increased crop yields. Similarly, the promotion of climate-smart agricultural techniques, such as drought-resistant seed varieties and water conservation methods, significantly enhanced

SHF resilience to climatic shocks. Additionally, initiatives aimed at addressing socio-economic inequalities, such as access to credit facilities and market linkages, contributed to improved financial stability and increased household incomes among farmers. These collective efforts highlight the importance of integrated, community-driven interventions in fostering sustainable agricultural development and improving rural livelihoods.

Table 3: Summary of the Challenges Addressed, MFEC Interventions, Adopted Practices, and Impact/Outcomes Reported during FGDs

Challenge	MFEC Intervention	Adopted Practice	Reported Impact/Outcomes
Low Productivity	Training in Sustainable Farming Practices	Organic manure preparation; integrated farming methods; crop rotation; intercropping; and contour farming	Improved soil fertility and higher crop yields
Poor Soil Fertility	Training in the Use of organic fertilizers and conservation	Organic manure preparation	Improved soil health and fertility
Climate Change Impacts	Climate-smart agriculture	Short-season crops, mulching	Increased resilience to climatic shocks
Soil Erosion and Deforestation	Environmental conservation	Agroforestry, tree planting	Reduced land degradation and improved resource management
Limited Income Diversification	Livelihood diversification	Beekeeping, poultry farming	Additional household income and financial stability
Lack of Access to Credit	Financial literacy training	Formation of VICOBA and FAMOs	Improved access to financial resources and strengthened community cohesion and economic empowerment
High Post-Harvest Losses	Training in Seed Storage Techniques	Preservation of native seeds; use of improved storage facilities	Reduced crop losses and better food security
Patriarchal norms	Gender equality training	Formation of women groups, leadership mentorship	Increased women's leadership roles
Resource ownership conflicts	Community consultations	Joint family land-use agreements	Improved family decision-making
Insufficient Training in Sustainable Practices	Practical Demonstrations via "*demonstration farm*"	Compost preparation, proper planting methods (row planting, accurate spacing)	Enhanced knowledge application, increased farm productivity

The qualitative information from Table 3 is supported by the data from Figure 4 in which respondents mentioned MFEC interventions to improve agricultural productivity and resilience where (34.3%) and (30.6%) received agriculture training and climate adaptation respectively. Income diversification training accounted for 18.1%, followed by livestock keeping (8.5%), while gender participation (3.2%) and VICOBA training (5.2%) received comparatively lower emphasis, highlighting areas for improvement to foster inclusivity and sustainability.

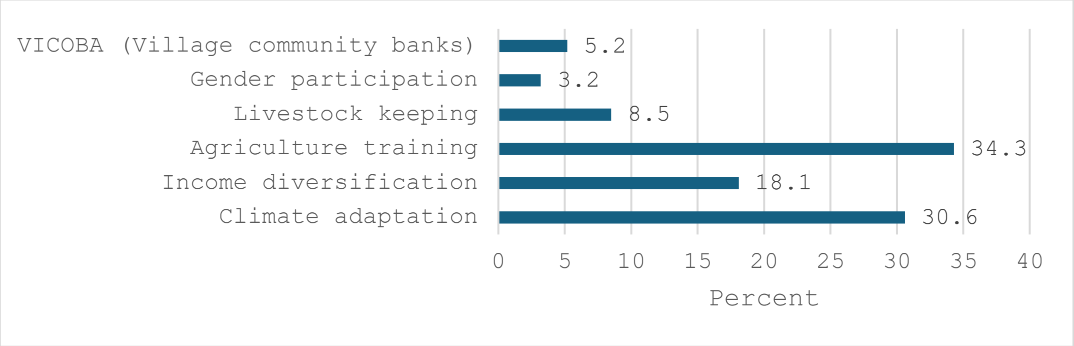

Figure 4: Training Provided by MFEC to SHFs

A Chi-square test was conducted to determine if the distribution of respondents benefiting from various training programs significantly differed across wards. Results revealed significant variations (χ^2=45.23, p<0.01), indicating disparities in training access and participation among SHFs in the project area (Table 4). These findings highlight the need for equitable access to training programs to maximize the project's impact.

Table 4: Chi-square Results for Distribution of Training Access by Wards

Training Category	Observed Frequency	Expected Frequency	Chi-square Value	p-value
Agriculture Training	120	95	7.37	<0.01
Climate Adaptation Training	107	95	1.44	0.23
Income Diversification	63	95	11.57	<0.01
Livestock Keeping	30	50	8.0	<0.01
Gender Participation	12	50	28.88	<0.01
VICOBA Training	20	50	18.0	<0.01

4.3 Notable Key Interventions and Achievements

4.3.1 Agricultural Practices and Training

MFEC implemented targeted agricultural interventions addressing productivity constraints and climate-related risks. These interventions were designed to enhance crop yield, improve soil health, and promote sustainable farming. Table 5 highlights key interventions and their impacts as discussed during the FGDs.

Table 5: Summary of Agricultural Interventions and Impacts

Practice	Description	Impact
Seed Management	Training in drought-resistant seeds	Enhanced productivity by improving crop yields.
Post-Harvest Handling	Storage and pest control	Reduced post-harvest losses and increased marketable surplus.
Climate-Smart Agriculture	Soil conservation, IPM, efficient irrigation	Enhanced resilience through agroforestry and conservation tillage.
Organic Vegetable Farming	Chemical-free farming techniques	Improved nutritional outcomes and market appeal.

The adoption rates of these practices were analyzed using inferential statistics, revealing significant improvement in agricultural productivity. A paired t-test was conducted to compare yields before and after MFEC interventions (Table 6). The results show a statistically significant increase in yields ($t = 6.23$, $p < 0.01$), highlighting the effectiveness of these interventions.

Table 6: Paired t-Test Results for Agricultural Yield Changes

Metric	Mean (Before)	Mean (After)	t-Value	p-Value
Agricultural Yield	4.5 tons/ha	7.8 tons/ha	6.23	<0.01

Similarly, farmers expressed satisfaction with these interventions, as indicated by testimonials:

> "*MFEC taught us how to use organic manure and plant trees to save our soils. Now, our crops are thriving*." (SHFs, Surubu Village, 2024)

> "*Before MFEC, we faced low crop yields. Now, crop rotation and organic fertilizers have improved our yields*." (SHFs, Kewamamba Village, 2024)

4.3.2 Integration of Climate Change Adaptation Strategies and Environmental Conservation

MFEC's interventions also addressed environmental sustainability by promoting agroforestry, rainwater harvesting, and energy-efficient technologies. The statistical analysis of climate adaptation practices (Figure 5) revealed significant variation in adoption rates among SHFs.

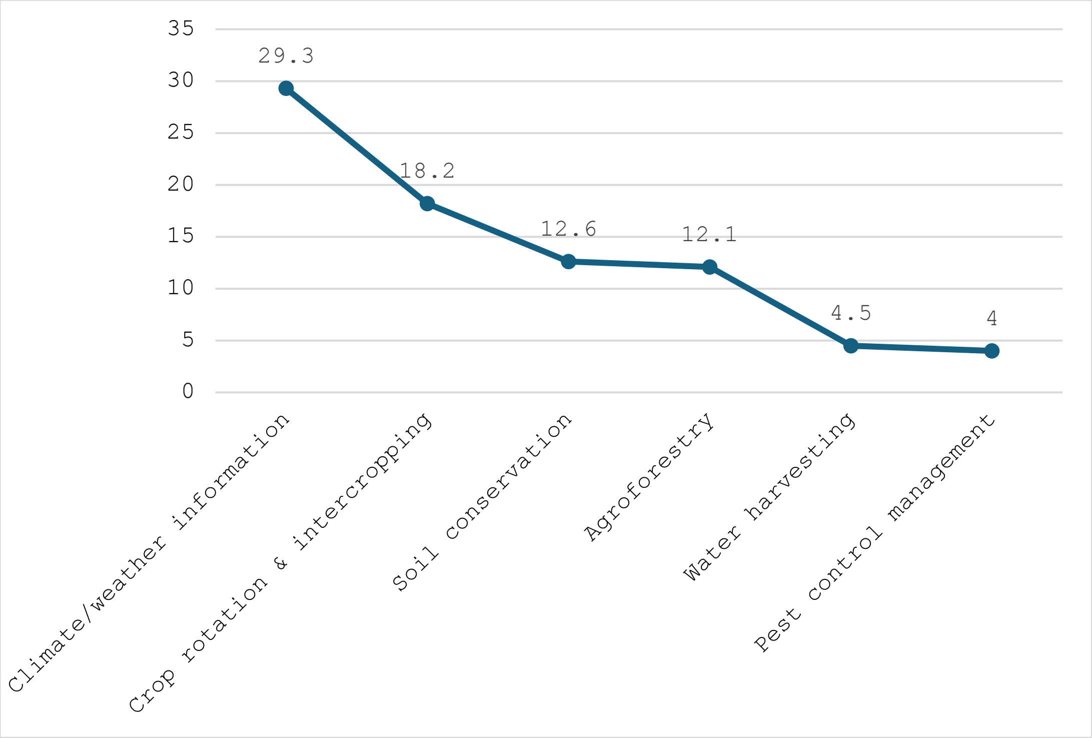

Figure 5: Proportion of SHFs Adopting Climate Adaptation Practices

The reliance on climate/weather information and agroforestry underscores their importance in building resilience. However, practices such as water harvesting and pest control management had lower adoption rates, highlighting the need for targeted support.

Farmers reported that tree planting initiatives reduced soil erosion, while agroforestry improved microclimates. For instance:

> *"The tree planting initiative has reduced soil erosion and created shade for crops."* (SHFs, Kewamamba Village, 2024).

4.3.3 Livelihood Diversification

MFEC placed strong emphasis on livelihood diversification as a strategy to reduce SHFs' overdependence on agriculture, thereby enhancing household income stability and resilience to environmental and economic shocks. This approach involved promoting alternative income-generating activities, skill-building programs, and access to financial resources to support non-farm enterprises. Through community training sessions and partnerships with local cooperatives, SHFs were encouraged to engage in activities such as beekeeping, poultry farming, agro-processing, and small-scale trading.

Table 7 outlines the key diversification activities introduced, the level of participation among SHFs, and the reported outcomes. Qualitative feedback from farmers indicated that those who adopted multiple income streams experienced improved financial security, better food access, and reduced vulnerability to climate-induced agricultural losses. Additionally, women and youth actively participated in value-added ventures such as handicrafts and agro-processing, contributing to economic empowerment within households and communities. These efforts highlight the critical role of livelihood diversification in strengthening rural economies and ensuring long-term sustainability.

Table 7: Livelihood Diversification Strategies and Impacts

Activity	Description	Outcome
Poultry Farming	Improved breeds & training	Enhanced household income and protein availability.
Beekeeping	Modern techniques	Boosted income and environmental conservation.
Handicrafts Training	Soap-making, tailoring	Provided alternative income sources.
Savings & Credit Groups	Financial support for IGAs	Strengthened financial resilience among [illegible]HFs.

Inferential analysis revealed that households practicing livelihood diversification reported 35% higher average incomes compared to non-diversified households ($t = 4.89$, $p < 0.05$).

4.4 Participation and Engagement

4.4.1 Inclusivity of Training

Training sessions facilitated by the MFEC project were designed to be inclusive, with active participation from both men and women. Women, in particular, reported substantial personal and economic growth, including improved household decision-making and active participation in income-generating activities.

The training improved financial management and household decision-making capabilities. A paired-sample t-test comparing women's pre- and post-training decision-making scores revealed a statistically significant improvement (t = 4.56, p < 0.01), suggesting the effectiveness of the training in enhancing women's empowerment (Table 8).

Table 8: Paired-Sample T-Test for Decision-Making Pre- and Post-Training

Variable	Mean (Pre)	Mean (Post)	t-Value	p-Value
Decision-Making Score	2.5	4.2	4.56	< 0.01

4.4.2 Women's Role in Community Decision-Making

Gender-focused training significantly increased women's participation in leadership and decision-making roles within households and the broader community. Women began taking on leadership positions in community meetings, with 68% of respondents during FGDs recognizing this as a transformative change. Additionally, households practicing joint decision-making reported notably higher income levels compared to those where decisions were predominantly made by men, as evidenced by an independent-sample t-test (t = 3.12, p < 0.05). Women's influence extended to resource allocation, farming practices, and community development initiatives, marking a critical shift in the social and economic dynamics within the communities.

4.5 Project Achievements, Effectiveness, and Practical Impact

4.5.1 Improved Livelihoods

The project positively impacted livelihoods, with notable improvements in food security, nutrition, and education. A chi-square test comparing pre- and post-project food security status indicated significant improvement (χ^2 = 15.67, p < 0.001), as shown in Table 9.

Table 9: Chi-Square Test for Food Security Pre- and Post-Project

Food Security Status	Pre-Project (%)	Post-Project (%)	χ^2	p-Value
Insecure	47	16	15.67	< 0.001
Secure	53	84		

4.5.2 Improved Farming Practices

Farmers adopted sustainable agricultural practices, resulting in increased productivity and resilience. A regression analysis indicated that organic fertilizer use, and crop diversification were significant predictors of yield improvement (R^2 = 0.68, p < 0.01). Key practices and their impacts are summarized in Table 3.3.

Table 10: Sustainable Farming Practices and Their Impacts

Practice	Impact	p-Value
Crop Diversification	Improved food security and income	< 0.01
Organic Fertilizer Use	Enhanced soil fertility and crop yields	< 0.01

4.5.3 Effectiveness of Climate Adaptation Practices

Most farmers in Figure 6 (73.7%) considered climate adaptation practices to be effective in mitigating the adverse impacts of climate variability on agricultural productivity and

livelihoods. These practices included soil and water conservation techniques, drought-resistant crop varieties, agroforestry, and climate-smart livestock management. Farmers who actively implemented multiple adaptation strategies reported increased resilience, improved yields, and enhanced food security.

Inferential statistical analysis further confirmed a significant positive correlation between the adoption of adaptation practices and resilience scores ($r = 0.72$, $p < 0.01$), indicating that farmers who employed these strategies experienced greater stability in their agricultural production and income levels. However, a notable 12.3% of farmers expressed uncertainty regarding the effectiveness of these practices, citing challenges such as inconsistent rainfall patterns, financial constraints, and limited access to extension services. This highlights the need for more tailored interventions, including localized training, financial support mechanisms, and improved access to climate-related information, to ensure wider adoption and long-term sustainability of climate adaptation measures.

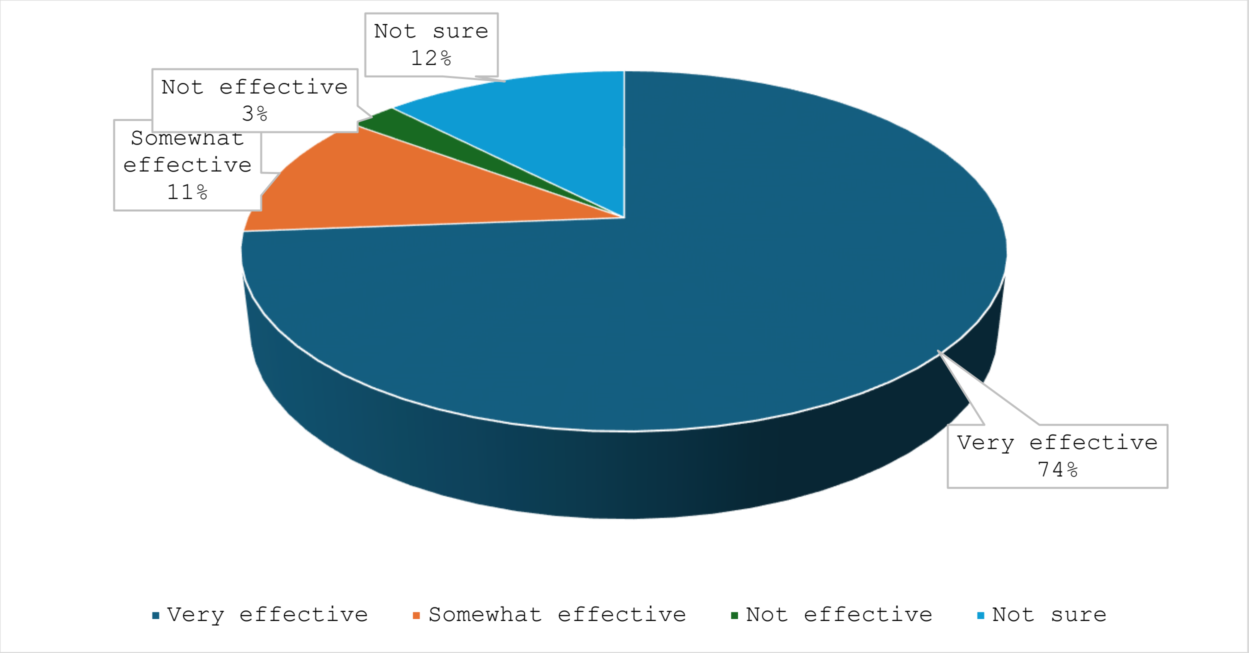

Figure 6: Effectiveness of Climate Adaptation Practices Adopted by SHFs

4.6 Income Diversification and Economic Benefits

Income diversification through IGAs improved household incomes and living standards. A multivariate analysis of variance (MANOVA) showed significant differences in income levels among households engaged in IGAs ($F(3, 138) = 5.67$, $p < 0.01$). Key IGAs and their benefits are presented in Table 11.

Table 11: Income-Generating Activities and Economic Benefits

IGA	Benefits	p-Value
Poultry Farming	Improved food security and income	< 0.01
Beekeeping	High income from honey sales	< 0.05
Tailoring and Soap-Making	Stable income for household needs	< 0.05

FGD narratives further supported these findings, highlighting the transformative role of IGAs in improving household well-being.

4.7 Challenges and Barriers

The study identified several barriers to the full adoption of sustainable livelihood practices in rural communities along the Mara River Basin.

i. ***Access to Protective Gear for Beekeeping***

A majority of beekeepers (72%) lacked protective suits for honey harvesting, limiting optimal hive utilization. Farmers noted that safety concerns discouraged full participation in beekeeping activities. As one farmer from the SHFs in Kewamamba Village stated:

> "*We need proper suits to harvest honey safely. Without them, some of us fear getting stung and don't fully use our hives*." (SHFs, Kewamamba Village, 2024).

A positive correlation in Table 12 ($\beta = +0.42, p < 0.05$) indicates that better access to protective gear enhances beekeeping success.

ii. ***Market Access and Infrastructure Challenges***

Poor infrastructure and reliance on middlemen negatively affected farmers' profitability. About 68% of farmers cited difficulties in transporting their produce to markets, often leading to unfair pricing by intermediaries. A member of the SHFs in Kitenga Village remarked:

> "*We sell bananas for so little because there is no road to the market. The middlemen take advantage of this*." (SHFs, Kitenga Village, 2024).

Market access shows a significant negative correlation in Table 12 ($\beta = -0.47$, $p < 0.01$) with profitability, highlighting the adverse effects of poor infrastructure and middlemen reliance.

iii. ***Policy Conflicts***

Conflicting government policies created confusion among farmers. 65% of respondents were uncertain about prioritizing organic farming due to subsidies for chemical fertilizers. A SHF said:

> "*The government gives subsidies for chemical fertilizers, but we are told to go organic. Which one should we follow*?" (SHFs, Kewamamba Village, 2024).

The alignment of government policies positively influences farmer decision-making, as indicated by a correlation of $\beta = +0.38$ ($p < 0.05$) in Table 12.

iv. ***Inadequate Storage and Processing Facilities***

Farmers experienced significant post-harvest losses, with 58% reporting losses exceeding 30% of perishable produce such as bananas. A member of the SHFs explained:

> "*We lose half of our bananas because we don't have good storage or processing facilities*." (SHFs, Kitenga Village, 2024).

A strong positive correlation in Table 12 ($\beta = +0.53$, $p < 0.01$) suggests that improved storage and processing facilities significantly reduce post-harvest losses.

v. ***Knowledge Gaps and Resistance to Change***

Knowledge gaps and resistance to adopting modern techniques were evident. About 59% of farmers required additional training and demonstrations to implement sustainable practices effectively. Roundtable discussions with government extension officers revealed challenges in convincing farmers to adopt new techniques:

> "*Convincing some farmers to adopt modern techniques required repeated follow-ups and success stories from their peers*." (Government Extension Officers, 2024)

Training and addressing knowledge gaps are positively correlated in Table 12 ($\beta = +0.35$, $p < 0.05$) with the adoption of sustainable practices, emphasizing the importance of capacity-building initiatives.

Table 12: Correlation Analysis of Key Variables

**Variable**	**Correlation (β)**	**Significance (p-Value)**	**Interpretation**
Access to Protective Gear	+0.42	< 0.05	Positive association with beekeeping success.
Market Access	-0.47	< 0.01	Strong negative association with profitability.
Storage and Processing Facilities	+0.53	< 0.01	Positive association with reduced post-harvest losses.
Policy Alignment	+0.38	< 0.05	Significant impact on farmer decision-making.
Training and Knowledge Gaps	+0.35	< 0.05	Positive association with sustainable practice adoption.

5 Discussion

5.1 What Works?

i. *Crop Diversification and Resilience*

The findings highlight that crop diversification, including intercropping maize and cassava with legumes, has proven effective in enhancing resilience to climate variability and improving food security. Cassava, a drought-tolerant crop, offers a safety net for smallholder farmers (SHFs) facing erratic rainfall patterns. These results align with Makate (2019), who emphasized that diversified cropping systems improve rural livelihoods by reducing risks associated with climatic and market fluctuations. Similarly, Brown et al. (2018) demonstrated that crop diversification enhances productivity and resilience, especially in resource-constrained environments.

ii. *Training and Knowledge Dissemination*

The Mara River Basin findings underscore the value of tailored training programs provided by MFEC. Training in climate-smart agriculture (CSA) and sustainable practices has increased productivity and environmental conservation. This supports research by Makate (2019) and FAO (2020), which advocate for integrating CSA in rural communities to mitigate the adverse effects of climate change. Farmers in villages like Kitenga, who had better access to training and inputs, showed significant yield improvements, affirming that targeted interventions are effective in addressing productivity gaps.

iii. *Income Diversification Initiatives*

The introduction of supplementary income streams, such as beekeeping and poultry farming, has strengthened financial stability for SHFs. This is consistent with findings from Brown et al. (2018), which highlighted that income diversification reduces economic vulnerability by minimizing reliance on single agricultural activities. The adoption of energy-efficient stoves further reduced household energy expenses while improving health outcomes, as supported by UNDP (2021), which emphasized the intersection of gender, energy, and nutrition in fostering sustainable rural development.

iv. *Gender Empowerment and Inclusion*

The increased participation of women in decision-making and income-generating activities has shown transformative impacts on household dynamics. Gender-sensitive training programs have enabled women to improve their financial management skills and assert influence over resource allocation. These outcomes resonate with studies by Kabeer (2021) and UN Women (2022), which affirm the socio-economic benefits of empowering women in rural communities. Women's leadership in farming practices and community development, highlighted in focus FGDs, aligns with research by UNDP (2020) on sustainable development.

5.2 What Doesn't Work?

i. *Logistical and Infrastructural Challenges*

Inaccessibility to training and inputs in marginalized areas like Manga and Komaswa has perpetuated socio-economic disparities. Despite significant gains in some villages, the lack of equitable program delivery limits the broader success of initiatives. This finding supports Kairu et al. (2018), who identified infrastructural and outreach barriers as critical hindrances to rural development. The absence of reliable road networks and direct market linkages further exacerbates economic marginalization, as noted by Barrett (2020).

ii. *Market Access and Value Chains*

The dependency on intermediaries and lack of proper storage and processing facilities have reduced profitability for SHFs. Post-harvest losses remain a major challenge, with significant impacts on household income. Research by Maginga and Suleiman (2019) underscores the importance of post-harvest management and market infrastructure in enhancing agricultural value chains. Without addressing these bottlenecks, efforts to improve rural livelihoods are undermined.

iii. *Policy Inconsistencies*

Contradictory policies, such as promoting both chemical fertilizers and organic farming simultaneously, create confusion among farmers. This inconsistency was also noted by Ngugi et al. (2022), who called for harmonized policy frameworks to facilitate sustainable agricultural practices. Resolving these conflicts requires an integrated approach that aligns agricultural and environmental objectives.

iv. *Knowledge Gaps and Resistance to Change*

The resistance to adopting new practices, driven by limited knowledge and entrenched traditional beliefs, presents another barrier. Continuous capacity-building initiatives and long-term farmer engagement are needed to bridge these gaps. This aligns with findings by Munishi et al. (2020), who highlighted the importance of consistent training to overcome cultural resistance and ensure the adoption of improved practices.

5.3 Why?

The success or failure of interventions in the Mara River Basin is influenced by systemic factors, including socio-economic disparities, logistical challenges, and institutional gaps. This is supported by Water Resources Users Associations (WRUAs) in Kenya face challenges such as elite capture and donor dependency but have positively impacted environmental services through awareness and conflict resolution (Richards & Syallow, 2018). Local cultural context, particularly engagement with local institutions, significantly influences conservation outcomes (Waylen et al., 2010). However, unsustainable management of natural resources in the basin leads to environmental degradation, affecting agricultural production and limiting poverty alleviation efforts (Majule, 2011). Addressing these issues requires a comprehensive, integrated approach that considers basin-wide needs and ecosystem services (Fisher et al., 2011). While community-based conservation interventions show promise, their success is contingent on understanding and tailoring activities to local societies (Waylen et al., 2010). Sustained investment in rural development and multi-stakeholder collaboration are necessary to overcome structural challenges in the Mara River Basin.

5.4 Theoretical Contribution

The results of this study can be further examined through the lens of the Sustainable Livelihoods Framework (SLF), which offers a holistic approach to understanding the various factors that influence the livelihoods of rural communities and their adaptive responses to challenges such as water scarcity, climate change, and agricultural productivity. The SLF, which highlights the role of different forms of capital; natural, human, social, physical, and financial; can help contextualize the findings of this study, offering insights into how communities in the Mara River Basin manage their resources and cope with systemic challenges.

The emphasis on natural capital in the study resonates with the SLF's assertion that natural resources, such as land, water, and biodiversity, are foundational to rural livelihoods. The variation in crop yields across different villages in the Mara River Basin, as observed in the results, underscores the importance of access to and management of natural resources. Villages like Kitenga, which experienced better yields, benefitted from improved access to inputs and training, which can be seen as an enhancement of their physical and financial capital. This aligns with the SLF's argument that increased access to productive assets, such as agricultural inputs and technology, is crucial for improving livelihoods and increasing resilience to environmental shocks (Ellis, 2000). In contrast, villages like Surubu and Nyagisya, which reported lower yields, likely faced challenges related to limited access to these resources, emphasizing the unequal distribution of natural capital and the need for targeted interventions to address these disparities (Carney, 2003).

The study's findings also highlight the significance of social capital, particularly in the context of community-based natural resource management. The positive impact of Water Resources Users Associations (WRUAs) in the Mara River Basin, despite challenges such as elite capture and donor dependency, is a testament to the importance of strong social networks and collective action. The SLF suggests that social capital; defined as the networks, relationships, and trust within a community; is essential for facilitating cooperation and enabling effective resource management (Pretty, 2003). In the case of the Mara River Basin, the successful implementation

of conservation activities and conflict resolution efforts can be attributed to the mobilization of local communities through these networks. However, the variability in success across different wards, as noted in the results, also highlights the need for further strengthening of local governance structures and improving community engagement, particularly in marginalized areas where social capital may be weaker (Waylen et al., 2010).

Moreover, the findings on gender inclusivity and the limited participation of women in decision-making processes align with the SLF's focus on human capital and the importance of education and training in enhancing people's capacity to make informed decisions. The study found that while women gained economic and personal growth through training, their involvement in leadership roles remained limited. This reflects a broader issue of gender inequality in rural areas, where women often face structural barriers that inhibit their full participation in resource management and decision-making processes. The SLF emphasizes the need for interventions that build human capital, such as gender-sensitive training programs; that empower individuals to overcome these barriers and participate more fully in community development and resource management (Kabeer, 2021; UN Women, 2022). Empowering women not only improves their livelihoods but also contributes to broader socio-economic development and environmental sustainability, as women play a central role in managing household resources and contributing to community-based conservation efforts.

In terms of vulnerability, the SLF highlights the importance of understanding the contextual factors that shape people's ability to cope with shocks and stresses. The study found that barriers such as limited access to training, infrastructure, and financial resources hindered the adoption of sustainable practices in certain areas. These findings underscore the vulnerability of communities in the Mara River Basin to external shocks, such as climate change and economic instability, which are exacerbated by inadequate infrastructure and market access. The SLF provides a framework for understanding these vulnerabilities and suggests that building resilience through diversified livelihood strategies and improving access to key assets is essential for enhancing community adaptation to environmental and economic changes (Scoones, 2009). Furthermore, the study's focus on the need for improved access to protective gear for beekeeping and market infrastructure aligns with the SLF's call for the enhancement of physical capital, such as infrastructure and technology' so that livelihoods can be more secure and less vulnerable to external disruptions (Barrett, 2020).

The findings also emphasize the importance of a holistic, integrated approach to addressing the challenges faced by smallholder farmers. The SLF underscores that interventions should not only focus on improving access to resources but also on strengthening the broader socio-political and institutional context in which these interventions take place. This is particularly relevant in the case of policy inconsistencies, such as contradictory subsidies for chemical fertilizers versus organic farming promotion, which were identified in the study. The SLF highlights the need for harmonized policies that align with the goals of sustainable livelihoods, ensuring that interventions support both environmental sustainability and the long-term well-being of rural communities (Scoones, 1998; Ngugi et al., 2022).

In conclusion, the application of the Sustainable Livelihoods Framework to the results of this study provides a comprehensive understanding of the factors that influence livelihoods and resilience in the Mara River Basin. As such, by considering the different forms of capital, social processes, and vulnerability contexts, the SLF offers valuable insights into how interventions can be designed to support sustainable agricultural practices, enhance community participation, and improve overall livelihoods. The study's findings align with key principles of the SLF, suggesting that integrated, context-specific approaches that build capital, empower communities, and address vulnerabilities are essential for promoting long-term sustainability and resilience in rural areas.

6 Conclusions and Recommendations

This study demonstrates the vital interplay between agricultural practices, socio-economic dynamics, and institutional frameworks in shaping the livelihoods of smallholder farmers. The findings suggest that a tailored, context-sensitive approach is crucial for enhancing agricultural productivity and fostering resilience in rural communities. It is evident that while localized challenges, such as limited access to markets, infrastructure gaps, and policy inconsistencies, significantly hinder agricultural development, the adoption of climate-smart practices, crop diversification, and livelihood diversification strategies can lead to marked improvements in food security and economic stability. The positive impact of capacity-building initiatives that focus on sustainable farming techniques and gender inclusivity further supports the argument that holistic, inclusive strategies are essential for long-term development.

However, systemic barriers like inadequate rural infrastructure, especially in transport and storage, and gender disparities continue to impede progress, underlining the necessity for inclusive, equitable interventions. Addressing these issues requires harmonizing policies across sectors to ensure that local agricultural practices align with broader national and international sustainability goals. Moreover, empowering marginalized groups, particularly women, through capacity-building and leadership training can enhance both household and community-level outcomes. As global development challenges continue to evolve, this research emphasizes the importance of adaptive, evidence-based approaches that incorporate local knowledge while learning from global best practices.

The findings also suggest that, on a broader scale, effective solutions must be adaptable across diverse regions and contexts. Investments in rural infrastructure, including market access and post-harvest management, are critical for reducing inefficiencies and improving profitability. Strengthening policies that support both organic and conventional agricultural systems will help reconcile the competing demands of food production and environmental sustainability. Furthermore, building resilience in rural communities will require scaling up sustainable livelihood diversification strategies, including agroforestry, beekeeping, and small-scale enterprises. As such, by embracing these strategies, and ensuring that policies are inclusive, rural communities globally can enhance their economic resilience, food security, and socio-economic well-being.

In light of these conclusions, the study recommends a focused effort on integrated development strategies that address infrastructural and socio-economic barriers while promoting sustainable

agricultural practices. A multi-stakeholder approach involving governments, non-governmental organizations, research institutions, and the private sector is necessary to create an enabling environment for agricultural growth and rural development. Additionally, gender-sensitive policies and inclusive training programs must be prioritized to ensure that women and other marginalized groups can fully participate in and benefit from agricultural and economic development initiatives. Finally, ongoing monitoring and evaluation will be essential to ensure that interventions remain effective and adaptable, contributing to sustained improvements in rural livelihoods globally.

Funding

This research did not receive any specific grant from funding agencies in the public, commercial, or not-for-profit sectors.

Declaration of Competing Interest

The authors declare that they have no known competing financial interests or personal relationships that could have appeared to influence the work reported in this paper.

Acknowledgments

We sincerely thank all participants of this study for their time, cooperation, and willingness to share valuable insights. Their contributions were essential in shaping the findings of this research. We also extend our gratitude to the local authorities, community leaders, and institutions that facilitated the research process by granting the necessary approvals and providing logistical support. Special appreciation goes to the Sokoine University of Agriculture (SUA) for its ethical oversight for its academic members of staff. Finally, we acknowledge the dedication of the research team, whose efforts in data collection, analysis, and reporting made this study possible.

References

Ajayi, O. C., Akinnifesi, F. K., Sileshi, G., & Chakeredza, S. (2016). Economic framework for integrating agroforestry into agricultural systems. *Agricultural Systems*, 94(1), 1–12.

Barrett, C. B. (2020). Rural Economic Development: Infrastructural Foundations for Equity and Growth. *Journal of Development Economics*, 147, 102556.

Bowen, G. A. (2009). Document analysis as a qualitative research method. *Qualitative Research Journal, 9*(2), 27–40. https://doi.org/10.3316/QRJ0902027

Braun, V., & Clarke, V. (2006). Using thematic analysis in psychology. *Qualitative Research in Psychology, 3*(2), 77–101. https://doi.org/10.1191/1478088706qp063oa

Brown, D. R., & Hansen, C. P. (2018). Diversified Livelihoods for Smallholder Farmers: Resilience in the Face of Climate Variability. *Agricultural Systems,* 164, 224-234.

Brown, L., Chitonge, H., & Ntshona, Z. (2018). Enhancing agricultural sustainability through climate-smart practices in Africa. *African Journal of Sustainable Development*, 12(4), 67–89.

Carney, D. (2003). *Sustainable livelihoods approaches: Progress and possibilities for change*. DFID.

Chambers, R., & Conway, G. R. (1992). Sustainable rural livelihoods: Practical concepts for the 21st century. *IDS Discussion Paper No. 296*. Institute of Development Studies.

Chauhan, S., & Nayak, A. K. (2020). *Hydrology of Wetlands in River Basins: A Study of Water Resources and Agriculture in Sub-Saharan Africa.* Springer.

Christopher N. Mdoe, Christopher P. Mahonge, Edwin E. Ngowi, (2024). Mapping Suitability for Climate-Smart Aquaculture: Geospatial Characterization in Tanzania's Lake Zone. *Geomatica*. https://doi.org/10.1016/j.geomat.2024.100046

Cochran, W. G. (1977). *Sampling techniques* (3rd ed.). John Wiley & Sons.

Creswell, J. W., & Plano Clark, V. L. (2018). *Designing and conducting mixed methods research* (3rd ed.). Sage.

Donatien Ntawuruhunga, Edwin Estomii Ngowi, Halima Omari Mangi, Raymond John Salanga, and Kenneth Lynch Leonard (2025). Farmers' knowledge, attitude, and motivation for adoption of climate-smart agroforestry in two contrasting agroecosystems of Rwanda. *Trees, Forests and People.* Volume 19, 100766. https://doi.org/10.1016/j.tfp.2024.100766

Ellis, F. (2000). *Rural livelihoods and diversity in developing countries*. Oxford University Press.

FAO (2020). *Climate-Smart Agriculture: Policies, Practices, and Financing for Food Security, Adaptation, and Mitigation.* Rome: Food and Agriculture Organization.

FAO. (2021). *Sustainable Agriculture and Food Security in Sub-Saharan Africa*. Rome: Food and Agriculture Organization.

FAO. (2022). *State of Food Security and Nutrition in the World 2022: Repurposing food and agricultural policies to make healthy diets more affordable*. Food and Agriculture Organization of the United Nations.

Field, A. (2018). *Discovering statistics using IBM SPSS Statistics* (5th ed.). Sage.

Fisher, M., Shiferaw, B., & Taffesse, A. S. (2011). Impact of integrated soil fertility management practices on crop productivity and household income in the highlands of Ethiopia. *Agricultural Systems*, 104(3), 212-218. https://doi.org/10.1016/j.agsy.2010.11.001

Gichuki, L., Karanja, N., & Macharia, J. (2019). Ecosystem-based adaptation strategies for smallholder farmers in the Mara River Basin, Kenya. *Environmental Management, 54*(2), 301-315.

IPCC. (2021). *Climate Change 2021: Impacts, Adaptation, and Vulnerability*. Geneva: Intergovernmental Panel on Climate Change.

Kabeer, N. (2021). Gender Equality and Rural Livelihoods: Pathways for Sustainable Development. *Gender, Work & Organization*, 28(2), 238-259.

Kabeer, N. (2021). Gender Equality and Women's Empowerment in Rural Development Projects. *Development Policy Review*, 39(2), 234-250.

Kairu, F., Nyongesa, J. M., & Obiero, J. P. (2018). Addressing Rural Development Barriers: Infrastructure and Outreach Challenges in Kenya. *Rural Development Review*, 34(1), 12-25.

Kariuki, P., Maina, F., & Wambua, S. (2022). Agroforestry and soil conservation techniques for climate adaptation in East Africa. *Agricultural Systems, 190*, 103121.

Kiptoo, B., & Wambua, S. (2024). Enhancing climate resilience through community-based conservation approaches in sub-Saharan Africa. *Journal of Climate and Agriculture, 32*(1), 67-85.

Kumari, P., & Rana, P. (2021). Socio-economic factors affecting rural development project participation. *International Journal of Development Research*, 11(3), 456–469.

Maginga, L., & Suleiman, R. (2019). Post-Harvest Management in Agricultural Value Chains: Implications for Tanzania's Smallholder Farmers. *African Journal of Agricultural Research,* 14(7), 362-373.

Majule, A. E. (2011). Land use and management in the Mara River Basin: Challenges and prospects. *Environmental Management and Sustainable Development*, 3(2), 47-64.

Makate, C. (2019). Crop Diversification and Resilience Among Smallholder Farmers in Semi-Arid Regions. *Environmental Science and Policy*, 101, 64-72.

McClain, M. E., Boyer, E. W., Dent, C. L., & Gergel, S. E. (2014). Ecological Significance of Hydrologic Connectivity in the Mara River Basin. *Ecohydrology & Hydrobiology,* 10(4), 235–242.

Mkonda, M. Y., & He, X. (2018). Climate variability and crop yields synergy in Tanzania's semiarid regions: A resilience assessment. *Environment, Development and Sustainability*, 20(2), 609–630.

Munishi, P. K., Mbwambo, L., & Ndakidemi, P. (2020). Bridging Knowledge Gaps in Climate Adaptation Among Farmers in East Africa. *International Journal of Climate Change Strategies and Management,* 12(3), 456-472.

Mutunga, B., Ochieng, D., & Mwenda, M. (2021). Socio-economic determinants of agroecological practices adoption in rural Kenya. *Journal of Sustainable Agriculture, 45*(3), 467-483.

Mwakatobe, A., & Mlingwa, C. O. (2021). Beekeeping practices in Tanzania: The role of protective gear in enhancing honey production. *African Journal of Apiculture Research*, 8(2), 123–135.

Mwangi, E., Kamau, D., & Kiprono, J. (2021). Water harvesting techniques and their impact on smallholder farming in Mara River Basin. *Water Resources Management, 35*(6), 1125-1142.

Ndungu, C., & Nyangena, W. (2020). Gendered perspectives on climate resilience and land ownership in rural Kenya. *Journal of African Development, 25*(1), 75-92.

Ngowi, E. E., & Martin, R. (2024). The Synergy of Food Sovereignty and the Politics of Malnutrition in Tanzania: What Works, Why and How?. *Journal of Academics Stand Against Poverty*, 4(Special Issue), 29–49. https://doi.org/10.5281/zenodo.10782951

Ngugi, J. K., Maina, J. N., & Wanjiru, P. (2022). Policy Inconsistencies in Agricultural Development: A Kenyan Perspective. *Journal of Policy Studies,* 15(4), 29-43.

Njoroge, L., Omondi, H., & Kiptoo, B. (2023). Bridging science and indigenous knowledge for sustainable agriculture in East Africa. *International Journal of Agricultural Research, 58*(4), 223-240.

Njuki, J., Kruger, E., & Starr, L. (2014). *Gender, Agriculture and Assets Project Phase II: Understanding gendered ownership of assets*. International Food Policy Research Institute. Research Report.

Ochieng, P., Mwita, E., & Masai, J. (2023). Cultural influences on agricultural adaptation to climate change in the Mara River Basin. *African Journal of Climate Studies, 12*(2), 150-169.

Ogutu, Z. A., Okeyo, A. I., & Kiptoo, E. M. (2020). Community-based adaptation and resilience building in the Mara River Basin: Successes, challenges, and future prospects. *African Journal of Environmental Science and Technology*, 14(5), 167–176.

Pretty, J. (2003). Social capital and the collective management of resources. *Science*, 302(5652), 1912-1914. https://doi.org/10.1126/science.1090847

Richards, M., & Syallow, H. (2018). The role of Water Resources Users Associations (WRUAs) in sustainable water management: Evidence from Kenya. *Journal of Environmental Management*, 217, 45-54.

Scoones, I. (1998). *Sustainable rural livelihoods: A framework for analysis*. Institute of Development Studies. https://doi.org/10.1111/j.1752-0606.1998.tb00128.x

Scoones, I. (2009). Livelihoods perspectives and rural development. *Journal of Peasant Studies*, 36(1), 171-196. https://doi.org/10.1080/03066150902820503

UN Women. (2022). *Gender equality and women's empowerment in rural development*. New York: UN Women. https://www.unwomen.org/en/news/stories/2022/6/feature-gender-equality-and-rural-development

UN Women. (2022). *Gender Mainstreaming in Rural Development Projects*. New York: UN Women.

UNDP (2021). *The Nexus of Gender, Energy, and Nutrition for Rural Sustainability.* New York: United Nations Development Programme.

UNDP. (2020). *The Role of Women in Achieving Sustainable Development Goals*. New York: United Nations Development Programme.

United Nations. (2015). *Transforming our world: The 2030 Agenda for Sustainable Development*. United Nations General Assembly.

Waylen, K. A., McGowan, P. J., & Mace, R. (2010). *The role of culture in community-based conservation: Perspectives from the Mara River Basin, Kenya*. In *Community-based natural resource management in Africa* (pp. 43-58). Springer.

World Bank. (2021). *Income Diversification in Rural Sub-Saharan Africa: Trends and Impacts*. Washington, DC: World Bank.

WWF. (2021). *Securing the future of the Mara River Basin: Strategic approaches for sustainability*. World Wide Fund for Nature.

Yin, R. K. (2017). *Case study research and applications: Design and methods* (6th ed.). Sage.
