# Supplementary material for: Evaluating Effectiveness of Sustainable Livelihood Development in Rural Communities along Mara River Basin, Tanzania: What Works, What Doesn’t Work, and Why?
Source: PLoS One. 2026 Jun 11;21(6):e0351252. doi: 10.1371/journal.pone.0351252 (PMC13258000; doi:10.1371/journal.pone.0351252)
Supplement: Attachment — Submitted filename: Response_to_Reviewers_auresp_3.docx [file pone.0351252.s010.docx]

**Response to Reviewer Comments**

**Manuscript Title:** “Evaluating Effectiveness of Sustainable Livelihood Development in Rural Communities along Mara River Basin, Tanzania: *What Works, What Doesn’t Work,* and *Why*?”

**Authors:** Edwin E. Ngowi and Angela M. Jesse

**Dear Reviewer,**

We sincerely thank you for your thoughtful and constructive comments on our manuscript. Your insights were invaluable in guiding the revision process. Thank you for this important comment. The manuscript has been thoroughly revised to comply with PLOS ONE referencing requirements using the Vancouver citation style. Specifically, (i) all references have now been compiled into a numbered reference list placed at the end of the manuscript, (ii) in-text citations have been converted from author–date format to numerical format in the order of appearance, and (iii) DOIs have been added where available. All citations have been cross-checked to ensure consistency between in-text numbering and the reference list. Below, we provide a point-by-point response to each comment, describing the actions we took to address your concerns. All changes were made to the revised manuscript.

**Reviewer Comment 1:**
*The Sustainable Livelihoods Framework is introduced but not consistently applied in the discussion, limiting analytical depth.*

**Author Response:**

We agreed with this observation. We therefore restructured the entire Discussion section (Section 4) to explicitly apply the Sustainable Livelihoods Framework (SLF) to each major finding. We reorganized the discussion under three sub-headings that directly map onto the SLF components: “What Works? Interpreting Successes through the SLF” (linking findings to Livelihood Assets), “What Doesn't Work? Systemic Barriers as Failures of Transforming Structures” (linking to Transforming Structures and Processes), and “Theoretical and Practical Contributions.” Each paragraph now explicitly names the relevant SLF concept (e.g., human capital, social capital, physical capital, transforming structures) and explains how the finding supports or challenges the framework.

**Location of Change in Manuscript:**

Section 4 (Discussion), sub-sections 4.1, 4.2, and 4.3 (pages 28-31 of the revised manuscript).

**Reviewer Comment 2:**

*Several figures and tables are difficult to interpret, and statistical reporting is inconsistent, for example, in p-value formats.*

**Author Response:**

We accepted this criticism and took the following actions. First, we standardized all p-value reporting throughout the manuscript. All p-values greater than 0.001 were reported as exact values (e.g., p = 0.03, p = 0.004), while values below 0.001 were reported as p < 0.001. Second, we revised the titles of several tables and figures to make them more descriptive and self-explanatory. For example, Table 7 was retitled from “Paired t-Test Results for Agricultural Yield Changes” to “Impact of MFEC Training on Agricultural Yield: A Paired t-Test Comparison of Before-and-After Intervention (n=265)”. Similarly, Figure 6 was retitled from “Effectiveness of Climate Adaptation Practices Adopted by SHFs” to “Smallholder Farmer Perceptions of the Effectiveness of Different Climate Adaptation Practices (n=265)”.

**Location of Changes in Manuscript:**

- Standardized p-values: Throughout the Results (Section 4) and Discussion (Section 5) sections.
- Table 7: Page 18
- Figure 6: Page 21

**Reviewer Comment 3:**

*The discussion is overly long and repeats results rather than engaging with prior literature or clearly articulating contributions.*

**Author Response:**

We agreed with this assessment. We therefore substantially shortened and refocused the Discussion section. We removed all repetitive content that merely restated results from Section 4. Instead, we reframed the discussion to interpret the *meaning* of the key findings, explicitly compared and contrasted our results with the cited literature (e.g., FAO, 2020; Barrett, 2020; Kabeer, 2021), and added a new, dedicated sub-section titled “Theoretical and Practical Contributions” (Section 4.3). This new sub-section clearly articulates the study’s two main contributions: empirical validation of the SLF in a data-scarce context and the provision of a replicable, low-cost evaluation protocol for practitioners.

**Location of Change in Manuscript:**

Section 4 (Discussion), entirely replaced with new text on pages 28-31.

**Reviewer Comment 4:**

*Kindly add more practical recommendations.*

**Author Response:**

We agreed that the original recommendations were too general. We therefore added a new, numbered sub-section titled “Specific, Actionable Recommendations for Policy and Practice” at the end of the Conclusions section. These recommendations were organized by target audience (District/National Government, Implementing NGO, Development Partners) and were made specific, measurable, and directly tied to the study’s findings (e.g., creating a voucher scheme for organic inputs to address policy confusion, developing a revolving fund for beekeeping protective gear based on the correlation *β* = +0.42, *p* < 0.05).

**Location of Change in Manuscript:**

Section 5 (Conclusions and Recommendations), added as a new sub-section before “Funding” (pages 34-35 of the revised manuscript).

**Reviewer Comment 5:**

*Language polishing and reference checking, such as DOI, are required.*

**Author Response:**

We took two actions to address this comment. First, we arranged for professional language editing of the entire manuscript, focusing on sentence structure, passive voice, and consistency in terminology (e.g., changing “Low Land” to “Lowland”). Second, we verified every reference in the bibliography and added Digital Object Identifiers (DOIs) where available. For example, we added DOIs for Bowen (2009), Creswell & Plano Clark (2018), and Scoones (2009), among others.

**Location of Change in Manuscript:**

- Language polishing: Throughout the entire manuscript.
- References with added DOIs: Section “References” (pages 37-44).

**Reviewer Comment 6:**

*Such a title, “5.3 Why?” should be improved more smoothly.*

**Author Response:**

We agreed that the original sub-section title was too abrupt. We therefore revised it to a more descriptive and academically professional title. The new title clearly indicates the content of the sub-section.

**Location of Change in Manuscript:**

Section 5.3. The title was changed from “Why?” to “Understanding the Underlying Drivers: Why Barriers Persist and Successes Occur” (page 32).

**Reviewer Comment 7:**

*For “2. The Theoretical Framework,” kindly add the framework showing the interconnection between the main concepts.*

**Author Response:**

We accepted this suggestion. We therefore created a new conceptual diagram (Image 1) that visually synthesizes the interconnections among the core components of the Sustainable Livelihoods Framework as applied to this project. The figure is a flow diagram showing the relationships among the Vulnerability Context, Livelihood Assets (human, social, natural, physical, financial), Transforming Structures and Processes, and Livelihood Strategies and Outcomes. An explanatory paragraph was added immediately preceding the figure.

**Location of Change in Manuscript:**

Section 2 (The Theoretical Framework), at the end of the section, just before Section 3 (Methodology) (page 7).

**Reviewer Comment 8:** *Add the map of the study area.*

**Author Response:**

We noted that a map (Image 2) was already present in the original manuscript. However, to improve its interpretability as suggested, we revised the map caption to be more detailed and descriptive. The new caption explicitly names the ten project villages, the five wards, and the position of the Mara River Basin within East Africa.

**Location of Change in Manuscript:** Section 3.1 (Study Area Description), caption for Image 1 (page 8).

**Summary of Changes**

We believe that the revisions made in response to your comments have substantially improved the manuscript. The theoretical framework is now consistently applied, the data presentation is clearer, the discussion is focused and contributes to the literature, the recommendations are actionable, and the technical quality of the writing and references has been enhanced. We are grateful for your time and expertise in reviewing our work.

We look forward to your final decision.

**Sincerely,**

Edwin E. Ngowi and Angela M. Jesse
