# Supplementary material for: Evaluating Effectiveness of Sustainable Livelihood Development in Rural Communities along Mara River Basin, Tanzania: What Works, What Doesn’t Work, and Why?
Source: PLoS One. 2026 Jun 11;21(6):e0351252. doi: 10.1371/journal.pone.0351252 (PMC13258000; doi:10.1371/journal.pone.0351252)
Supplement: Peer review history

# Peer review: Decision Letter 0

**DOI:** 10.1371/journal.pone.0351252.r001

**Authors:** Juan Carlos Suárez Salazar

3 Jun 2025

PONE-D-25-05903

Evaluating Effectiveness of Sustainable Livelihood Development in Rural Communities along Mara River Basin, Tanzania: What Works, What Doesn’t Work, and Why?

PLOS ONE

Dear Dr. Ngowi,

Thank you for submitting your manuscript to PLOS ONE. After careful consideration, we feel that it has merit but does not fully meet PLOS ONE’s publication criteria as it currently stands. Therefore, we invite you to submit a revised version of the manuscript that addresses the points raised during the review process.

Please submit your revised manuscript by Jul 17 2025 11:59PM. If you will need more time than this to complete your revisions, please reply to this message or contact the journal office at plosone@plos.org. When you're ready to submit your revision, log on to https://www.editorialmanager.com/pone/ and select the 'Submissions Needing Revision' folder to locate your manuscript file.

Please include the following items when submitting your revised manuscript:

- A rebuttal letter that responds to each point raised by the academic editor and reviewer(s). You should upload this letter as a separate file labeled 'Response to Reviewers'.
- A marked-up copy of your manuscript that highlights changes made to the original version. You should upload this as a separate file labeled 'Revised Manuscript with Track Changes'.
- An unmarked version of your revised paper without tracked changes. You should upload this as a separate file labeled 'Manuscript'.

If you would like to make changes to your financial disclosure, please include your updated statement in your cover letter. Guidelines for resubmitting your figure files are available below the reviewer comments at the end of this letter.

If applicable, we recommend that you deposit your laboratory protocols in protocols.io to enhance the reproducibility of your results. Protocols.io assigns your protocol its own identifier (DOI) so that it can be cited independently in the future. For instructions see: https://journals.plos.org/plosone/s/submission-guidelines#loc-laboratory-protocols. Additionally, PLOS ONE offers an option for publishing peer-reviewed Lab Protocol articles, which describe protocols hosted on protocols.io. Read more information on sharing protocols at https://plos.org/protocols?utm_medium=editorial-email&utm_source=authorletters&utm_campaign=protocols.

We look forward to receiving your revised manuscript.

Kind regards,

Juan Carlos Suárez Salazar

Academic Editor

PLOS ONE

Journal Requirements:

When submitting your revision, we need you to address these additional requirements.

1. Please ensure that your manuscript meets PLOS ONE's style requirements, including those for file naming. The PLOS ONE style templates can be found at

https://journals.plos.org/plosone/s/file?id=wjVg/PLOSOne_formatting_sample_main_body.pdf and

https://journals.plos.org/plosone/s/file?id=ba62/PLOSOne_formatting_sample_title_authors_affiliations.pdf

2. We note that your Data Availability Statement is currently as follows: [All relevant data are within the manuscript and its Supporting Information files.]

Please confirm at this time whether or not your submission contains all raw data required to replicate the results of your study. Authors must share the “minimal data set” for their submission. PLOS defines the minimal data set to consist of the data required to replicate all study findings reported in the article, as well as related metadata and methods (https://journals.plos.org/plosone/s/data-availability#loc-minimal-data-set-definition).

For example, authors should submit the following data:

- The values behind the means, standard deviations and other measures reported;

- The values used to build graphs;

- The points extracted from images for analysis.

Authors do not need to submit their entire data set if only a portion of the data was used in the reported study.

If your submission does not contain these data, please either upload them as Supporting Information files or deposit them to a stable, public repository and provide us with the relevant URLs, DOIs, or accession numbers. For a list of recommended repositories, please see https://journals.plos.org/plosone/s/recommended-repositories.

If there are ethical or legal restrictions on sharing a de-identified data set, please explain them in detail (e.g., data contain potentially sensitive information, data are owned by a third-party organization, etc.) and who has imposed them (e.g., an ethics committee). Please also provide contact information for a data access committee, ethics committee, or other institutional body to which data requests may be sent. If data are owned by a third party, please indicate how others may request data access.

***Comments from PLOS Editorial Office:* We note that one or more reviewers has recommended that you cite specific previously published works. As always, we recommend that you please review and evaluate the requested works to determine whether they are relevant and should be cited. It is not a requirement to cite these works. We appreciate your attention to this request.**

Additional Editor Comments:

The manuscript titled "Evaluating Effectiveness of Sustainable Livelihood Development in Rural Communities along Mara River Basin, Tanzania: What Works, What Doesn’t Work, and Why?" comprehensively addresses the complex environmental, social, political, and cultural challenges facing rural communities in the Mara River Basin. This holistic approach is appropriate and demonstrates originality by analyzing sustainable livelihoods as a key element for transforming the local economy. However, several areas for improvement have been identified to enhance the quality and impact of the study.

The introduction should provide a more detailed description of the demographic, geographic, social, and cultural characteristics of the rural communities in the lower Mara River Basin to better contextualize the research. Including a map of the basin and its rural communities would also be valuable. Additionally, while issues such as environmental degradation and poverty are mentioned, their magnitude should be quantified to emphasize their significance. In the theoretical framework, the conclusions drawn in the final paragraph appear overly ambitious given the limited number of references cited. Expanding the bibliographic base to better support these claims and articulating the findings with previous studies would strengthen this section.

The methodology section requires further elaboration to ensure clarity and transparency. Specifically, the criteria used to select the 265 farmers from the total population of 850 should be detailed, including considerations such as levels of vulnerability, crop types, or agricultural practices. It is also important to explain how diversity in terms of gender, age, and socioeconomic status was addressed. Justifying the sample size and explaining its representativeness for the entire population of farmers in the basin is crucial to validate the generalizability of the study's conclusions. Regarding the focus groups, it is recommended to describe the diversity of participants, including farmers, community leaders, NGO representatives, local authorities, and rural development experts, and to justify their selection based on their experience, community influence, or technical expertise.

The results section would benefit from a comparative analysis between traditional and modern farming practices, highlighting tangible benefits such as increased income, improved market access, resilience to natural disasters, and the preservation of natural resources like water and biodiversity. Additionally, the content analysis of focus group results should be refined to streamline the discussion and better link qualitative quotes to quantitative findings. Including a dedicated section on the study's limitations—such as methodological constraints, data limitations, and the temporal scope—would further enhance the transparency and robustness of the research. Future studies could address these limitations through interdisciplinary approaches and longitudinal designs.

The conclusions and recommendations should be more context-specific, taking into account the territorial and social conditions of the region and the population groups studied. Expanding on how the findings could influence public policies or development programs in the region would significantly enhance the practical relevance of the study.

From a technical perspective, the presentation of Figures 1 and 6 should be revised to improve their clarity and effectiveness. Additionally, the use of more recent or real-time data sources is recommended to improve the accuracy of the findings. Incorporating advanced analytical tools such as GIS, machine learning, or big data analytics could further enhance the study's methodological rigor.

To strengthen the theoretical framework and provide better context for the findings, the following references are suggested:

https://doi.org/10.1016/j.tcrr.2023.03.001

https://doi.org/10.1016/j.ocecoa man.2024.107080

https://doi.org/10.1016/j.geogeo.2022.100129

https://doi.org/10.1007/s42797-022-00059-z

https://doi.org/10.1016/B978-0-323-91603-5.00005-1

This consolidated feedback integrates the reviewers' comments to guide the authors in improving their manuscript, ensuring greater scientific rigor, methodological clarity, and practical relevance.

[Note: HTML markup is below. Please do not edit.]

Reviewers' comments:

Reviewer's Responses to Questions

**Comments to the Author**

1. Is the manuscript technically sound, and do the data support the conclusions?

The manuscript must describe a technically sound piece of scientific research with data that supports the conclusions. Experiments must have been conducted rigorously, with appropriate controls, replication, and sample sizes. The conclusions must be drawn appropriately based on the data presented.

Reviewer #1: Partly

Reviewer #2: Partly

Reviewer #3: Yes

Reviewer #4: Yes

**********

2. Has the statistical analysis been performed appropriately and rigorously?

Reviewer #1: Yes

Reviewer #2: Yes

Reviewer #3: Yes

Reviewer #4: I Don't Know

**********

3. Have the authors made all data underlying the findings in their manuscript fully available?

The PLOS Data policy requires authors to make all data underlying the findings described in their manuscript fully available without restriction, with rare exception (please refer to the Data Availability Statement in the manuscript PDF file). The data should be provided as part of the manuscript or its supporting information, or deposited to a public repository. For example, in addition to summary statistics, the data points behind means, medians and variance measures should be available. If there are restrictions on publicly sharing data—e.g. participant privacy or use of data from a third party—those must be specified.

Reviewer #1: Yes

Reviewer #2: Yes

Reviewer #3: Yes

Reviewer #4: Yes

**********

4. Is the manuscript presented in an intelligible fashion and written in standard English?

PLOS ONE does not copyedit accepted manuscripts, so the language in submitted articles must be clear, correct, and unambiguous. Any typographical or grammatical errors should be corrected at revision, so please note any specific errors here.

Reviewer #1: Yes

Reviewer #2: Yes

Reviewer #3: Yes

Reviewer #4: Yes

**********

5. Review Comments to the Author

Please use the space provided to explain your answers to the questions above. You may also include additional comments for the author, including concerns about dual publication, research ethics, or publication ethics. (Please upload your review as an attachment if it exceeds 20,000 characters)

Reviewer #1: Authors must adress the following suggestion during Revision stage:

• Data Constraints – The study may rely on limited or outdated datasets, which could affect the accuracy of findings. Future research can incorporate real-time or more recent data sources.

Geographical Scope – If the study focuses on a specific region, its findings may not be generalizable to other areas. Expanding the geographic scope can enhance the study's applicability. Must use the following articles for further development:

https://doi.org/10.1016/j.tcrr.2023.03.001

https://doi.org/10.1016/j.ocecoa man.2024.107080

https://doi.org/10.1016/j.geogeo.2022.100129

https://doi.org/10.1007/s42797-022-00059-z

https://doi.org/10.1016/B978-0-323-91603-5.00005-1

• Methodological Limitations – The choice of methodology may have inherent biases or limitations. Using mixed-method approaches or alternative analytical frameworks could yield more robust results.

• Sample Size and Representativeness – A small or non-representative sample may limit the reliability of conclusions. Future research could increase sample size or use stratified sampling techniques.

• Temporal Limitations – The study might be based on a specific time frame, missing seasonal or long-term trends. Longitudinal studies could provide a more comprehensive understanding.

• Lack of Multidisciplinary Perspectives – The study may not incorporate insights from other relevant disciplines such as sociology, economics, or environmental sciences. Interdisciplinary research could strengthen the analysis.

• Policy and Practical Implications – If the study does not sufficiently address policy implications, future research could focus more on actionable recommendations for policymakers and stakeholders.

• Technological and Analytical Tools – The study may not use advanced analytical tools such as machine learning, GIS, or big data analytics, which could enhance data interpretation.

• Social and Cultural Factors – The study may not fully account for socio-cultural influences on the subject matter. Future research could incorporate ethnographic or qualitative approaches to explore these dimensions.

• Unaccounted External Variables – Factors such as climate change, economic fluctuations, or policy shifts may not be fully integrated into the analysis. A more dynamic approach could improve predictive accuracy.

Reviewer #2: I mention my recommendations for adjusting the document:

• The introduction should mention demographic, geographic, social, cultural, and, above all, territorial aspects of Rural Communities in the Lowlands along the Mara River Basin, in order to identify the subject of the research. Although this is mentioned in the methodology, it is important to contextualize the research geographically and socially from the introduction. It is recommended to include a map showing the location of the basin and its rural residents.

• Consider that the deduction made at the end (last paragraph) of the theoretical framework is too ambitious given the number of references mentioned in the same section.

• It mentions that there are some challenges faced, such as environmental degradation, poverty, and unsustainable subsistence practices, but their quantification is not identified. The magnitude of the problem should be quantified from the introduction.

• Conclusions and recommendations should be expressed in a more contextualized manner, taking into account the territorial and social conditions of the geographic area and the population groups being studied (SHF) so that the analysis of the variables is not limited to general assessments.

• The study's limitations should be considered within the conclusions.

• Within the theoretical contribution, the findings should be supported by more previous studies articulated with what is presented in the theoretical framework on the analyzed and correlated variables.

• The content analysis of the focus group results should be improved to streamline the discussion regarding the textual quotations addressed and their comparison with the quantitative results.

• Almost 49% of the textual quotations are from the last 6 years (2020-2025); review this topic without neglecting classic authors.

• There are references in the bibliography that are not cited within the document. It should be verified that each reference in the bibliography complies with all the elements of APA standards.

Reviewer #3: The manuscript is technically sound, the methods used are clear and concise, the statistical analyses used were adequate to present the results, although the style of some graphs is not appropriate in my view, figures 1 and 6 should be presented in another way. Indeed, all relevant data are found within the manuscript. The authors indicate that the data files are found as supplementary information, however I was unable to view them due to the privacy of the participants. Each result is supported by statistical results and a description of the result. The discussion is solid and represents case studies of the area under study. This manuscript represents a significant advance for sustainable livelihoods in the Mara River Basin.

Reviewer #4: The article provides a solid foundation by addressing the complex environmental, social, political, and cultural issues of the Mara River Basin, a region that faces various threats directly impacting the quality of life of its local population. The approach is appropriate as it highlights how these issues are interconnected and affect communities in a cross-cutting manner, especially farmers who depend on the land for their livelihood. The article demonstrates originality by including an analysis of sustainable livelihoods, which is essential for understanding how the local economy can be transformed. However, to strengthen the findings section, it would be interesting to delve deeper into specific points:

- Comparison between traditional and modern practices: Including a comparative analysis of the results obtained by farmers who adopt sustainable practices versus those who follow conventional methods could better illustrate the effectiveness of the new practices.

- Socioeconomic and environmental impact: It would be helpful to detail the tangible benefits these practices have had in terms of income, market access, and resilience to natural disasters. Additionally, highlighting how natural resources (water, biodiversity) are preserved would help better understand the benefits of sustainable livelihoods.

Regarding the methodology, the selection of 265 farmers from the 850 available is a crucial aspect that requires more detail. It is important to specify the following aspects:

- Selection criteria: What specific criteria were used to choose the 265 farmers? It would be helpful to detail whether they were selected based on vulnerability levels, type of crops, or agricultural practices. It would also be valuable to include how diversity regarding gender, age, and socioeconomic status was addressed.

- Justification of the sample: Explain why this number of participants is representative of the total population and how it ensures that the study's conclusions are generalizable to all farmers in the Basin.

The selection of participants for the focus groups is also crucial for the validity of the study. To improve this section, I suggest the following:

- Diversity of stakeholders: Ensure that it is mentioned whether the focus groups included not only farmers but also key actors such as community leaders, representatives from non-governmental organizations, local authorities, and rural development experts. This would ensure a multidimensional perspective on the issue.

- Justification of the selection: Explain why these groups were selected in particular. For example, were they chosen for their field experience, community influence, or technical knowledge on the topics discussed? This would enrich the section and provide greater credibility to the opinions gathered.

Finally, the article rightly emphasizes the importance of developing such research to improve the living conditions of families in the region. To further strengthen this part, it would be helpful to expand on how the research findings could influence public policies or development programs in the region.

**********

6. PLOS authors have the option to publish the peer review history of their article (what does this mean?). If published, this will include your full peer review and any attached files.

If you choose “no”, your identity will remain anonymous but your review may still be made public.

**Do you want your identity to be public for this peer review?** For information about this choice, including consent withdrawal, please see our Privacy Policy.

Reviewer #1: No

Reviewer #2: No

Reviewer #3: No

Reviewer #4: **Yes:** Yojan Gutiérrez-Rojas, Master's degree in Sustainable Productin Systms and Program Advisor in Livelihoods, Norwegian Human Rights Fund.

**********

[NOTE: If reviewer comments were submitted as an attachment file, they will be attached to this email and accessible via the submission site. Please log into your account, locate the manuscript record, and check for the action link "View Attachments". If this link does not appear, there are no attachment files.]

While revising your submission, please upload your figure files to the Preflight Analysis and Conversion Engine (PACE) digital diagnostic tool, https://pacev2.apexcovantage.com/. PACE helps ensure that figures meet PLOS requirements. To use PACE, you must first register as a user. Registration is free. Then, login and navigate to the UPLOAD tab, where you will find detailed instructions on how to use the tool. If you encounter any issues or have any questions when using PACE, please email PLOS at figures@plos.org. Please note that Supporting Information files do not need this step.

Attachment Submitted filename: PONE-D-25-05903.pdf

# Author comment: Author response to Decision Letter 1

**DOI:** 10.1371/journal.pone.0351252.r002

21 Jul 2025

Dear Dr. Juan Carlos Suárez Salazar,

Thank you for the opportunity to revise and resubmit our manuscript to PLOS ONE. We appreciate the constructive feedback provided by the academic editor and reviewers, which has significantly strengthened the manuscript. We have carefully addressed each point raised during the review process, incorporating additional data, refining analyses, and enhancing the clarity and rigor of the manuscript. We outline our responses to the reviewers’ comments, detailing the changes made to the manuscript. We have submitted three files as requested: this rebuttal letter ("Response to Reviewers"), a marked-up copy of the revised manuscript ("Revised Manuscript with Track Changes"), and an unmarked version ("Manuscript"). No changes to the financial disclosure are necessary.

We believe these revisions address all reviewer concerns, enhancing the manuscript’s clarity, depth, and alignment with PLOS ONE’s publication criteria. We are grateful for the reviewers’ insightful feedback, which has significantly improved the quality of our work. Please let us know if further clarifications or adjustments are needed.

Attachment Submitted filename: Response to Reviewers.docx

# Peer review: Decision Letter 1

**DOI:** 10.1371/journal.pone.0351252.r003

**Authors:** Bonoua Faye

3 Nov 2025

PONE-D-25-05903R1

Evaluating Effectiveness of Sustainable Livelihood Development in Rural Communities along Mara River Basin, Tanzania: What Works, What Doesn’t Work, and Why?

PLOS ONE

Dear Dr.  Ngowi,

Thank you for submitting your manuscript to PLOS ONE. After careful consideration, we feel that it has merit but does not fully meet PLOS ONE’s publication criteria as it currently stands. Therefore, we invite you to submit a revised version of the manuscript that addresses the points raised during the review process.

Please submit your revised manuscript by Dec 18 2025 11:59PM. If you will need more time than this to complete your revisions, please reply to this message or contact the journal office at plosone@plos.org. When you're ready to submit your revision, log on to https://www.editorialmanager.com/pone/ and select the 'Submissions Needing Revision' folder to locate your manuscript file.

Please include the following items when submitting your revised manuscript:

- A rebuttal letter that responds to each point raised by the academic editor and reviewer(s). You should upload this letter as a separate file labeled 'Response to Reviewers'.
- A marked-up copy of your manuscript that highlights changes made to the original version. You should upload this as a separate file labeled 'Revised Manuscript with Track Changes'.
- An unmarked version of your revised paper without tracked changes. You should upload this as a separate file labeled 'Manuscript'.

If you would like to make changes to your financial disclosure, please include your updated statement in your cover letter. Guidelines for resubmitting your figure files are available below the reviewer comments at the end of this letter.

If applicable, we recommend that you deposit your laboratory protocols in protocols.io to enhance the reproducibility of your results. Protocols.io assigns your protocol its own identifier (DOI) so that it can be cited independently in the future. For instructions see: https://journals.plos.org/plosone/s/submission-guidelines#loc-laboratory-protocols. Additionally, PLOS ONE offers an option for publishing peer-reviewed Lab Protocol articles, which describe protocols hosted on protocols.io. Read more information on sharing protocols at https://plos.org/protocols?utm_medium=editorial-email&utm_source=authorletters&utm_campaign=protocols.

We look forward to receiving your revised manuscript.

Kind regards,

Bonoua Faye

Academic Editor

PLOS ONE

Journal Requirements:

1. If the reviewer comments include a recommendation to cite specific previously published works, please review and evaluate these publications to determine whether they are relevant and should be cited. There is no requirement to cite these works unless the editor has indicated otherwise.

[Note: HTML markup is below. Please do not edit.]

Reviewer's Responses to Questions

**Comments to the Author**

1. If the authors have adequately addressed your comments raised in a previous round of review and you feel that this manuscript is now acceptable for publication, you may indicate that here to bypass the “Comments to the Author” section, enter your conflict of interest statement in the “Confidential to Editor” section, and submit your "Accept" recommendation.

Reviewer #1: All comments have been addressed

Reviewer #2: All comments have been addressed

Reviewer #3: (No Response)

Reviewer #4: All comments have been addressed

**********

2. Is the manuscript technically sound, and do the data support the conclusions?

The manuscript must describe a technically sound piece of scientific research with data that supports the conclusions. Experiments must have been conducted rigorously, with appropriate controls, replication, and sample sizes. The conclusions must be drawn appropriately based on the data presented.

Reviewer #1: Partly

Reviewer #2: Yes

Reviewer #3: Yes

Reviewer #4: Yes

**********

3. Has the statistical analysis been performed appropriately and rigorously?

Reviewer #1: Yes

Reviewer #2: Yes

Reviewer #3: Yes

Reviewer #4: I Don't Know

**********

4. Have the authors made all data underlying the findings in their manuscript fully available?

The PLOS Data policy requires authors to make all data underlying the findings described in their manuscript fully available without restriction, with rare exception (please refer to the Data Availability Statement in the manuscript PDF file). The data should be provided as part of the manuscript or its supporting information, or deposited to a public repository. For example, in addition to summary statistics, the data points behind means, medians and variance measures should be available. If there are restrictions on publicly sharing data—e.g. participant privacy or use of data from a third party—those must be specified.

Reviewer #1: Yes

Reviewer #2: Yes

Reviewer #3: No

Reviewer #4: Yes

**********

5. Is the manuscript presented in an intelligible fashion and written in standard English?

PLOS ONE does not copyedit accepted manuscripts, so the language in submitted articles must be clear, correct, and unambiguous. Any typographical or grammatical errors should be corrected at revision, so please note any specific errors here.

Reviewer #1: Yes

Reviewer #2: Yes

Reviewer #3: Yes

Reviewer #4: Yes

**********

6. Review Comments to the Author

Please use the space provided to explain your answers to the questions above. You may also include additional comments for the author, including concerns about dual publication, research ethics, or publication ethics. (Please upload your review as an attachment if it exceeds 20,000 characters)

Reviewer #1: Manuscript: PLOS ONE PONE-D-25-05903R1)

Evaluating Effectiveness of Sustainable Livelihood Development in Rural Communities along Mara River Basin, Tanzania: What Works, What Doesn’t Work, and Why?

Following are the suggestions for Major Revision of the Article:

Abstract:

Clarify the research contribution by explicitly stating the evaluation’s novelty or added value in the last two sentences.

Introduction & Theoretical Framework:

Add a sharper research gap statement that explicitly highlights what is underexplored in existing studies, such as the socio-cultural and institutional barriers to livelihood sustainability.

Improve the logical flow by reorganizing content into a clear structure (Context → Challenges → Existing Efforts → Gaps → Aim) while reducing redundancy across paragraphs.

Clarify the study’s broader contribution by explicitly linking its findings to global debates on sustainable livelihoods, climate adaptation, gender equity, and the SDGs.

Methods:

Streamline the methodology by reducing repetition, particularly in the Study Area Description and Justification, to improve clarity and conciseness.

Clarify how the quantitative and qualitative strands of the mixed-methods approach were integrated (e.g., sequential, concurrent, or explanatory) to strengthen methodological transparency.

Explicitly describe the steps taken to ensure data validity and reliability, such as pre-testing surveys, inter-coder checks for qualitative coding, and minimizing respondent bias, to enhance the credibility of findings.

Results & Discussion:

Streamline overlapping explanations by reducing repetition between the Results and Discussion sections, ensuring that the Results focus on findings while the Discussion interprets them.

Strengthen the integration of quantitative and qualitative evidence by explicitly showing how statistical results (e.g., chi-square, ANOVA, regression) align with farmer testimonies or FGD insights.

Improve the flow and readability by organizing findings thematically (e.g., productivity, resilience, gender, markets) rather than mixing statistical tables with narratives in long blocks of text.

Deepen the Discussion by linking findings to broader theoretical debates (e.g., sustainable livelihoods, climate adaptation, gender empowerment) instead of mainly restating results.

Clarify the practical implications of findings for policy and practice by suggesting how to address identified challenges such as market access, policy inconsistencies, and infrastructural gaps.

Conclusion:

Simplify and condense the conclusion by avoiding repetition of results (e.g., yield increase, income diversification, and gender participation are mentioned multiple times). Instead, emphasize the core takeaway message in a sharper, more impactful way.

Strengthen the ending by providing a forward-looking statement that clearly links your recommendations to broader global sustainability agendas (e.g., SDGs, climate resilience frameworks), so the conclusion resonates beyond the Mara River Basin.

Reviewer #2: Kind regards,

I believe that the authors of the manuscript, taking into account most of the observations, demonstrate greater scientific rigor in the research conducted. Therefore, I believe the manuscript can be published in your journal.

Reviewer #3: The figures present the information in a format that does not meet the visual standards expected in high-impact scientific articles (A1). The graph lacks a professional design and adequate editorial quality: the type of power, the edges and the rotation of the labels make reading difficult and do not maintain coherence with the graphic style of the manuscript.

It is recommended to redraw the figure using a statistical analysis or scientific visualization program (for example, R, GraphPad Prism, Origin, Python–Matplotlib), maintaining a uniform scale, legible horizontal labels, and a neutral color palette. Furthermore, it is suggested to include the exact values in it or within the bars only if they are relevant for the interpretation, and avoid text oversaturation

Reviewer #4: The manuscript incorporates the adjustments requested in my review, which contributes to strengthening its coherence, clarity, and academic relevance.

**********

7. PLOS authors have the option to publish the peer review history of their article (what does this mean?). If published, this will include your full peer review and any attached files.

If you choose “no”, your identity will remain anonymous but your review may still be made public.

**Do you want your identity to be public for this peer review?** For information about this choice, including consent withdrawal, please see our Privacy Policy.

Reviewer #1: No

Reviewer #2: **Yes:** CRISTIAN HERNANDEZ GIL

Reviewer #3: No

Reviewer #4: **Yes:** Yojan Gutierrez-Rojas

**********

[NOTE: If reviewer comments were submitted as an attachment file, they will be attached to this email and accessible via the submission site. Please log into your account, locate the manuscript record, and check for the action link "View Attachments". If this link does not appear, there are no attachment files.]

To ensure your figures meet our technical requirements, please review our figure guidelines: https://journals.plos.org/plosone/s/figures

You may also use PLOS’s free figure tool, NAAS, to help you prepare publication quality figures: https://journals.plos.org/plosone/s/figures#loc-tools-for-figure-preparation.

NAAS will assess whether your figures meet our technical requirements by comparing each figure against our figure specifications.

Attachment Submitted filename: Manuscript Revised.docx

# Author comment: Author response to Decision Letter 2

**DOI:** 10.1371/journal.pone.0351252.r004

10 Feb 2026

We are pleased to inform you that we have carefully reviewed and addressed all comments and suggestions provided by the Academic Editor and Reviewers. The revisions undertaken include strengthening the research contribution statement, refining the research gap and theoretical positioning, improving methodological clarity and integration of mixed methods, enhancing results–discussion alignment, improving figure quality and presentation standards, and strengthening the conclusion and policy relevance.

Attachment Submitted filename: Response_to_Reviewers_auresp_2.docx

# Editor report: Decision Letter 2

**DOI:** 10.1371/journal.pone.0351252.r005

**Authors:** Bonoua Faye

4 Mar 2026

PONE-D-25-05903R2

Evaluating Effectiveness of Sustainable Livelihood Development in Rural Communities along Mara River Basin, Tanzania: What Works, What Doesn’t Work, and Why?

PLOS One

Dear Dr. Ngowi,

Thank you for submitting your manuscript to PLOS ONE. After careful consideration, we feel that it has merit but does not fully meet PLOS ONE’s publication criteria as it currently stands. Therefore, we invite you to submit a revised version of the manuscript that addresses the points raised during the review process.

Please submit your revised manuscript by Apr 18 2026 11:59PM. If you will need more time than this to complete your revisions, please reply to this message or contact the journal office at plosone@plos.org. When you're ready to submit your revision, log on to https://www.editorialmanager.com/pone/ and select the 'Submissions Needing Revision' folder to locate your manuscript file.

Please include the following items when submitting your revised manuscript:

- A letter that responds to each point raised by the academic editor and reviewer(s). You should upload this letter as a separate file labeled 'Response to Reviewers'.
- A marked-up copy of your manuscript that highlights changes made to the original version. You should upload this as a separate file labeled 'Revised Manuscript with Track Changes'.
- An unmarked version of your revised paper without tracked changes. You should upload this as a separate file labeled 'Manuscript'.

-->If you would like to make changes to your financial disclosure, please include your updated statement in your cover letter. Guidelines for resubmitting your figure files are available below the reviewer comments at the end of this letter.

If applicable, we recommend that you deposit your laboratory protocols in protocols.io to enhance the reproducibility of your results. Protocols.io assigns your protocol its own identifier (DOI) so that it can be cited independently in the future. For instructions see: https://journals.plos.org/plosone/s/submission-guidelines#loc-laboratory-protocols. Additionally, PLOS ONE offers an option for publishing peer-reviewed Lab Protocol articles, which describe protocols hosted on protocols.io. Read more information on sharing protocols at https://plos.org/protocols?utm_medium=editorial-email&utm_source=authorletters&utm_campaign=protocols.

We look forward to receiving your revised manuscript.

Kind regards,

Bonoua Faye

Academic Editor

PLOS One

Journal Requirements:

If the reviewer comments include a recommendation to cite specific previously published works, please review and evaluate these publications to determine whether they are relevant and should be cited. There is no requirement to cite these works unless the editor has indicated otherwise.

Please review your reference list to ensure that it is complete and correct. If you have cited papers that have been retracted, please include the rationale for doing so in the manuscript text, or remove these references and replace them with relevant current references. Any changes to the reference list should be mentioned in the rebuttal letter that accompanies your revised manuscript. If you need to cite a retracted article, indicate the article’s retracted status in the References list and also include a citation and full reference for the retraction notice.

Additional Editor Comments (if provided):

Dear authors

While the manuscript addresses an important topic and uses an appropriate mixed-methods design, revisions are still needed. For instance, the Sustainable Livelihoods Framework is introduced but not consistently applied in the discussion, limiting analytical depth. Several figures and tables are difficult to interpret, and statistical reporting is inconsistent, for example, in p-value formats.

The discussion is overly long and repeats results rather than engaging with prior literature or clearly articulating contributions. Finally, kindly add more practical recommendations.

Language polishing and reference checking, such as DOI, are required.

In addition, such a title, “5.3 Why? should be improved more smoothly.

For “2. The Theoretical Framework,” kindly add the framework showing the interconnection between the main concepts.

Add the map of the study area.

Sincerely,

[Note: HTML markup is below. Please do not edit.]

[NOTE: If reviewer comments were submitted as an attachment file, they will be attached to this email and accessible via the submission site. Please log into your account, locate the manuscript record, and check for the action link "View Attachments". If this link does not appear, there are no attachment files.]

To ensure your figures meet our technical requirements, please review our figure guidelines: https://journals.plos.org/plosone/s/figures

You may also use PLOS’s free figure tool, NAAS, to help you prepare publication quality figures: https://journals.plos.org/plosone/s/figures#loc-tools-for-figure-preparation.

NAAS will assess whether your figures meet our technical requirements by comparing each figure against our figure specifications.

# Author comment: Author response to Decision Letter 3

**DOI:** 10.1371/journal.pone.0351252.r006

10 Apr 2026

Dear Reviewer,

We sincerely thank you for your thoughtful and constructive comments on our manuscript. Your insights were invaluable in guiding the revision process. Thank you for this important comment. The manuscript has been thoroughly revised to comply with PLOS ONE referencing requirements using the Vancouver citation style. Specifically, (i) all references have now been compiled into a numbered reference list placed at the end of the manuscript, (ii) in-text citations have been converted from author–date format to numerical format in the order of appearance, and (iii) DOIs have been added where available. All citations have been cross-checked to ensure consistency between in-text numbering and the reference list. Below, we provide a point-by-point response to each comment, describing the actions we took to address your concerns. All changes were made to the revised manuscript.

Reviewer Comment 1:

The Sustainable Livelihoods Framework is introduced but not consistently applied in the discussion, limiting analytical depth.

Author Response:

We agreed with this observation. We therefore restructured the entire Discussion section (Section 4) to explicitly apply the Sustainable Livelihoods Framework (SLF) to each major finding. We reorganized the discussion under three sub-headings that directly map onto the SLF components: “What Works? Interpreting Successes through the SLF” (linking findings to Livelihood Assets), “What Doesn't Work? Systemic Barriers as Failures of Transforming Structures” (linking to Transforming Structures and Processes), and “Theoretical and Practical Contributions.” Each paragraph now explicitly names the relevant SLF concept (e.g., human capital, social capital, physical capital, transforming structures) and explains how the finding supports or challenges the framework.

Location of Change in Manuscript:

Section 4 (Discussion), sub-sections 4.1, 4.2, and 4.3 (pages 28-31 of the revised manuscript).

Reviewer Comment 2:

Several figures and tables are difficult to interpret, and statistical reporting is inconsistent, for example, in p-value formats.

Author Response:

We accepted this criticism and took the following actions. First, we standardized all p-value reporting throughout the manuscript. All p-values greater than 0.001 were reported as exact values (e.g., p = 0.03, p = 0.004), while values below 0.001 were reported as p < 0.001. Second, we revised the titles of several tables and figures to make them more descriptive and self-explanatory. For example, Table 7 was retitled from “Paired t-Test Results for Agricultural Yield Changes” to “Impact of MFEC Training on Agricultural Yield: A Paired t-Test Comparison of Before-and-After Intervention (n=265)”. Similarly, Figure 6 was retitled from “Effectiveness of Climate Adaptation Practices Adopted by SHFs” to “Smallholder Farmer Perceptions of the Effectiveness of Different Climate Adaptation Practices (n=265)”.

Location of Changes in Manuscript:

• Standardized p-values: Throughout the Results (Section 4) and Discussion (Section 5) sections.

• Table 7: Page 18

• Figure 6: Page 21

Reviewer Comment 3:

The discussion is overly long and repeats results rather than engaging with prior literature or clearly articulating contributions.

Author Response:

We agreed with this assessment. We therefore substantially shortened and refocused the Discussion section. We removed all repetitive content that merely restated results from Section 4. Instead, we reframed the discussion to interpret the meaning of the key findings, explicitly compared and contrasted our results with the cited literature (e.g., FAO, 2020; Barrett, 2020; Kabeer, 2021), and added a new, dedicated sub-section titled “Theoretical and Practical Contributions” (Section 4.3). This new sub-section clearly articulates the study’s two main contributions: empirical validation of the SLF in a data-scarce context and the provision of a replicable, low-cost evaluation protocol for practitioners.

Location of Change in Manuscript:

Section 4 (Discussion), entirely replaced with new text on pages 28-31.

Reviewer Comment 4:

Kindly add more practical recommendations.

Author Response:

We agreed that the original recommendations were too general. We therefore added a new, numbered sub-section titled “Specific, Actionable Recommendations for Policy and Practice” at the end of the Conclusions section. These recommendations were organized by target audience (District/National Government, Implementing NGO, Development Partners) and were made specific, measurable, and directly tied to the study’s findings (e.g., creating a voucher scheme for organic inputs to address policy confusion, developing a revolving fund for beekeeping protective gear based on the correlation β = +0.42, *p* < 0.05).

Location of Change in Manuscript:

Section 5 (Conclusions and Recommendations), added as a new sub-section before “Funding” (pages 34-35 of the revised manuscript).

Reviewer Comment 5:

Language polishing and reference checking, such as DOI, are required.

Author Response:

We took two actions to address this comment. First, we arranged for professional language editing of the entire manuscript, focusing on sentence structure, passive voice, and consistency in terminology (e.g., changing “Low Land” to “Lowland”). Second, we verified every reference in the bibliography and added Digital Object Identifiers (DOIs) where available. For example, we added DOIs for Bowen (2009), Creswell & Plano Clark (2018), and Scoones (2009), among others.

Location of Change in Manuscript:

• Language polishing: Throughout the entire manuscript.

• References with added DOIs: Section “References” (pages 37-44).

Reviewer Comment 6:

Such a title, “5.3 Why?” should be improved more smoothly.

Author Response:

We agreed that the original sub-section title was too abrupt. We therefore revised it to a more descriptive and academically professional title. The new title clearly indicates the content of the sub-section.

Location of Change in Manuscript:

Section 5.3. The title was changed from “Why?” to “Understanding the Underlying Drivers: Why Barriers Persist and Successes Occur” (page 32).

Reviewer Comment 7:

For “2. The Theoretical Framework,” kindly add the framework showing the interconnection between the main concepts.

Author Response:

We accepted this suggestion. We therefore created a new conceptual diagram (Image 1) that visually synthesizes the interconnections among the core components of the Sustainable Livelihoods Framework as applied to this project. The figure is a flow diagram showing the relationships among the Vulnerability Context, Livelihood Assets (human, social, natural, physical, financial), Transforming Structures and Processes, and Livelihood Strategies and Outcomes. An explanatory paragraph was added immediately preceding the figure.

Location of Change in Manuscript:

Section 2 (The Theoretical Framework), at the end of the section, just before Section 3 (Methodology) (page 7).

Reviewer Comment 8: Add the map of the study area.

Author Response:

We noted that a map (Image 2) was already present in the original manuscript. However, to improve its interpretability as suggested, we revised the map caption to be more detailed and descriptive. The new caption explicitly names the ten project villages, the five wards, and the position of the Mara River Basin within East Africa.

Location of Change in Manuscript: Section 3.1 (Study Area Description), caption for Image 1 (page 8).

Summary of Changes

We believe that the revisions made in response to your comments have substantially improved the manuscript. The theoretical framework is now consistently applied, the data presentation is clearer, the discussion is focused and contributes to the literature, the recommendations are actionable, and the technical quality of the writing and references has been enhanced. We are grateful for your time and expertise in reviewing our work.

We look forward to your final decision.

Sincerely,

Edwin E. Ngowi and Angela M. Jesse

Attachment Submitted filename: Response_to_Reviewers_auresp_3.docx

# Editor report: Decision Letter 3

**DOI:** 10.1371/journal.pone.0351252.r007

**Authors:** Sandra Ricart

25 May 2026

<p>Evaluating Effectiveness of Sustainable Livelihood Development in Rural Communities along Mara River Basin, Tanzania: What Works, What Doesn’t Work, and Why?

PONE-D-25-05903R3

Dear Dr. Ngowi,

We’re pleased to inform you that your manuscript has been judged scientifically suitable for publication and will be formally accepted for publication once it meets all outstanding technical requirements.

Within one week, you’ll receive an e-mail detailing the required amendments. When these have been addressed, you’ll receive a formal acceptance letter and your manuscript will be scheduled for publication.

An invoice will be generated when your article is formally accepted. Please note, if your institution has a publishing partnership with PLOS and your article meets the relevant criteria, all or part of your publication costs will be covered. Please make sure your user information is up-to-date by logging into Editorial Manager at Editorial Manager® and clicking the ‘Update My Information' link at the top of the page. For questions related to billing, please contact billing support.

If your institution or institutions have a press office, please notify them about your upcoming paper to help maximize its impact. If they’ll be preparing press materials, please inform our press team as soon as possible -- no later than 48 hours after receiving the formal acceptance. Your manuscript will remain under strict press embargo until 2 pm Eastern Time on the date of publication. For more information, please contact onepress@plos.org.

Kind regards,

Sandra Ricart

Academic Editor

PLOS One

# Editor report: Acceptance letter

**DOI:** 10.1371/journal.pone.0351252.r008

**Authors:** Sandra Ricart

PONE-D-25-05903R3

PLOS One

Dear Dr. Ngowi,

I'm pleased to inform you that your manuscript has been deemed suitable for publication in PLOS One. Congratulations! Your manuscript is now being handed over to our production team.

At this stage, our production department will prepare your paper for publication. This includes ensuring the following:

* All references, tables, and figures are properly cited

* All relevant supporting information is included in the manuscript submission,

* There are no issues that prevent the paper from being properly typeset

You will receive further instructions from the production team, including instructions on how to review your proof when it is ready. Please keep in mind that we are working through a large volume of accepted articles, so please give us a few days to review your paper and let you know the next and final steps.

Lastly, if your institution or institutions have a press office, please let them know about your upcoming paper now to help maximize its impact. If they'll be preparing press materials, please inform our press team within the next 48 hours. Your manuscript will remain under strict press embargo until 2 pm Eastern Time on the date of publication. For more information, please contact onepress@plos.org.

You will receive an invoice from PLOS for your publication fee after your manuscript has reached the completed accept phase. If you receive an email requesting payment before acceptance or for any other service, this may be a phishing scheme. Learn how to identify phishing emails and protect your accounts at https://explore.plos.org/phishing.

If we can help with anything else, please email us at customercare@plos.org.

Thank you for submitting your work to PLOS ONE and supporting open access.

Kind regards,

PLOS ONE Editorial Office Staff

on behalf of

Prof. Sandra Ricart

Academic Editor

PLOS One
